# Supplementary material for: Ligand engineering enhances (photo) electrocatalytic activity and stability of zeolitic imidazolate frameworks via in-situ surface reconstruction
Source: Nat Commun. 2024 Oct 30;15:9393. doi: 10.1038/s41467-024-53385-0 (PMC11526130; doi:10.1038/s41467-024-53385-0)
Supplement: Supplementary file 1 — Supplementary Information [file 41467_2024_53385_MOESM1_ESM.pdf]

Supporting Information

Ligand Engineering Enhances (Photo)Electrocatalytic Activity and Stability of Zeolitic Imidazolate Frameworks via In-situ Surface Reconstruction

Zheao Huang [1], Zhouzhou Wang [2], Hannah Rabl [1], Shaghayegh Naghdi [1], Qiancheng Zhou [2], Sabine Schwarz [3], Dogukan Hazar Apaydin [1], Ying Yu [,2] and Dominik Eder [*,1]*

[1] Institute of Materials Chemistry, Technische Universität Wien, 1060, Vienna, Austria

[2] Institute of Nanoscience and Nanotechnology, College of Physical Science and Technology, Central China Normal University, 430079, Wuhan, China

[3] Service Center for Electron Microscopy (USTEM), Technische Universität Wien, 1040, Vienna, Austria

* Corresponding authors: dominik.eder@tuwien.ac.at and yuying01@ccnu.edu.cn

Content

S1. Structural characterization of LE-ZIFs 3

S2. Electrocatalytic studies 10

S3. Structure evolution after electrocatalytic reaction 22

S4. Evolution of morphology and elemental distribution after electrocatalytic reaction 36

S5. In-situ UV–Vis absorption and Raman spectroscopy during electrocatalytic reactions......... 44

S6. Photoelectrocatalysis and band gaps calculation 49

S7. DFT simulation calculations 58

S8. Characterizations Section 68

S9. References 70

S1. Structural characterization of LE-ZIFs

The XRD patterns of LE-ZIFs, incorporating various secondary ligands, exhibit well-maintained crystallinity with Bragg peaks corresponding to A-ZIF (ZIF-67 in Fig. S1a). The introduction of secondary ligands occurred in a one-to-one weight ratio during the synthesis process, i.e., the stations have a 50 wt.%. However, due to competitive coordination among ligands, the actual content of the secondary ligand deviates from the nominal values, as detailed in the ^{1}H NMR analysis (Table S3). A comparison of the XRD patterns of as-prepared ZIFs reveals that the impact of different ligands on the positions of the (011)/(112) peaks is not significant in LE-ZIFs compared to the single-ligand ZIF-67 (Fig. S1b). The SEM images of as-prepared ZIFs exhibit typical ZIF-67 morphology, characterized by dodecahedral particles (Fig. S2). With the exception of a size increase in AB-ZIF, the particle sizes of other LE samples closely resemble those of A-ZIF, around 350 nm.

In the ATR-IR spectra of as-prepared ZIFs, distinct from A-ZIF, the incorporation of secondary ligands results in the appearance of new bands in the range of 472 to 505 cm^{-1} (Fig. S3). These bands are located at 472 cm^{-1} (B), 461 cm^{-1} (C), 499 cm^{-1} (D), and 505 cm^{-1} (E), respectively, closely resembling the Co-N$_\alpha$ band (435 cm^{-1}) of the original ligand (A). Based on the conclusions drawn from previous reports on mixed-ligand ZIFs [1, 2, 3], these new bands are attributed to the bonding of the secondary ligands with metal ions, referred to herein as Co-N$_\beta$. The emergence of the Co-N$_\beta$ bands signify the achievement of ligand substitution process through the successful integration of the secondary ligands into the ZIF-67 framework. Influence by the secondary ligands, the LE-ZIFs exhibit notable differences in the imidazole ring stretching region (700 to 1500 cm^{-1}) compared to A-ZIF.

Through N_2 physisorption at 77 K, as-prepared ZIFs exhibit type-I isotherms, characteristic of typical microporous materials (Fig. S4). Utilizing nonlocal density functional theory (NLDFT), we observed that the distinct peak centered at 1.2 nm, representative of intrinsic micropores, is largely preserved independent of the initial ligand mixing. Interestingly, samples AC and AD exhibit additional pores in the range from 2 to 20 nm (Fig. S5), possibly influenced by incorporation of secondary ligands, a phenomenon commonly observed in previous literature [3, 4]. However, their micropore volume still predominates in total pore distribution. Compared to A-ZIF (2291.3 m^2 g^{-1}), other LE-ZIFs exhibit varying degrees of decrease in Brunauer-Emmett-Teller (BET) specific surface area due to the disorder and bond stretching caused by the secondary ligands [5, 6]. Specifically, AB-ZIF has a surface area of 1092.9 m^2 g^{-1}, AC-ZIF has 717.8 m^2 g^{-1}, AD-ZIF has 860.6 m^2 g^{-1}, and AE-ZIF has 807.7 m^2 g^{-1} (Table S1).

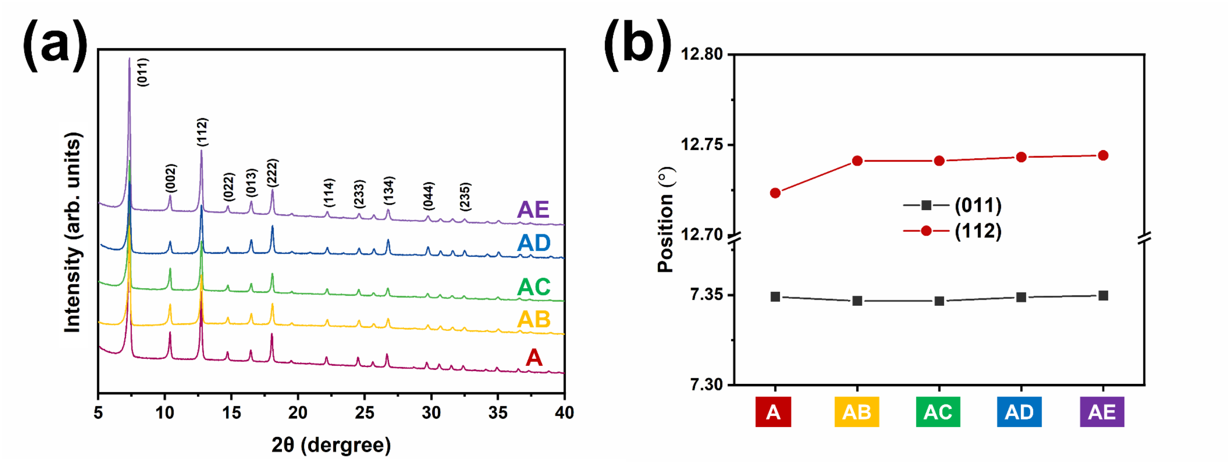

Fig. S1. **a**, XRD patterns of A-ZIF, AB-ZIF, AC-ZIF, AD-ZIF and AE-ZIF. **b**, Evolution plots of (011)/(112) peak position in A-ZIF, AB-ZIF, AC-ZIF, AD-ZIF and AE-ZIF.

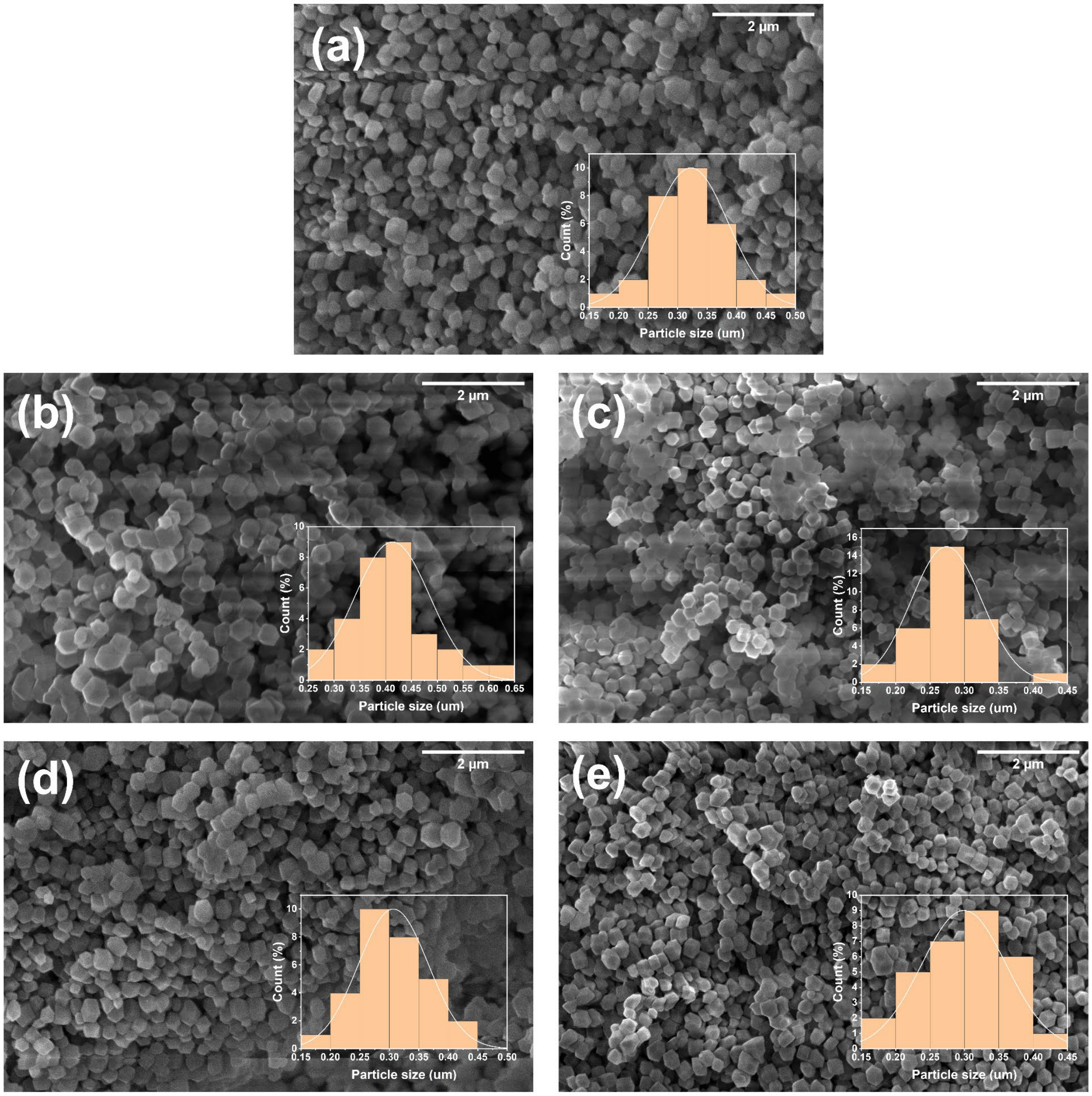

Fig. S2. SEM images of A-ZIF (**a**), AB-ZIF (**b**), AC-ZIF (**c**), AD-ZIF (**d**) and AE-ZIF (**e**). An ImageJ program was used to select 30 particles for particle size statistics from each sample.

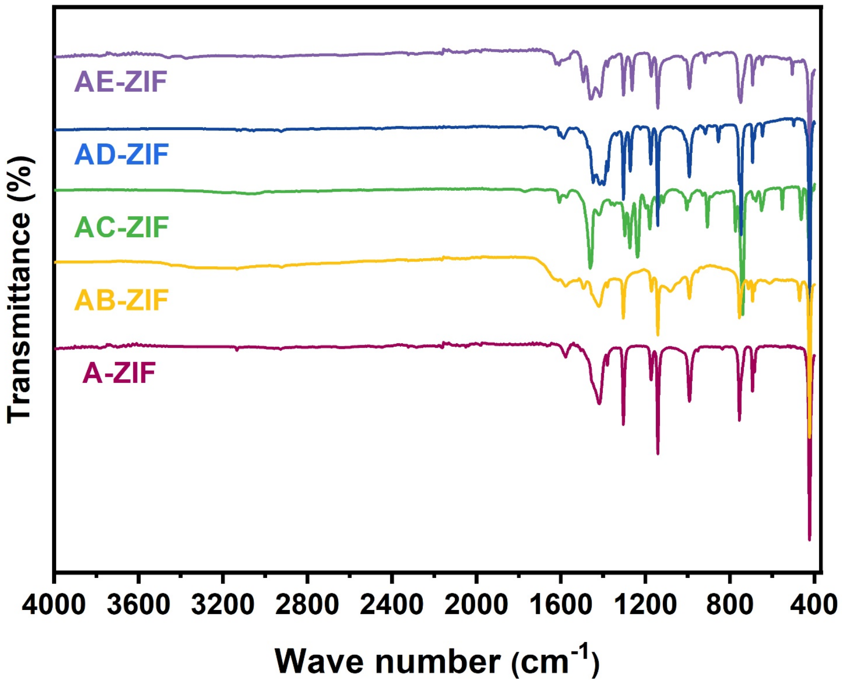

Fig. S3. ATR-IR spectra of A-ZIF, AB-ZIF, AC-ZIF, AD-ZIF and AE-ZIF.

Table S1. Specific surface area and porosity parameters of as-prepared ZIFs.

**Samples**	**Specific surface area[a] ($m^2\ g^{-1}$)**	**Total Pore Volume ($cm^3\ g^{-1}$)**	**Micropore Volume[b] ($cm^3\ g^{-1}$)**	**Total Area in Pores ($m^2\ g^{-1}$)**
A-ZIF	1867.9	0.655	0.647	1977.7
AB-ZIF	1092.9	0.489	0.424	1571.2
AC-ZIF	717.8	0.456	0.213	902.5
AD-ZIF	860.6	0.482	0.292	1214.8
AE-ZIF	891.3	0.365	0.320	1236.0

[a] Brunauer-Emmett-Teller specific surface area.

[b] Cumulative adsorption volume of micropores from 0 to 2 nm in diameter in diameter according to International Union of Pure and Applied Chemistry (IUPAC) [7].

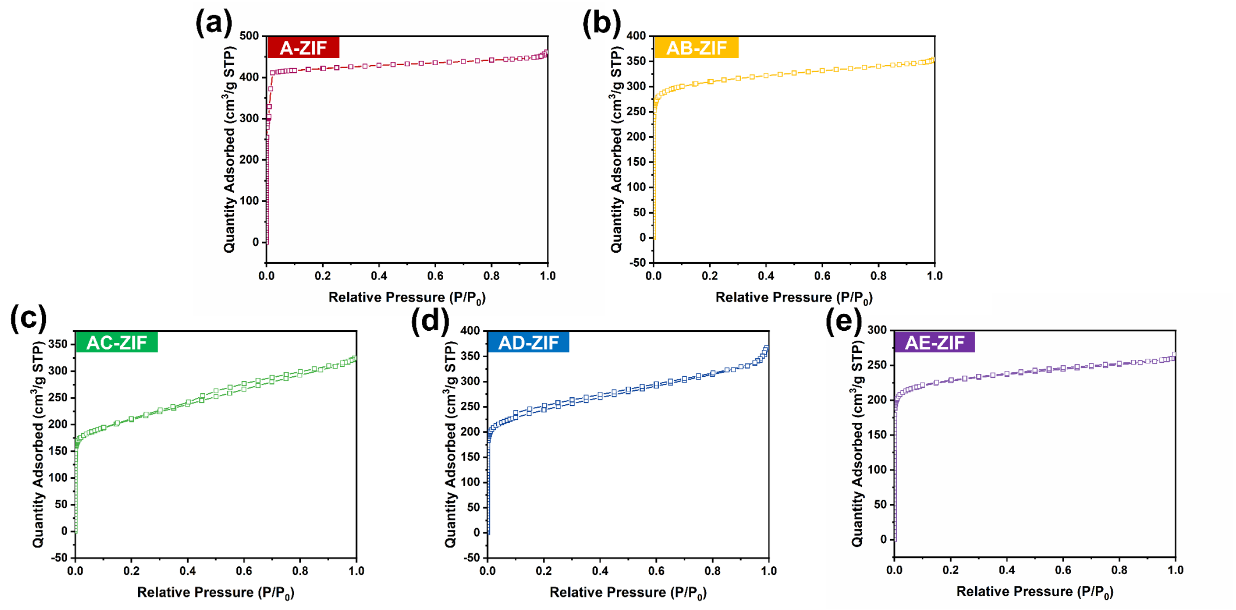

Fig. S4. N_2 sorption isotherm of A-ZIF (**a**), AB-ZIF (**b**), AC-ZIF (**c**), AD-ZIF (**d**) and AE-ZIF (**e**).

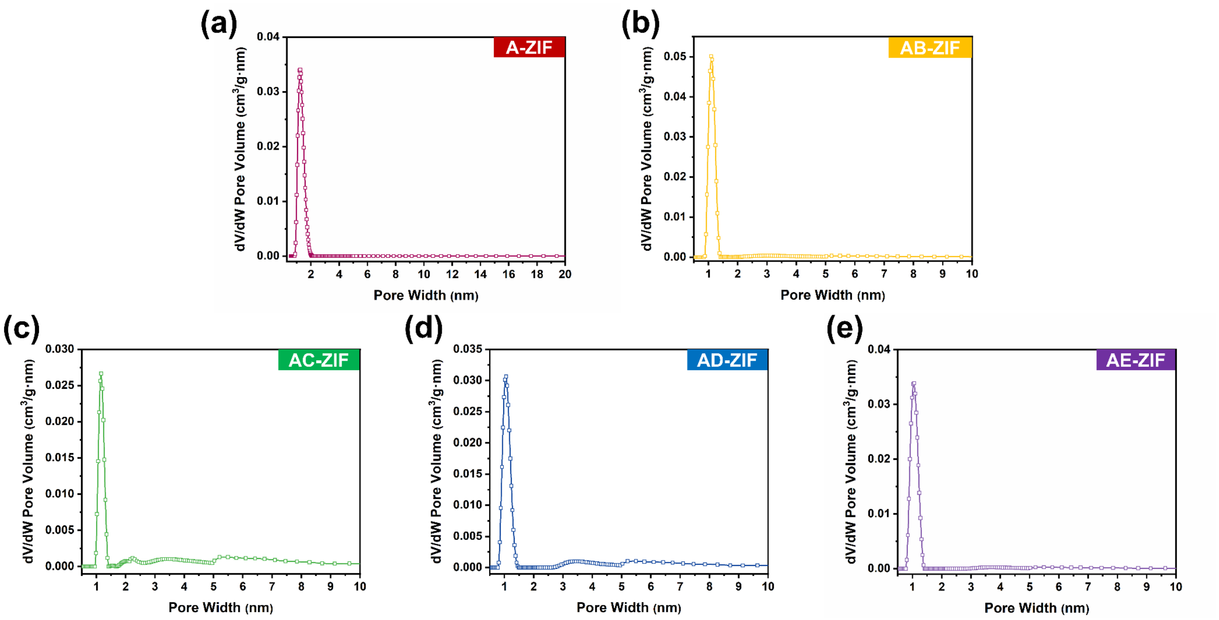

Fig. S5. NLDFT porosity distributions of A-ZIF (**a**), AB-ZIF (**b**), AC-ZIF (**c**), AD-ZIF (**d**) and AE-ZIF (**e**).

S2. Electrocatalytic studies

The following potentials are all referenced to the reversible hydrogen electrode (RHE). As shown in Fig. S7a, AB-ZIF exhibits a continuous decrease from the beginning of the CV cycles until stabilizing at 20 cycles. Similarly, the charge of $R_{LE\text{-}AB}1$ rapidly decreases within the first 10 CV cycles, suggesting that the framework structure of AB-ZIF had been completely degraded (Fig. S8b). The CR process of AB-ZIF at low CV cycles leads to a rapid increase in the overpotential and Tafel slope of unstable AB-ZIF within the first 20 cycles (Fig. S11a), indicating a quickly response of its electrocatalytic rate to potential changes [8]. All these findings collectively indicate the poor stability of AB-ZIF in electrocatalytic reaction.

After 60 CV cycles, the current density of AC-ZIF slightly decreases, and its overpotential exhibits an inverted volcano-shaped curve, decreasing initially and then increasing (Figs. S7b and S11b). In contrast, the current density, overpotential, and Tafel slope of AD-ZIF show no significant changes after 80 CV cycles, remaining relatively stable (Figs. S7c and S11c). These observations indicate that AC-ZIF is more strongly influenced by the electro-oxidation process compared to AD-ZIF during electrocatalysis.

Since electrochemically active surface area (ECSA) is directly proportional to the electrochemical double-layer capacitance (C_{dl}), which was estimated based on the non-Faradaic region of the cyclic voltammetry (CV) curves at different scan rates (20 to 100 mV s^{-1}; see Fig. S9a-e). As shown in Figs. S9c and S9f, before the electrocatalytic activation, A-ZIF and AE-ZIF exhibited similar ECSA values of 64.25 cm^{-2} and 78.00 cm^{-2}, respectively. However, after 100 cycles, the ECSA of A-ZIF decreased significantly to 33.25 cm^{-2} due to its own complete reconstruction, representing a reduction of about 48%. In contrast, AE-ZIF, which experienced only surface reconstruction, maintained a high ECSA value of 71.50 cm^{-2}, showing a minor decrease of 8%. These conclusions are consistent with our TEM analysis of the two samples. The degradation of the surface area of the AE-ZIF particles caused by the 5 nm thick cobalt (oxy)hydroxide layer (Fig. 4) corresponds exactly to a decrease of 8% ECSA, while the decrease of 48% ECSA for A-ZIF corresponding to the transformation of particles into nanosheets.

In a 12-hour amperometry at 1.65V, sample AB exhibits severe current fluctuations (Fig. S13a). Its current density significantly decreases within the initial 2 hours, while the O_2 evolution rate initially increases before declining and final loss of 79.8%, consistent with the conclusions in the CV analysis. AC-ZIF loses 21.4% of the O_2 evolution rate after 12 hours, indicating poorer stability than AD and AE (Fig. S13b). The current density trend of AD-ZIF is similar to AE-ZIF, rapidly increasing in the

initial 2 hours and then stabilizing (Fig. S13c). This indicates that their electrodes only undergo rapid reconstruction in the initial 2 hours, and have higher stability in electrochemical performance compared to other ZIF samples. However, both AC and AD do not perform as well as AE-ZIF, exhibiting only about one- eighth of the performance in terms of current density and O_2 evolution rate.

Under intermittent biased conditions, after stopping and restarting the application of 1.65 V, the current density and O_2 evolution rate of AE-ZIF quickly returned to their original levels and remained stable (Fig. S13d). Unlike the initial three hours, AE-ZIF did not exhibit a gradual increase in current density due to surface reconstruction again, indicating that AE-ZIF formed a stable cobalt (oxy)hydroxide layer within the first two hours. This layer effectively inhibited further electro-oxidation process, achieving in a structurally stable state.

The electrical conductivity of each ZIF@FTO glass was evaluated using the two-probe method, where the initial conductivity of AC, AD and AE surpass that of A and AB. This improvement can be attributed to the incorporation of π-π stacking aromatic carbon rings in the secondary ligands (C, bIm; D, 2-bIm; E, NH_2-bIm), which function as additional conductive pathway, effectively facilitating electron transfer [9]. With the increase in the number of CV cycles at high potential window, the electrical conductivity for each ZIF@FTO exhibit discernible changes and closely mirroring with the trends in current density, indicating a strong correlation between OER activity and conductivity (Fig. S14). Apart from AB-ZIF, other ZIFs show varying degrees of conductivity increase, typically attributed to the formation of high-valence Co species and ligand shedding. Sample AE-ZIF@FTO exhibits the highest electrical conductivity among other LE samples, reaching approximately −1.0 (log S/cm) after CV cycles. On the other hand, the trends in electrochemical impedance spectroscopy (EIS) are less pronounced in the CV cycles, as illustrated in Fig. S15.

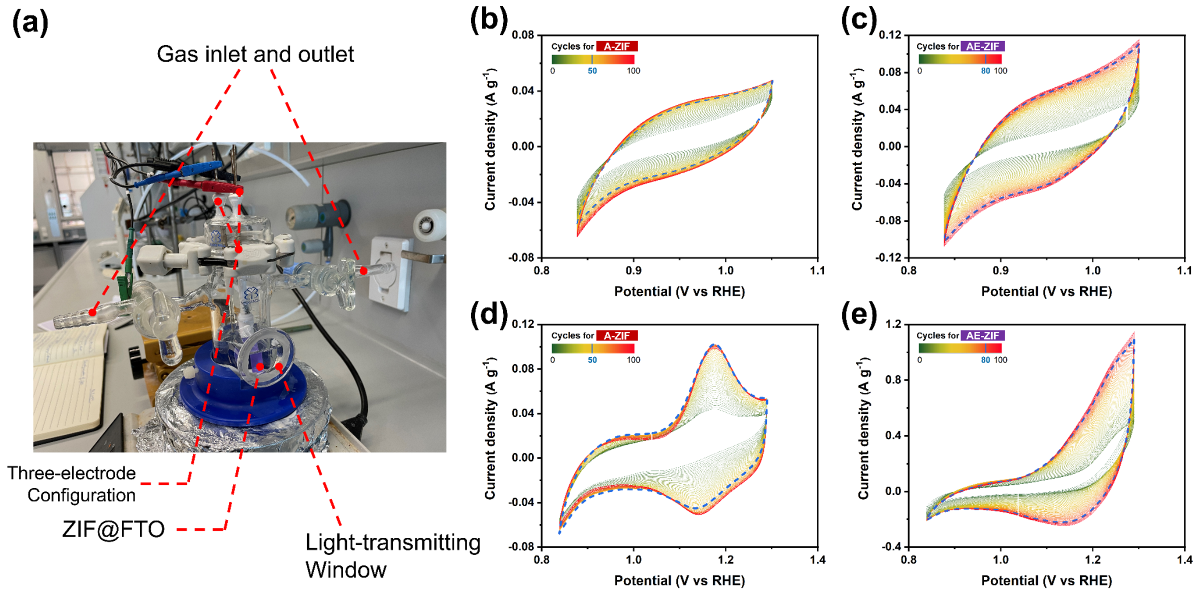

Fig. S6. a, Experimental setup for a standard three-electrode configuration used in electrocatalytic measurements. **b**, **c**, Continuous cyclic voltammograms at low potential window between 0.85 to 1.05 V of A-ZIF (**b**) and AE-ZIF (**c**). **d**, **e**, At medium potential window between 0.85 to 1.30 V of A-ZIF (**d**) and AE-ZIF (**e**).

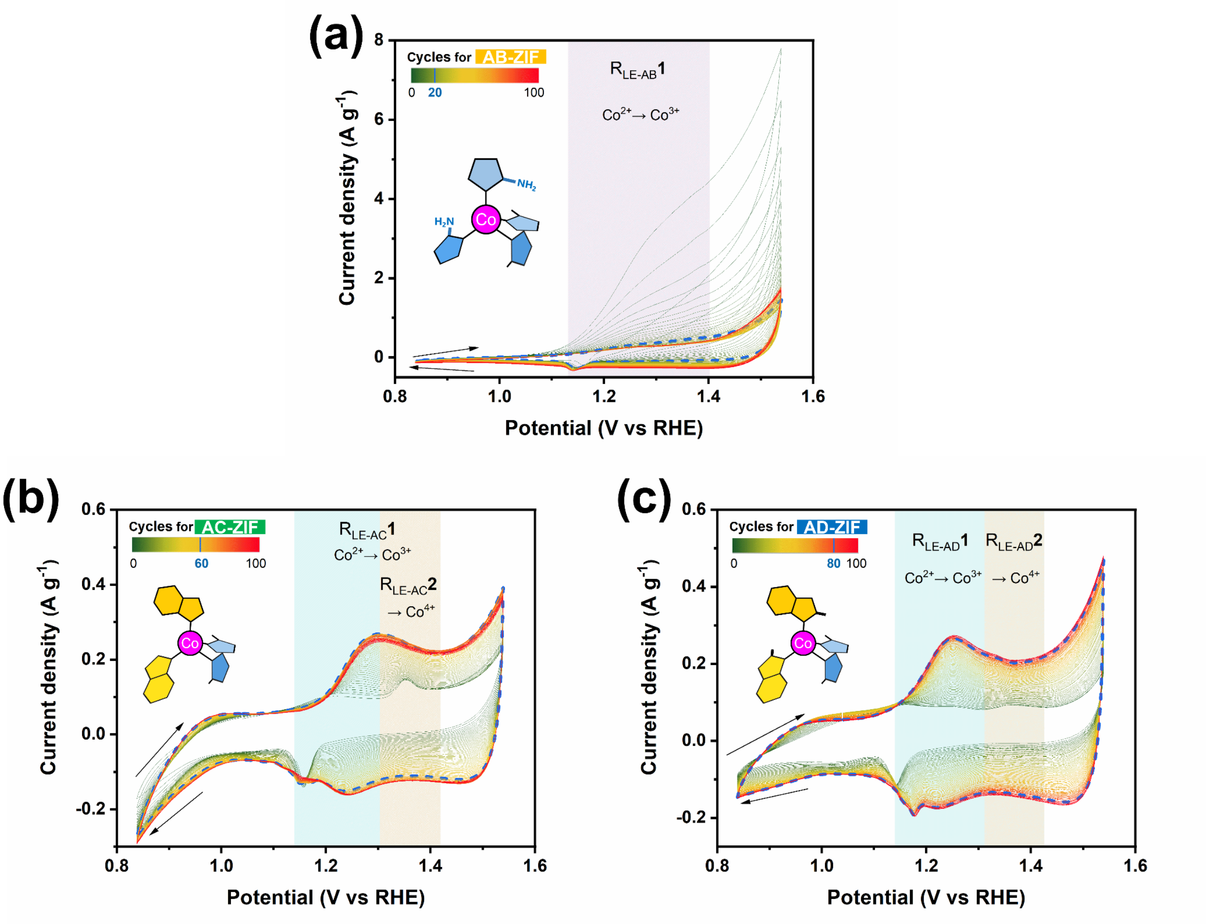

Fig. S7. Continuous cyclic voltammograms at higher potential window between 0.85 to 1.55 V of AB-ZIF (**a**), AC-ZIF (**b**) and AD-ZIF (**c**). Dashed line is the current density maximum, the scan rate is 10 mV s^{-1}.

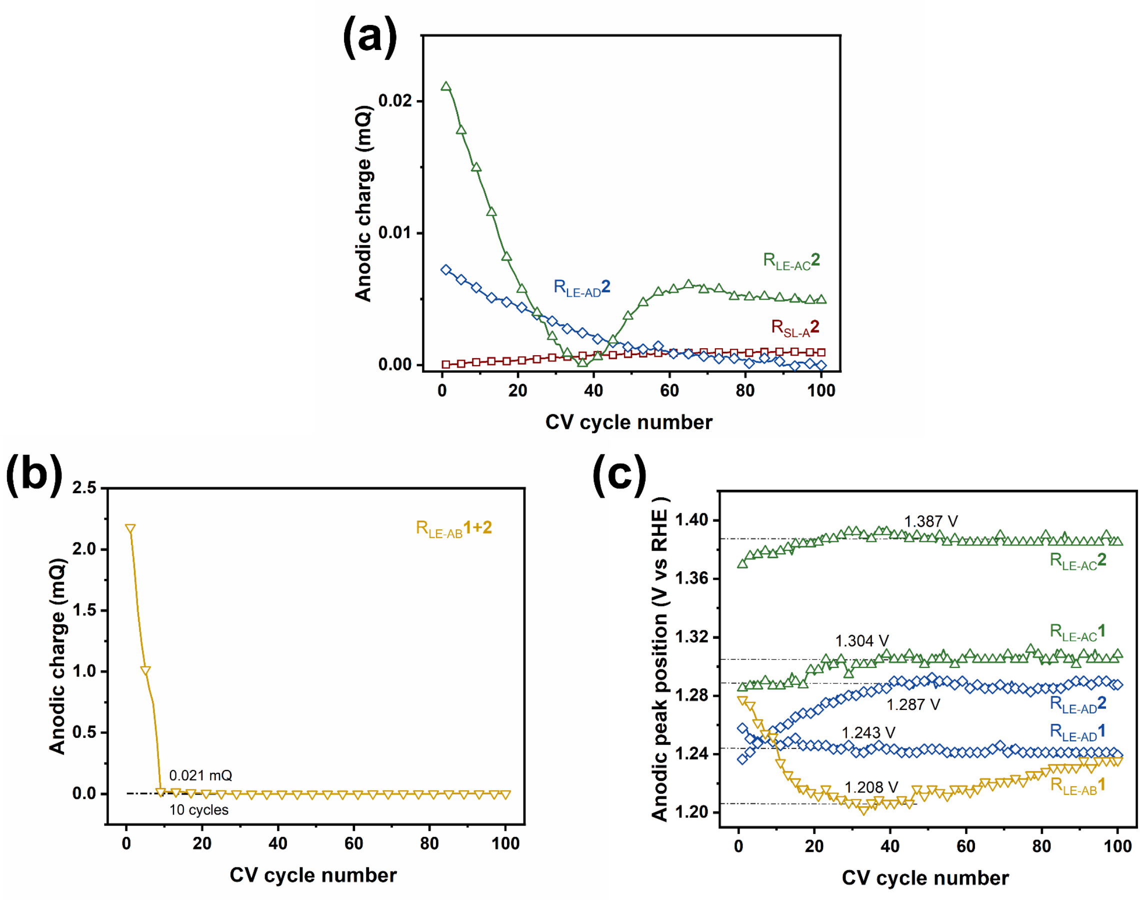

Fig. S8. **a**, **b**, Anodic charge plots with CV cycles of A-ZIF, AC-ZIF, AD-ZIF (**a**) and AB-ZIF (**b**). **c**, Peak position plots with CV cycles of AB-ZIF, AC-ZIF and AD-ZIF.

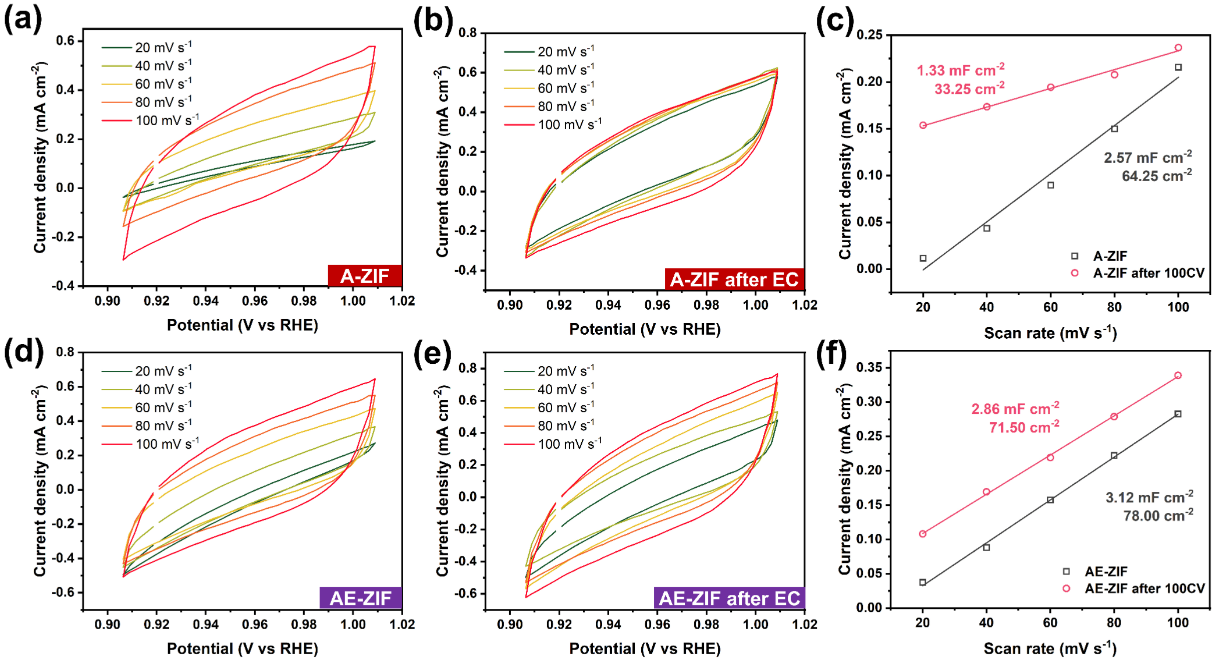

Fig. S9. a, **b**, **d**, **e**, Cyclic voltammetry curves of A-ZIF (**a**), A-ZIF after 100 CV (**b**), AE-ZIF (**d**) and AE-ZIF after 100 CV (**e**) at different scan rates in 1.0 M KOH. **c**, **f**, Capacitive currents of A-ZIF (**c**) and AE-ZIF (**f**).

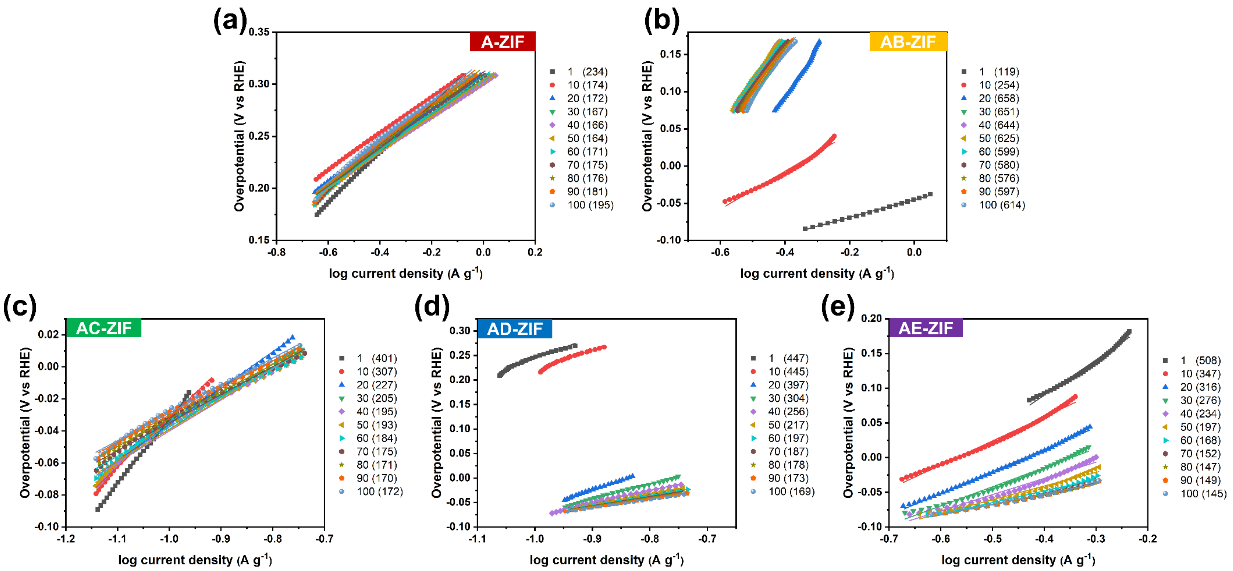

Fig. S10. Tafel plots of OER activity of A-ZIF (**a**), AB-ZIF (**b**), AC-ZIF (**c**), AD-ZIF (**d**) and AE-ZIF (**e**) between 0.85-1.55 V for different CV cycles (0, 10, 20, 30, 40, 50, 60, 70, 80, 90 and 100). Labeled as CV cycle number before parentheses, Tafel plots in parentheses (mV dec^{-1}).

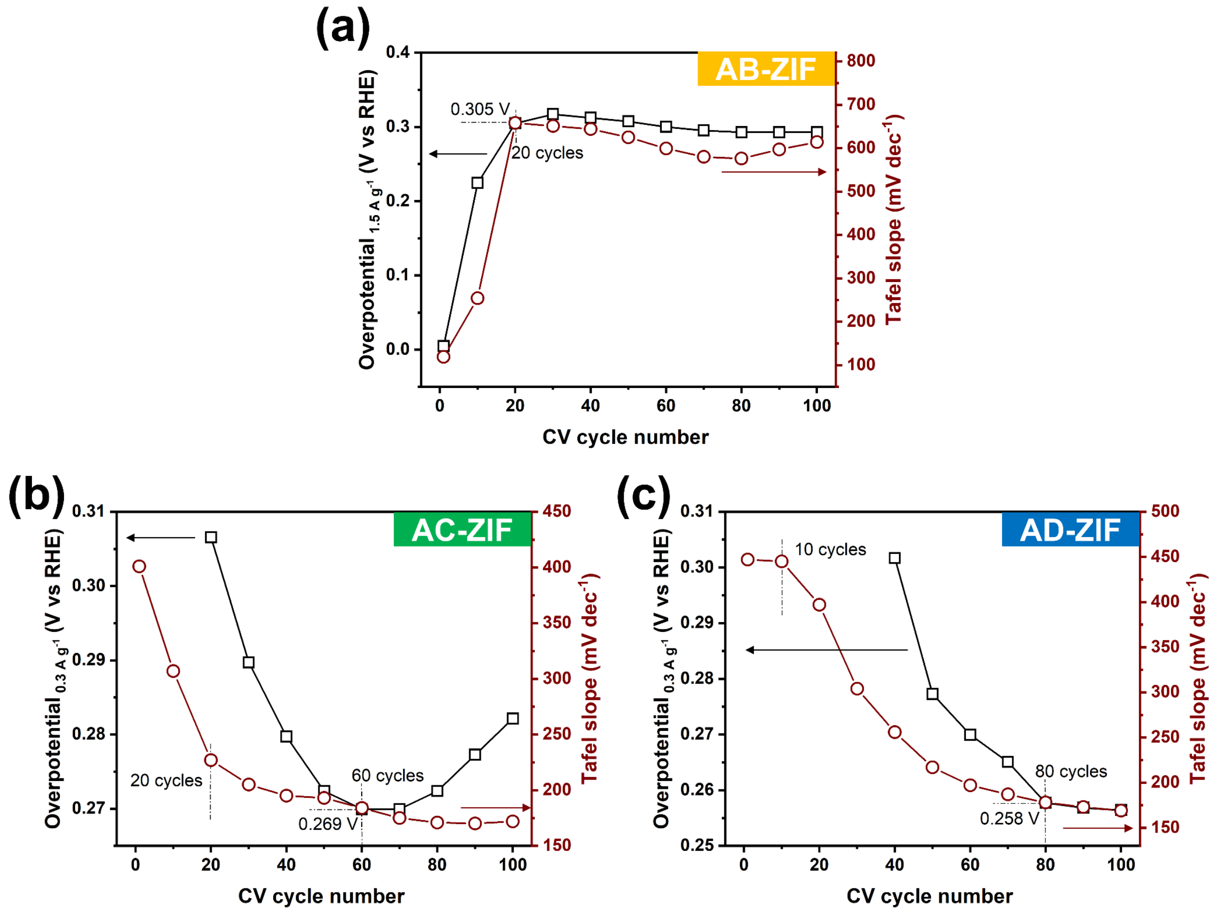

Fig. S11. Overpotential (current density is 1.5 A g^{-1} for AB-ZIF, 0.3 A g^{-1} for AC-ZIF and AD-ZIF) and Tafel slope plots with CV cycles of AB-ZIF (**a**), AC-ZIF (**b**) and AD-ZIF (**c**). The data for the Tafel slope for each CV are from Fig. S7, and calculations are from Fig. S10.

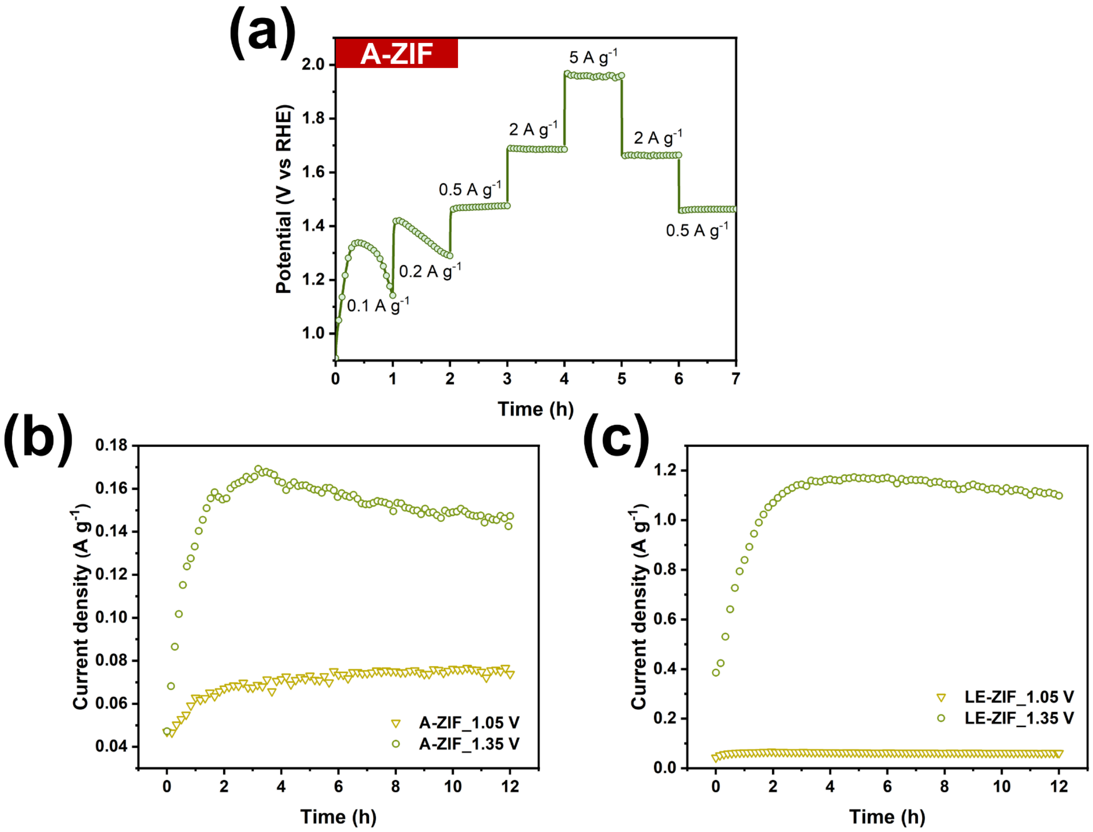

Fig. S12. **a**, Multistep potentiometric plot of A-ZIF at different current densities. **b**, **c**, Amperometric plots of A-ZIF (**b**) and AE-ZIF (**c**) at certain potentials of 1.05 and 1.35 V.

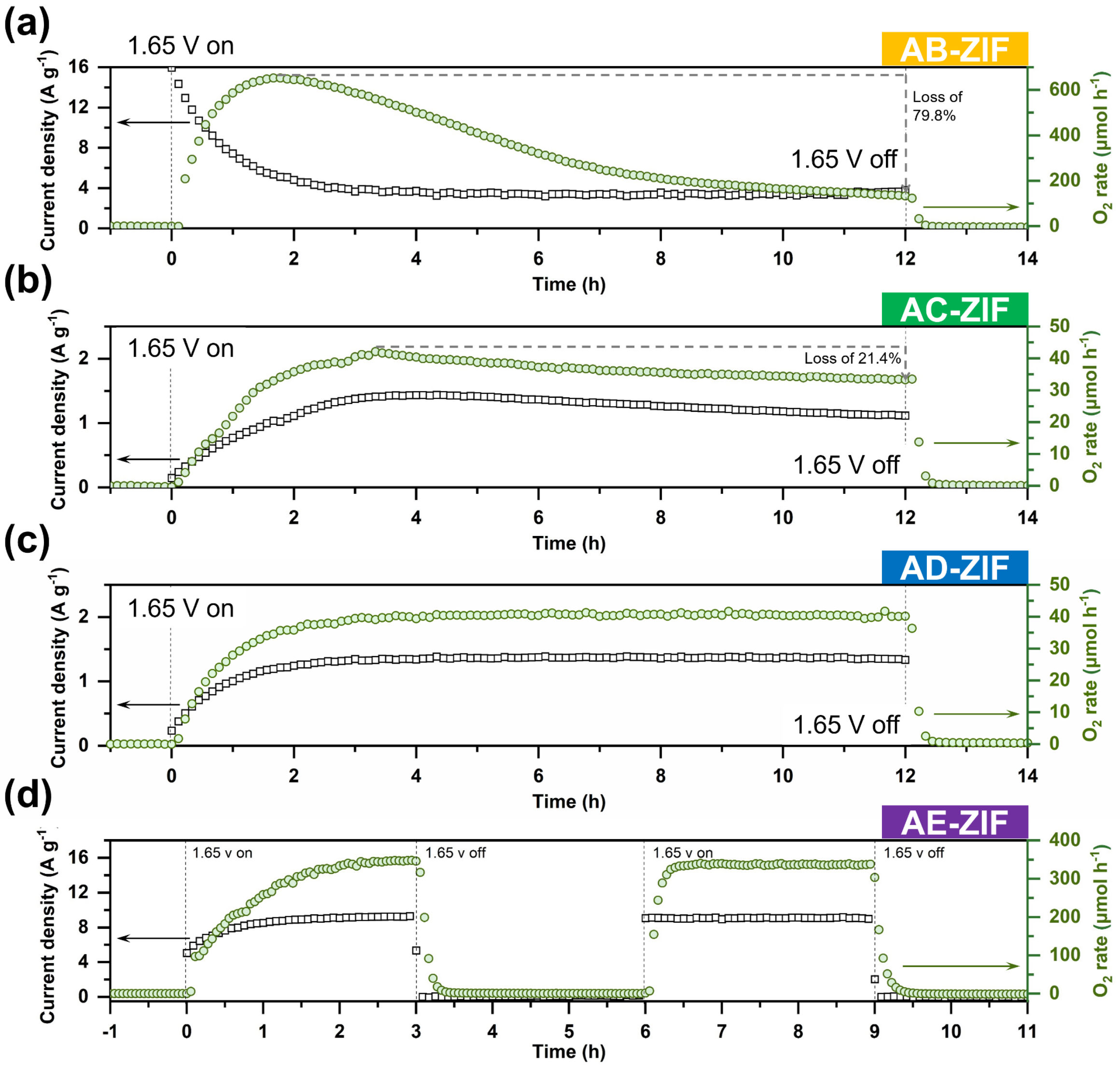

Fig. S13. a, **b**, **c**, Amperometric and O_2 evolution rate plots of AB-ZIF (**a**), AC-ZIF (**b**) and AD-ZIF (**c**) at certain potentials of 1.65 V. **d,** Amperometric and O_2 evolution rate plots of AE-ZIF under intermittent biased condition.

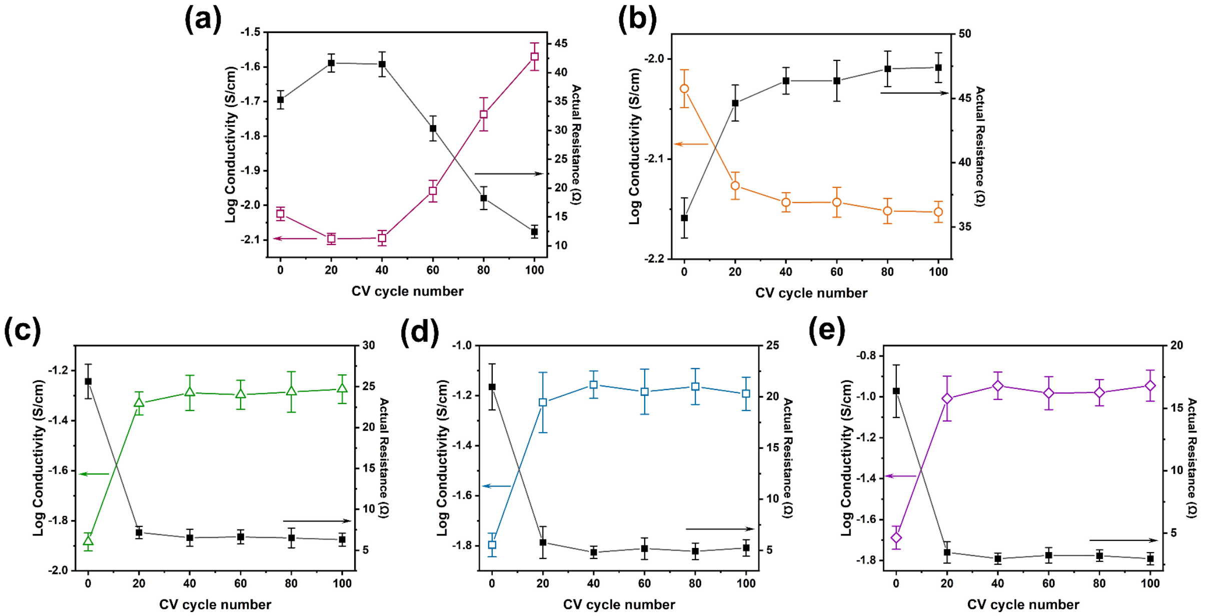

Fig. S14. Actual resistance value and electrical conductivity (log scale) of A-ZIF (**a**), AB-ZIF (**b**), AC-ZIF (**c**), AD-ZIF (**d**) and AE-ZIF (**e**) on FTO glass between 0.85-1.55 V with different CV cycles (0, 10, 20, 30, 40, 50, 60, 70, 80, 90 and 100).

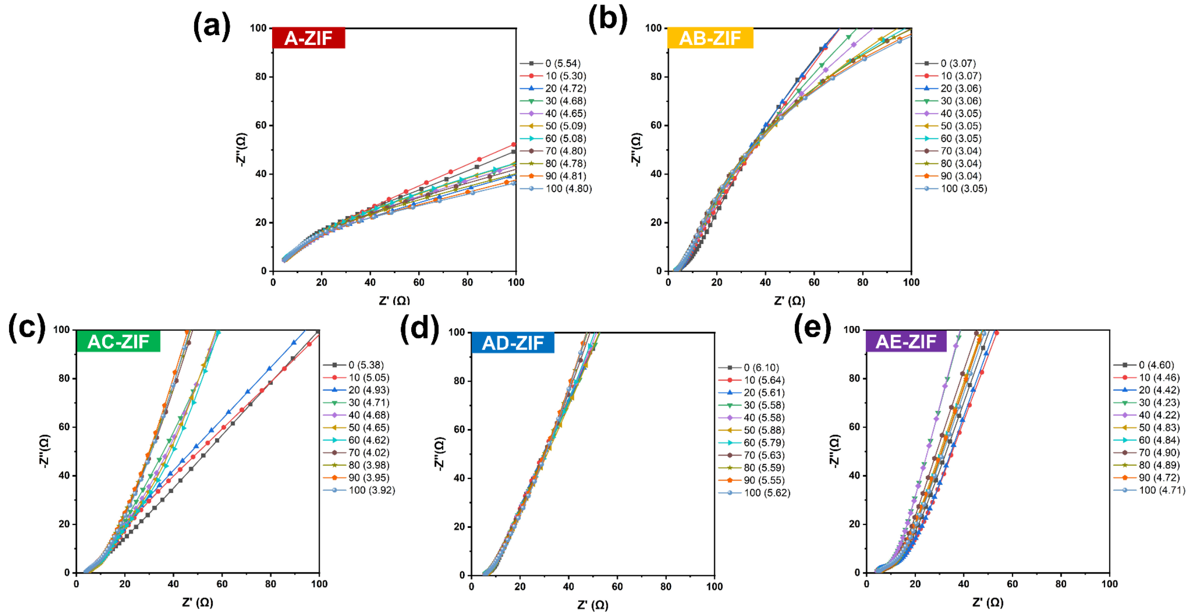

Fig. S15. Electrochemical impedance spectroscopy of A-ZIF (**a**), AB-ZIF (**b**), AC-ZIF (**c**), AD-ZIF (**d**) and AE-ZIF (**e**) on FTO glass between 0.85-1.55 V with different CV cycles (0, 10, 20, 30, 40, 50, 60, 70, 80, 90 and 100). Labeled as CV cycle number before parentheses, resistance value (R_{Ω}) at high frequency in parentheses.

S3. Structure evolution after electrocatalytic reaction

After 12-hour amperometry, except for an additional peak of $Co(OH)_2$ which appeared at 19.1°, the XRD patterns of AD and AE remain unchanged compared to before electrocatalysis (Fig. S16). This observation suggests that even under extended exposure to high potentials (1.65 V), AD and AE can preserve the inherent crystallinity of ZIF-67 well. The relatively low intensity of the additional $Co(OH)_2$ peak can be attributed to a cobalt (oxy)hydroxide layer constructed by surface reconstruction (Fig. 4). For other ZIF samples, the XRD patterns of A, AC and AB were consistent with the results after their respective CV tests.

In XPS analysis of AC and AD, no discernible signal indicative of Co^{3+} formation through electro-oxidation was detected during the high potential CV cycles. Moreover, two signals corresponding to Co-N (red) and C-N (blue) were retained in the N *1s* spectra [10], as depicted in Figs. S19 and S20. The N/Co ratio in AC and AD has still decreased slightly, from 6.63 and 5.51 (base) to 4.93 and 3.45 (high potential in Table S2). Conversely, the complete absence of the Co *2p* and N *1s* signals observed in AB-ZIF after electrocatalysis suggests the degradation of the framework structure (Fig. S18). This degradation is further evidenced by a significant decrease in the N/Co ratio, dropping from 3.83 (base) to 1.26 (high) under high potential window.

Quantitative analysis of ^1H NMR spectra before and after 100 CV cycles revealed the absence of the ligand NH_2-mIm in AB-ZIF, consistent with the results from XRD and XPS. The actual ratio of ligand bIm in AC-ZIF, increases from 41.4 mol% to 60.2 mol% (Table S3), indicating the detachment of the original ligands and the electro-oxidation of some Co species to higher valences.

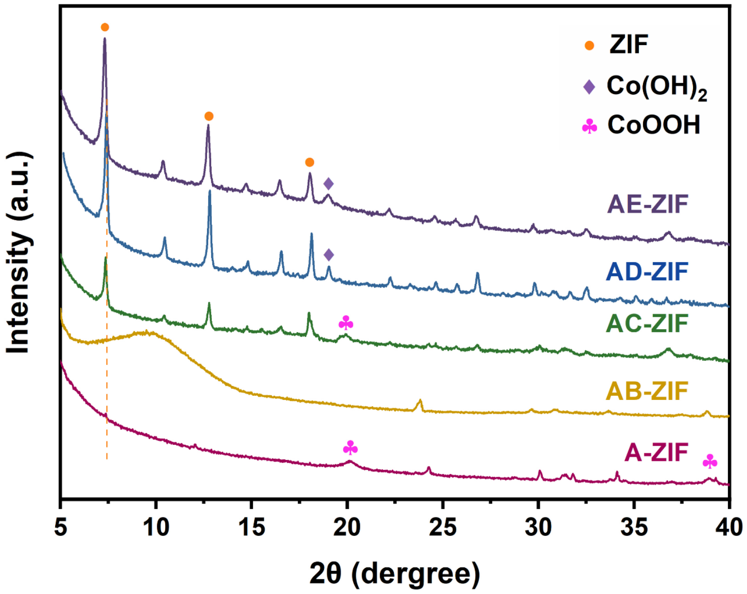

Fig. S16. XRD patterns of A-ZIF, AB-ZIF, AC-ZIF, AD-ZIF and AE-ZIF after 12-hour amperometry.

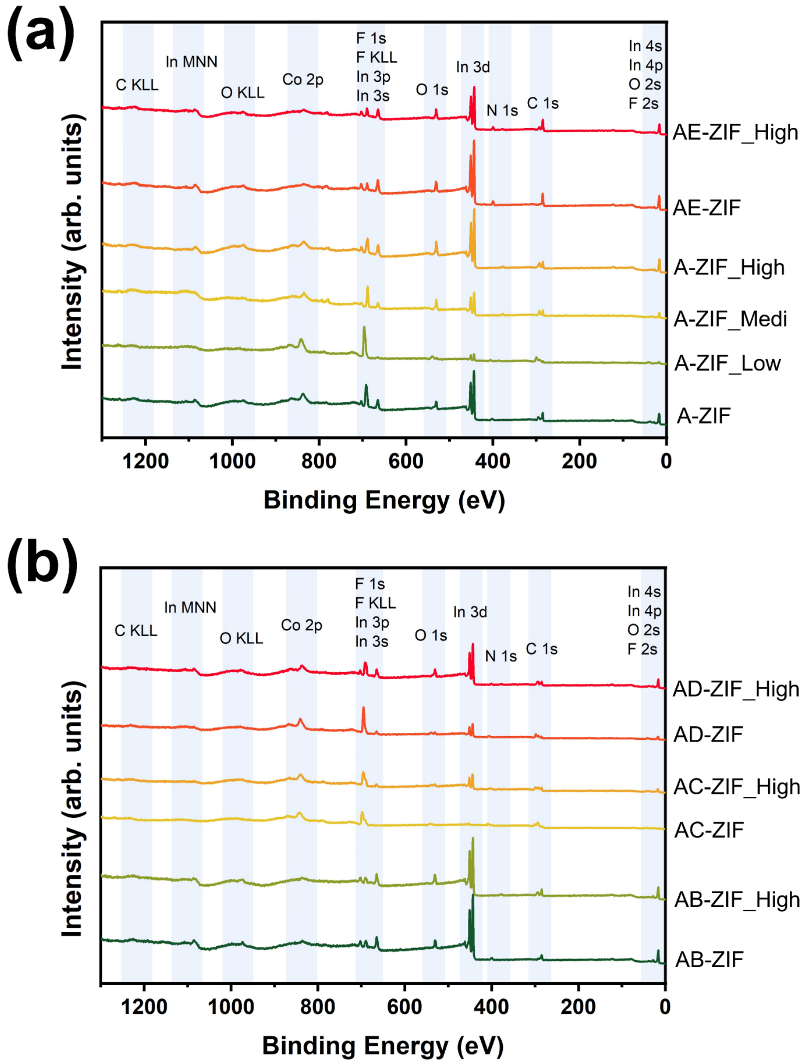

Fig. S17. XPS survey spectra of A-ZIF, AE-ZIF (**a**) and AB-ZIF, AC-ZIF, AD-ZIF (**b**) with 100 CV cycles at different potential window (Low: 0.85-1.05 V, Medi: 0.85-1.30 V, High: 0.85-1.55 V). Elemental Fluorine was obtained from the Nafion solution in the synthetic electrode.

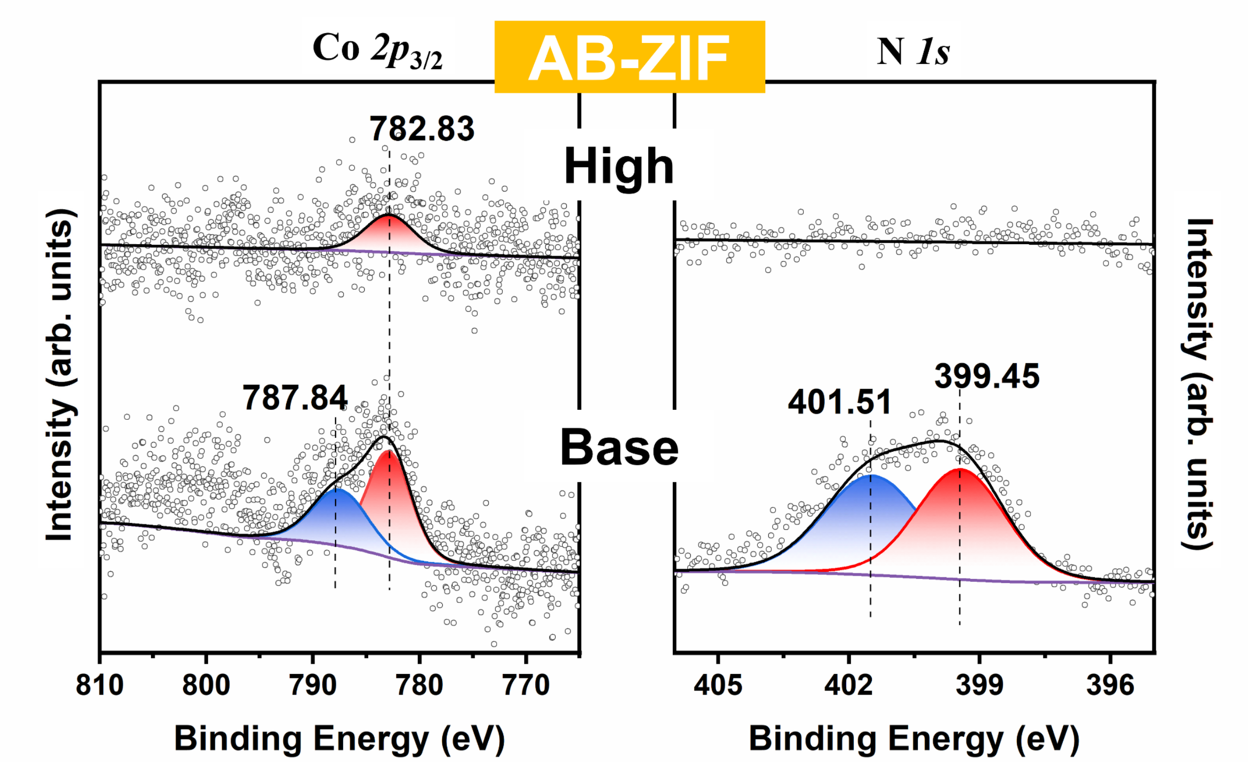

Fig. S18. Co *2p*$_{3/2}$ and N *1s* of XPS spectra of AB-ZIF with 100 CV cycles at high potential window (0.85-1.55 V).

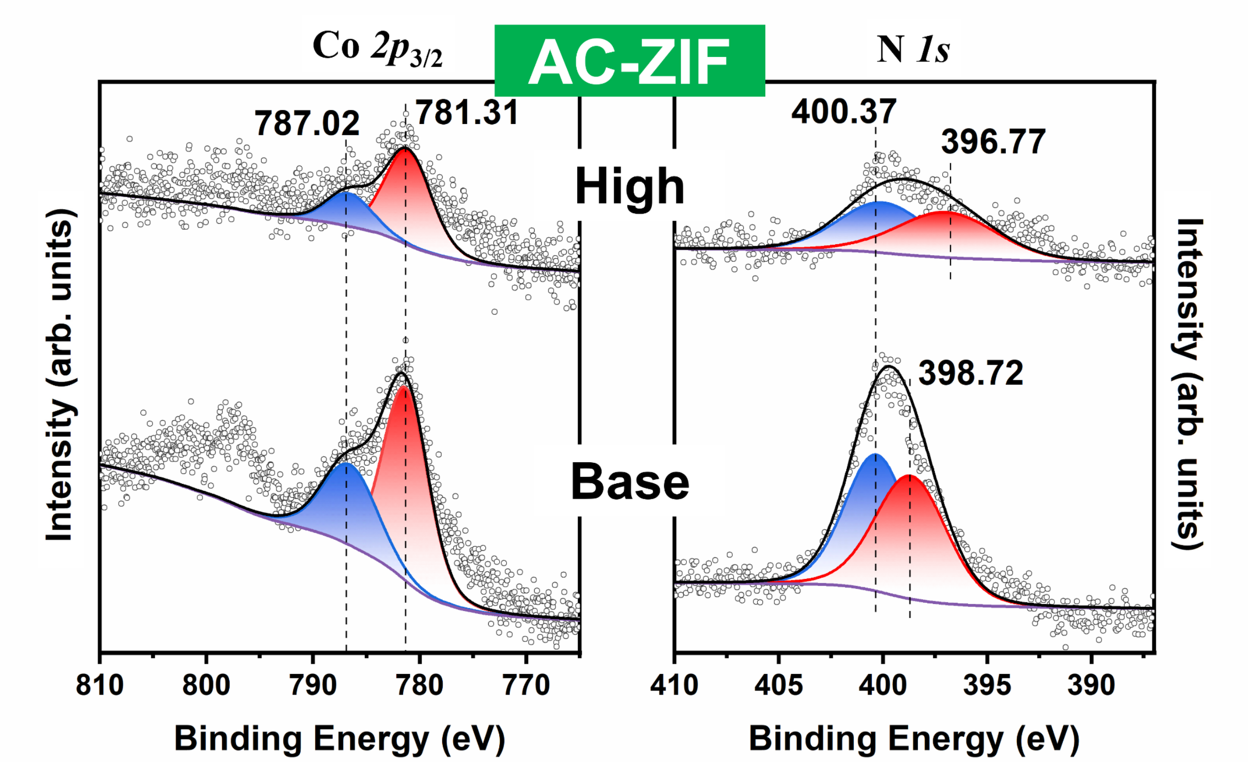

Fig. S19. Co *2p*$_{3/2}$ and N *1s* of XPS spectra of AC-ZIF with 100 CV cycles at high potential window (0.85-1.55 V).

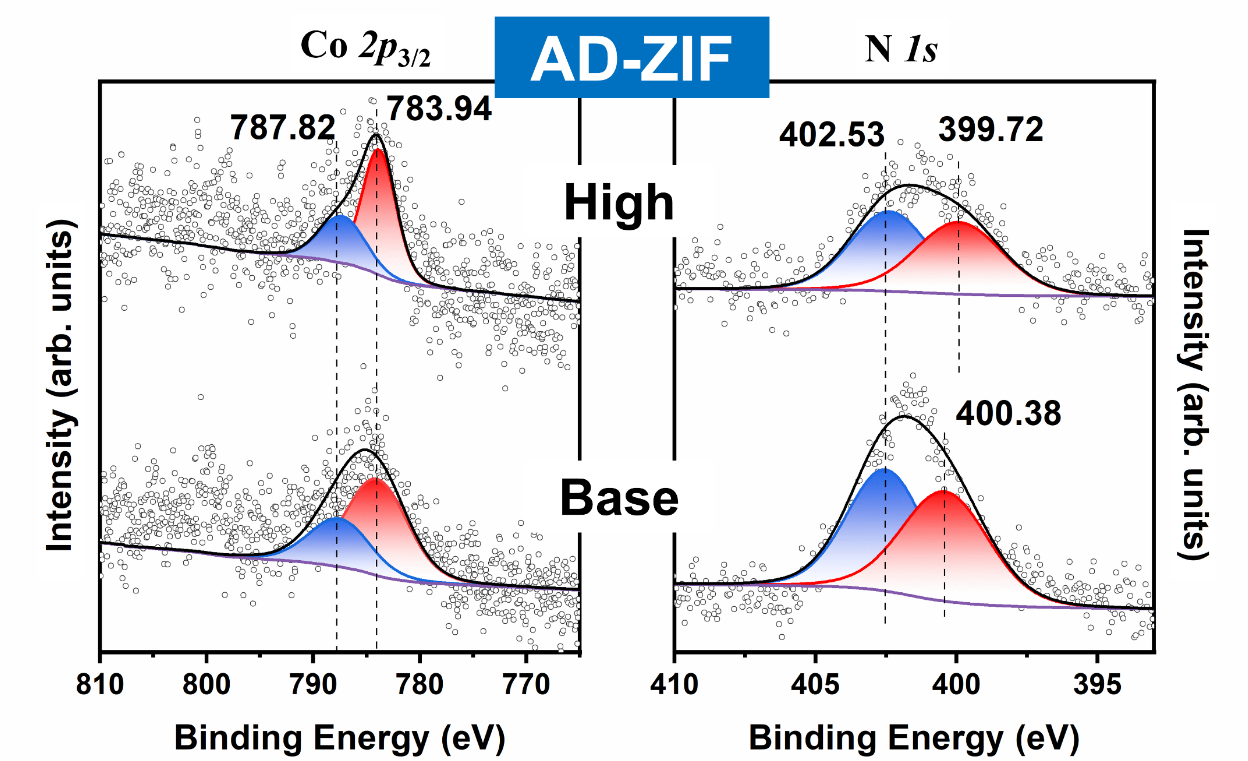

Fig. S20. Co *2p*$_{3/2}$ and N *1s* of XPS spectra of AD-ZIF with 100 CV cycles at high potential window (0.85-1.55 V).

Table S2. Cobalt, nitrogen and oxygen atomic ratios by XPS spectra.

Sample[a]	Co *2p*[b] (At.%)	N *1s* (At.%)	O *1s* (At.%)	N/Co	N/O
A-ZIF_Base	2.0	7.8	14.7	3.9	0.5
A-ZIF_Low	2.8	6.5	14.6	2.4	0.4
A-ZIF_Medi	3.3	0.0	27.6	0.0	0.0
A-ZIF_High	2.3	0.0	29.8	0.0	0.0
AB-ZIF_Base	2.8	10.9	26.2	3.8	0.4
AB-ZIF_High	0.6	0.7	28.2	1.3	0.0
AC-ZIF_Base	1.2	7.6	13.6	6.6	0.6
AC-ZIF_High	1.4	7.1	14.3	4.9	0.5
AD-ZIF_Base	1.4	7.8	16.2	5.5	0.5
AD-ZIF_High	1.6	5.5	23.0	3.5	0.2
AE-ZIF_Base	2.6	11.6	22.4	4.5	0.5
AE-ZIF_High	2.6	9.4	19.1	3.6	0.5

[a] Base means the sample before electrocatalysis. Low, medium, and high correspond to samples with 100 CV cycles at different potential window (Low: 0.85-1.05 V, Medi: 0.85-1.30 V, High: 0.85-1.55 V).

[b] All atomic ratios were determined by XPS spectroscopy in Fig. S17.

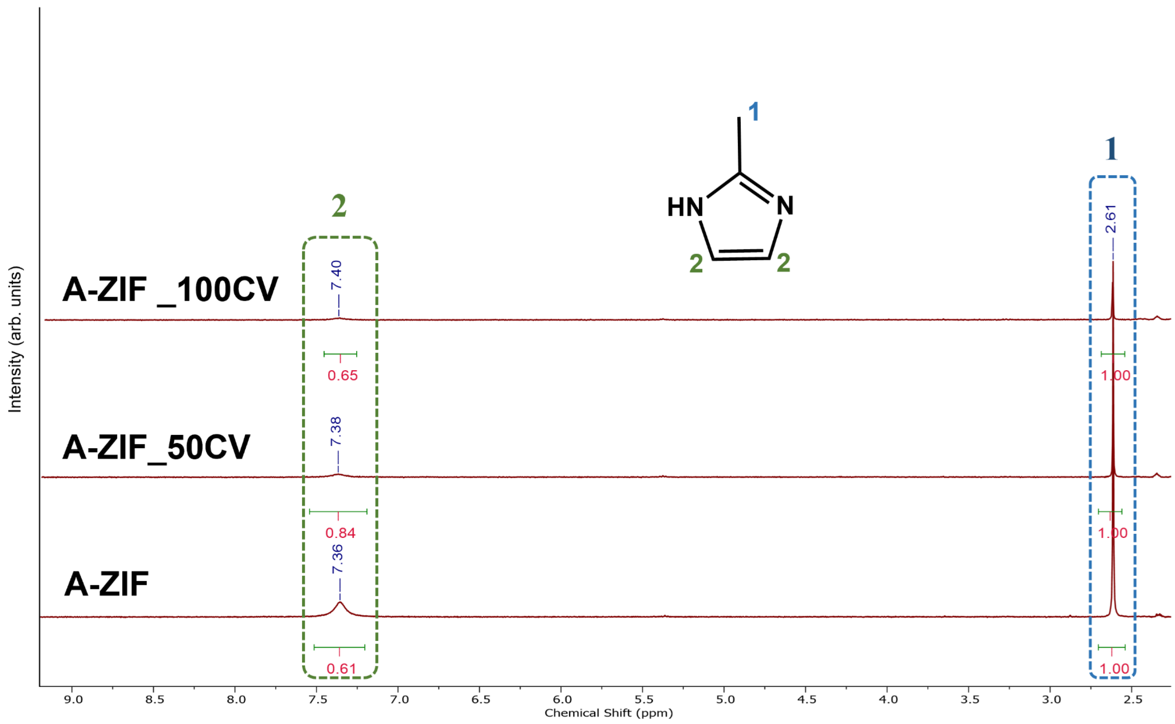

Fig. S21. ^{1}H NMR of A-ZIF, A-ZIF after 50 CV and A-ZIF after 100 CV cycles at high potential window (0.85-1.55 V).

A-ZIF: *^{1}H NMR (250 MHz, Acetic Acid-d_4) δ 7.36 (s, 1H), 2.61 (s, 1H).* A-ZIF after 50 CV: *^{1}H NMR (250 MHz, Acetic Acid-d_4) δ 7.38 (s, 1H), 2.63 (s, 1H).* A-ZIF after 100 CV: *^{1}H NMR (250 MHz, Acetic Acid-d_4) δ 7.40 (s, 1H), 2.65 (s, 1H).*

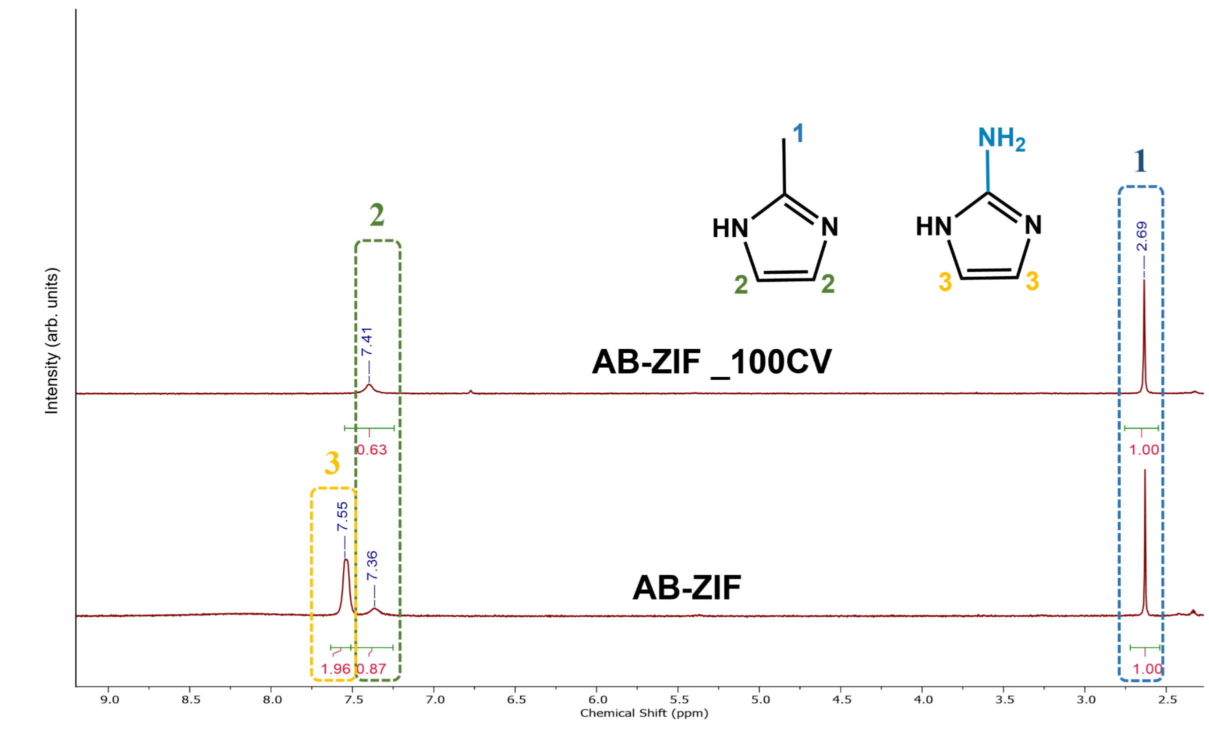

Fig. S22. ^{1}H NMR of AB-ZIF and AB-ZIF after 100 CV cycles at high potential window (0.85-1.55 V).

AB-ZIF: *^{1}H NMR (250 MHz, Acetic Acid-d_4) δ 7.55 (s, 2H), 7.36 (s, 1H), 2.63 (s, 1H).* AB-ZIF after 100 CV: *^{1}H NMR (250 MHz, Acetic Acid-d_4) δ 7.41 (s, 1H), 2.69 (s, 1H).*

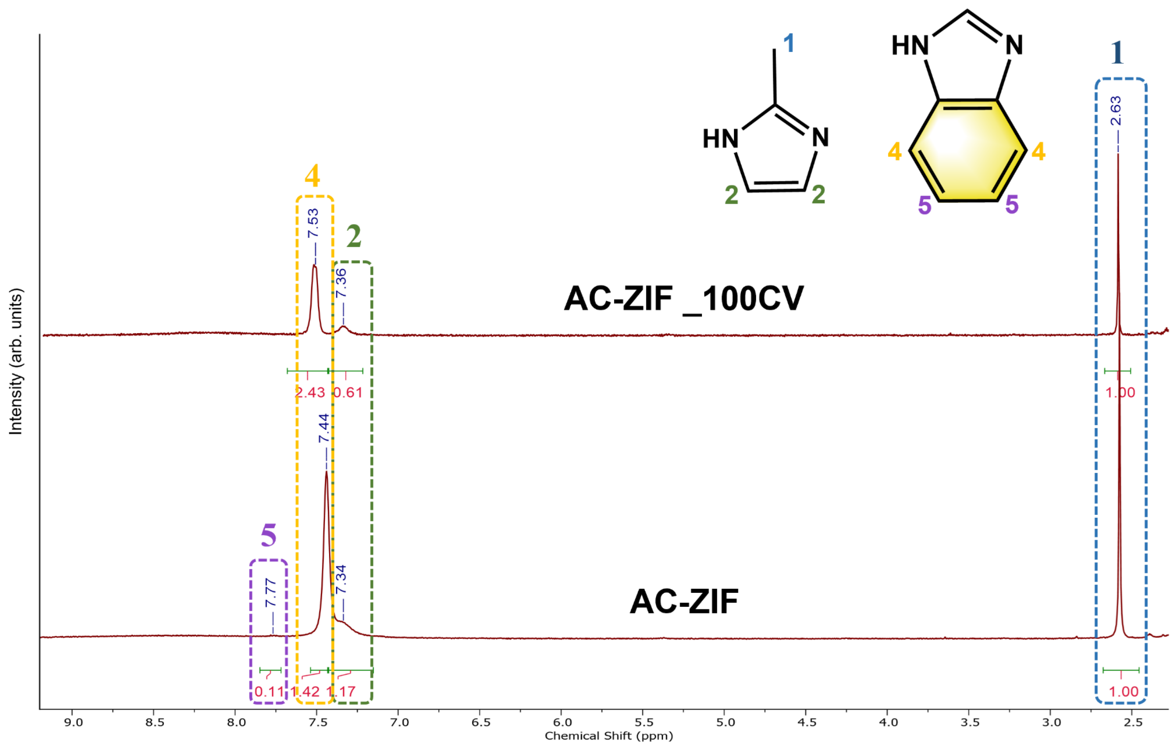

Fig. S23. ^{1}H NMR of AC-ZIF and AC-ZIF after 100 CV cycles at high potential window (0.85-1.55 V).

AC-ZIF: *^{1}H NMR (250 MHz, Acetic Acid-d_4) δ 7.44 (s, 1H), 7.34 (s, 1H), 2.58 (s, 1H).* AC-ZIF after 100 CV: *^{1}H NMR (250 MHz, Acetic Acid-d_4) δ 7.53 (s, 2H), 7.36 (s, 1H), 2.63 (s, 1H).*

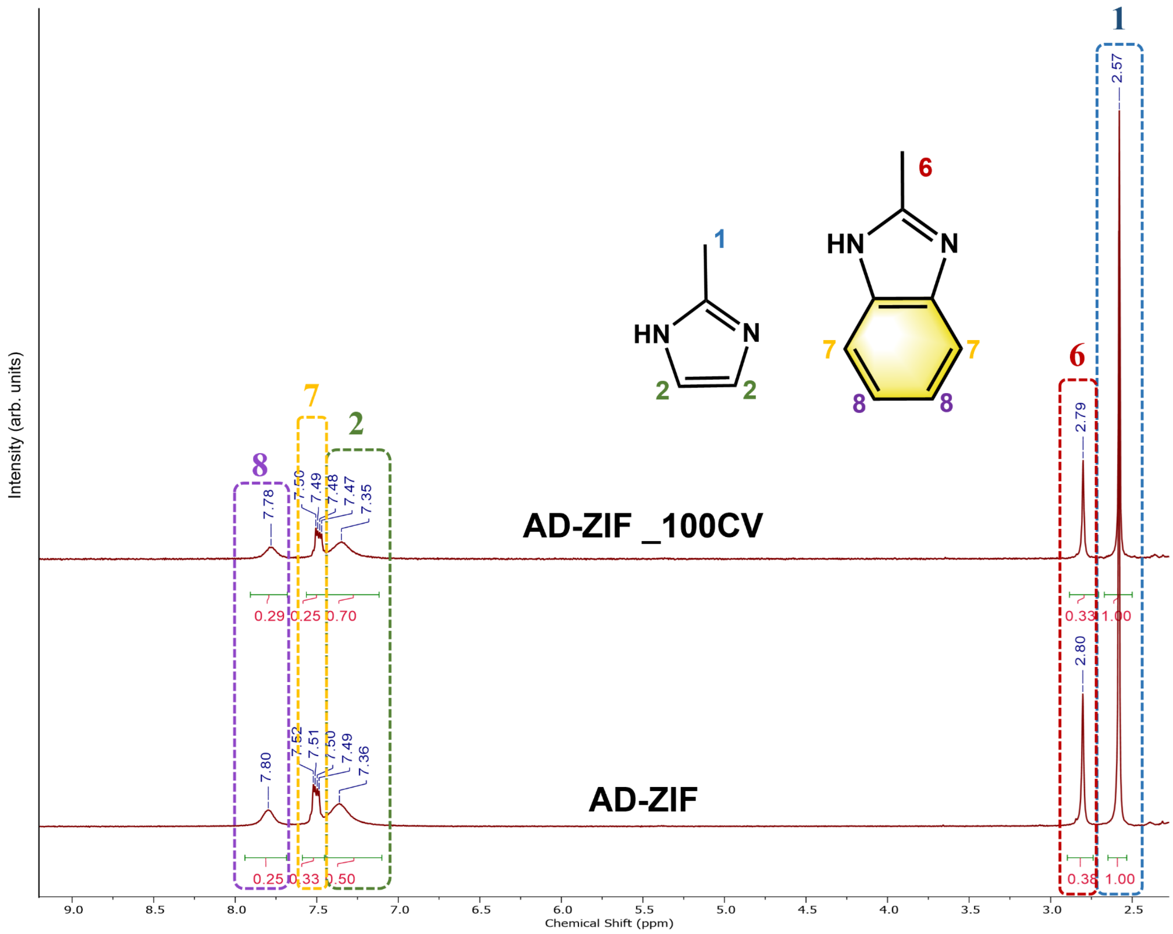

Fig. S24. ^{1}H NMR of AD-ZIF and AD-ZIF after 100 CV cycles at high potential window (0.85-1.55 V).

AD-ZIF: *^{1}H NMR (250 MHz, Acetic Acid-d_4) δ 7.80 (s, 0H), 7.50 (dd, J = 6.1, 2.9 Hz, 0H), 7.36 (s, 1H), 2.80 (s, 0H), 2.58 (s, 1H).*

AC-ZIF after 100 CV: *^{1}H NMR (250 MHz, Acetic Acid-d_4) δ 7.78 (s, 0H), 7.49 (dd, J = 6.1, 2.9 Hz, 0H), 7.35 (s, 1H), 2.79 (s, 0H), 2.57 (s, 1H).*

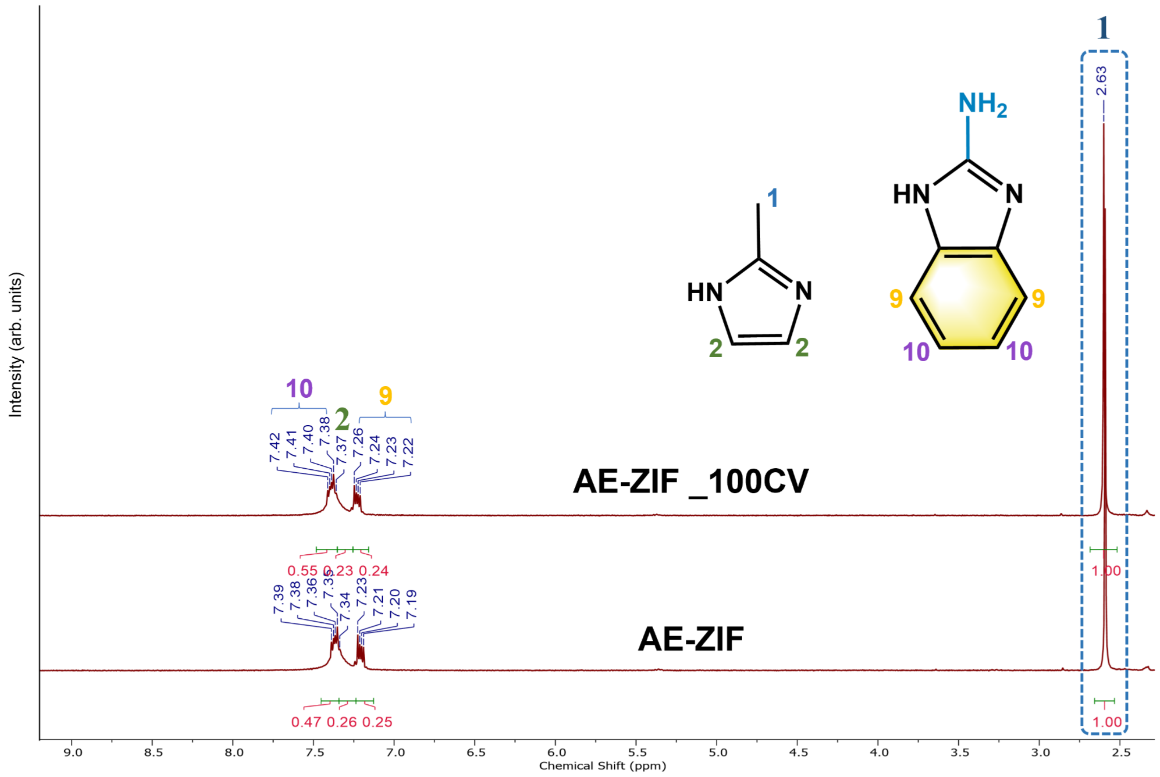

Fig. S25. ^1H NMR of AE-ZIF and AE-ZIF after 100 CV cycles at high potential window (0.85-1.55 V).

AE-ZIF: *^1H NMR (250 MHz, Acetic Acid-d_4) δ 7.37 (dd, J = 5.9, 3.2 Hz, 0H), 7.34 (s, 0H), 7.21 (dd, J = 5.9, 3.2 Hz, 0H), 2.59 (s, 1H)*. AE-ZIF after 100 CV: *^1H NMR (250 MHz, Acetic Acid-d_4) δ 7.40 (dd, J = 5.9, 3.2 Hz, 0H), 7.37 (s, 0H), 7.24 (dd, J = 5.9, 3.2 Hz, 0H), 2.63 (s, 1H).*

Table S3. Calculation of the ligand ratio in LE-ZIFs by ^{1}H NMR.

**Samples**	**AB-ZIF**	**AC-ZIF**	**AD-ZIF**	**AE-ZIF**
**Synthetic mass of ligand B-E/A (g/g)**	1/1	1/1	1/1	1/1
**Synthetic ratio of ligand B-E (mol%)**	49.6	41.0	38.3	38.1
**Actual ratio of ligand B-E (mol%)**	51.2	41.4	39.0	36.9
**Actual ratio of ligand B-E after 100 CV (mol%)**	-	60.2	33.9	39.1

All ^{1}H NMR data were quantified from Figs. S21-25.

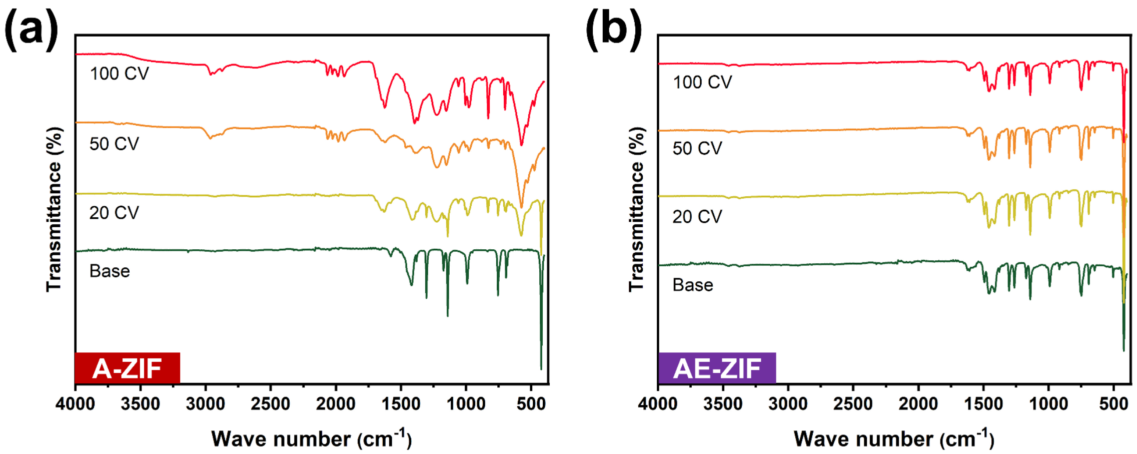

Fig. S26. ATR-IR spectra of A-ZIF (**a**) and AE-ZIF (**b**) between 0.85-1.55 V with different CV cycles (0, 20, 50 and 100).

S4. Evolution of morphology and elemental distribution after electrocatalytic reaction

Both A and AE exhibit distinct dodecahedral crystal grains with clear and sharp edges before electrocatalytic reaction, indicating their well-defined morphology (Figs. 4 and S27). Elemental mapping via EDS reveals the uniform distribution of elements within ZIF particles, indicating the homogeneous distribution of the secondary ligand without aggregation together, contrasting markedly with the structured evolution post electrocatalysis.

After 12-hour amperometry, enlargement of the AE-ZIF particle edge in Fig. S30 reveals the lattice fringes of 0.23, 0.24 and 0.27 nm within surface layer, corresponding well with the (012) plane of CoOOH and (101) and (100) planes of $Co(OH)_2$, as indicated by bright rings in the SAED pattern [11, 12]. Other LE samples, AC and AD exhibit the in-situ formation of a cobalt (oxy)hydroxide layer similar to AE-ZIF on their surfaces (Figs. S32 and S33). The apparent aggregation of Co and O elements on the particle surfaces in the EDS mapping further confirms the presence of the outer layer structure. Interestingly, although AB-ZIF transitions to an amorphous during electrocatalysis according to XRD pattern, some agglomerates of $Co(OH)_2$ and CoOOH nanoparticles are still observed in TEM images. The distribution of Co and O elements in EDS mapping, lattice fringes, and bright spots in the corresponding selected area FFT patterns further confirm the crystalline characteristics of these agglomerated particles (Fig. S31).

The atomic ratio of oxygen elements increases significantly to 49.21% and 23.23% after 12-hour amperometry for A and AB, contrasting with the nearly negligible oxygen content before the reaction (Figs. S28e and S31f). This further supports the complete reconstruction of A and AB, transitioning into high-valence cobalt (oxy)hydroxides. In contrast, the atomic ratio of oxygen elements in AC, AD and AE remains clearly controlled within the range of 1 to 3.12%, indicating incomplete reconstruction with only slight electro-oxidation occurring on the particle surface.

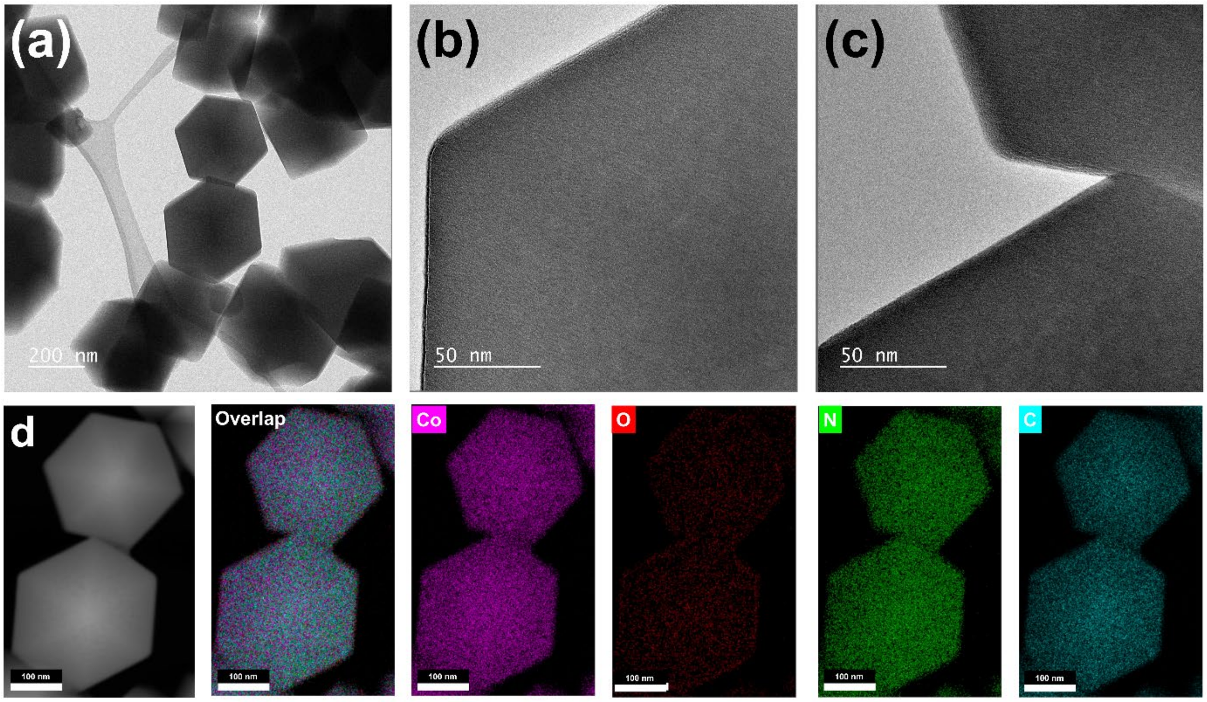

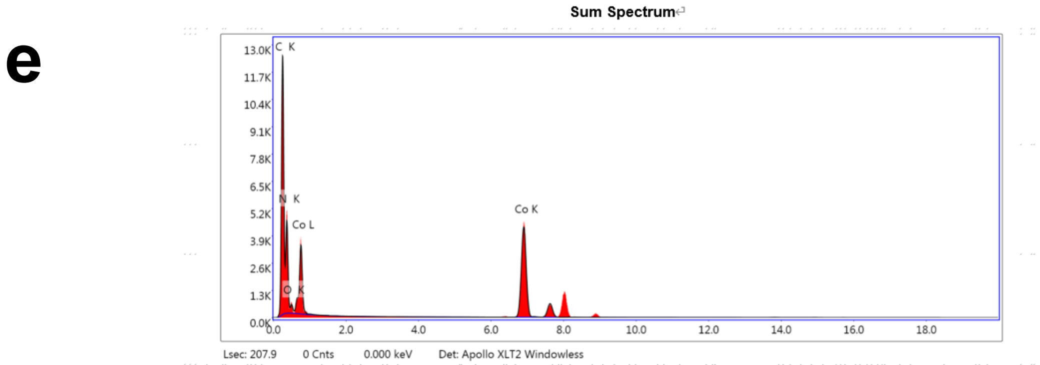

MThin Smart Quant Results (Theoretical)

Element	Weight %	Atomic %	Net Int.	Net Error%	KABFactor
C K	39.08	58.16	909.90	0.28	1
N K	23.04	29.40	328.60	0.77	1.63
O K	1.17	1.31	23.00	8.81	1.18
CoK	36.71	11.13	682.20	0.39	1.25

Fig. S27. a, TEM image of A-ZIF. **b**, **c**, Enlarged TEM images of A-ZIF. **d**, Elemental mapping of A-ZIF showing the Co, O, N and C distribution. **e**, EDS quantitative elemental analysis of A-ZIF.

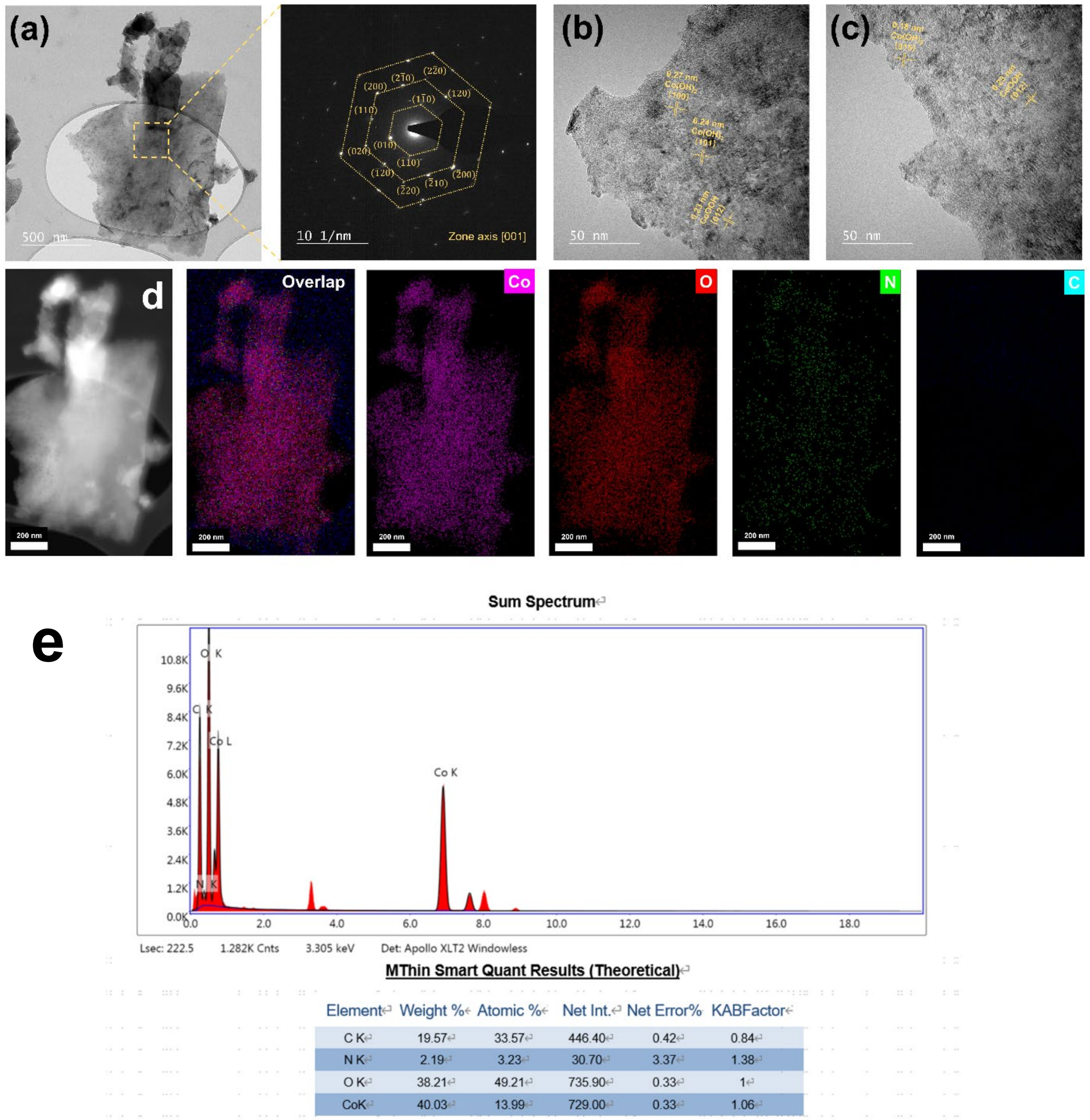

Element	Weight %	Atomic %	Net Int.	Net Error%	KABFactor
C K	19.57	33.57	446.40	0.42	0.84
N K	2.19	3.23	30.70	3.37	1.38
O K	38.21	49.21	735.90	0.33	1
CoK	40.03	13.99	729.00	0.33	1.06

Fig. S28. a, TEM image of A-ZIF after 12-hour amperometry, with the corresponding SAED pattern. **b**, **c**, Enlarged TEM images of A-ZIF after 12-hour amperometry. **d**, Elemental mapping of A-ZIF after 12-hour amperometry showing the Co, O, N and C distribution. **e**, EDS quantitative elemental analysis of A-ZIF after 12-hour amperometry.

a

Sum Spectrum

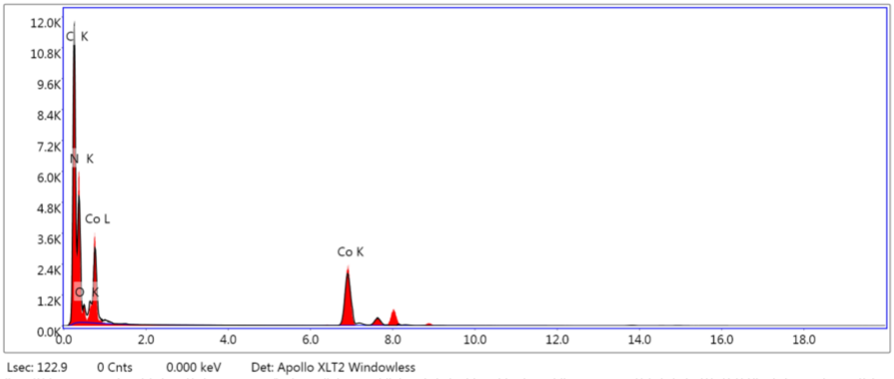

MThin Smart Quant Results (Theoretical)

Element	Weight %	Atomic %	Net Int.	Net Error%	KABFactor
C K	46.32	59.59	1722.40	0.25	1
N K	30.91	34.10	703.90	1.22	1.63
O K	0.48	0.46	15.00	72.23	1.18
CoK	22.29	5.84	661.50	0.76	1.25

b

Sum Spectrum

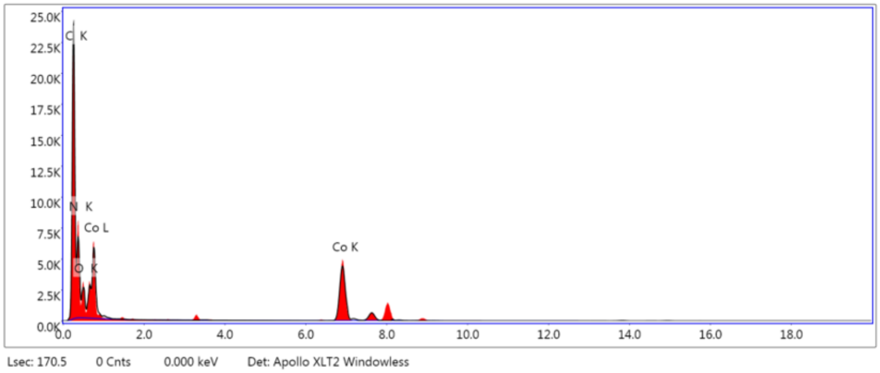

MThin Smart Quant Results (Theoretical)

Element	Weight %	Atomic %	Net Int.	Net Error%	KABFactor
C K	50.52	66.58	2459.70	0.21	1
N K	20.72	23.42	617.90	1.75	1.63
O K	3.16	3.12	129.80	10.54	1.18
CoK	25.60	6.88	995.00	0.54	1.25

Fig. S29. EDS quantitative elemental analysis of AE-ZIF before (**a**) and after 12-hour amperometry (**b**).

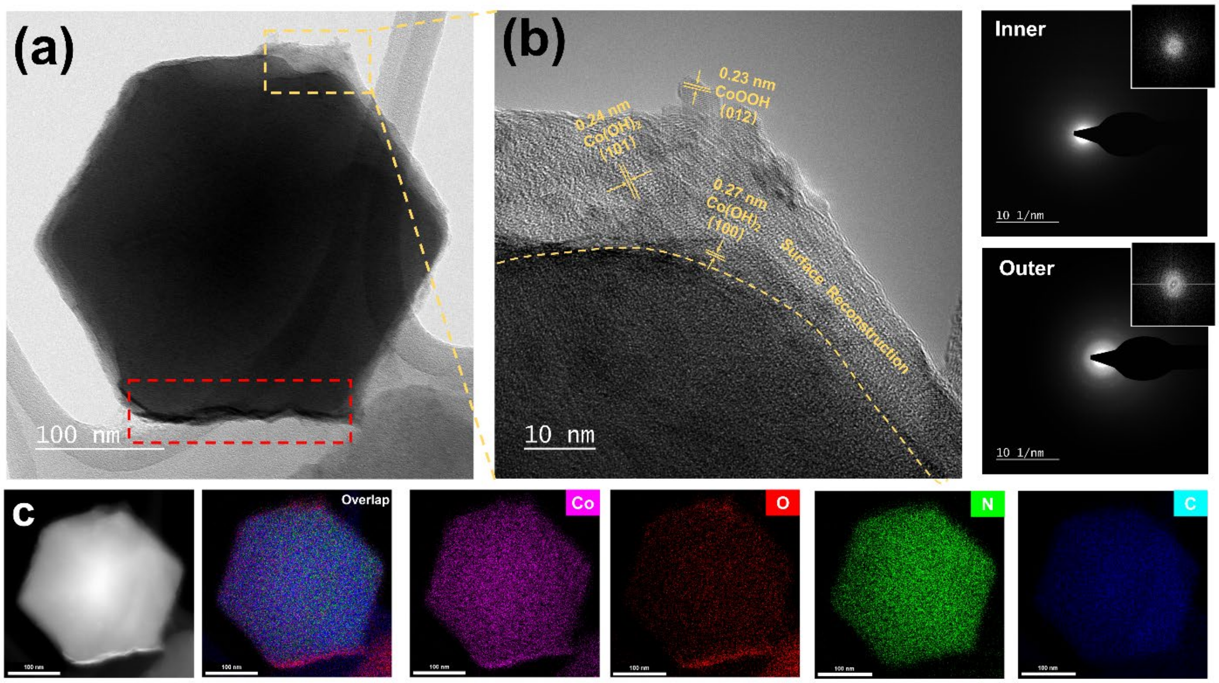

d

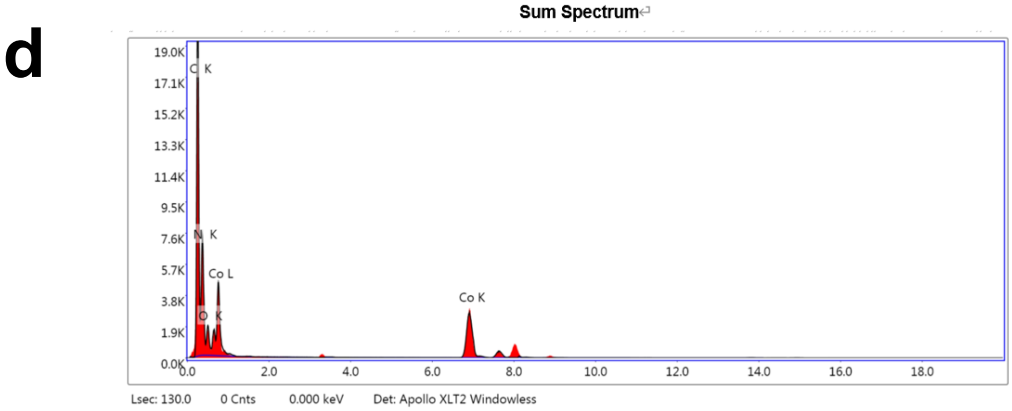

MThin Smart Quant Results (Theoretical)

Element	Weight %	Atomic %	Net Int.	Net Error%	KABFactor
C K	51.74	65.61	2436.20	0.32	1
N K	24.59	26.74	709.20	1.62	1.63
O K	2.21	2.11	88.00	15.99	1.18
CoK	21.46	5.55	806.60	0.71	1.25

Fig. S30. a, TEM image of single-particle AE-ZIF after 12-hour amperometry. **b**, Enlarged TEM image of AE-ZIF edge after 12-hour amperometry, the corresponding SAED pattern. **c**, Elemental mapping of single-particle AE-ZIF after 12-hour amperometry showing the Co, O, N and C distribution. **d**, EDS quantitative elemental analysis of AE-ZIF after 12-hour amperometry.

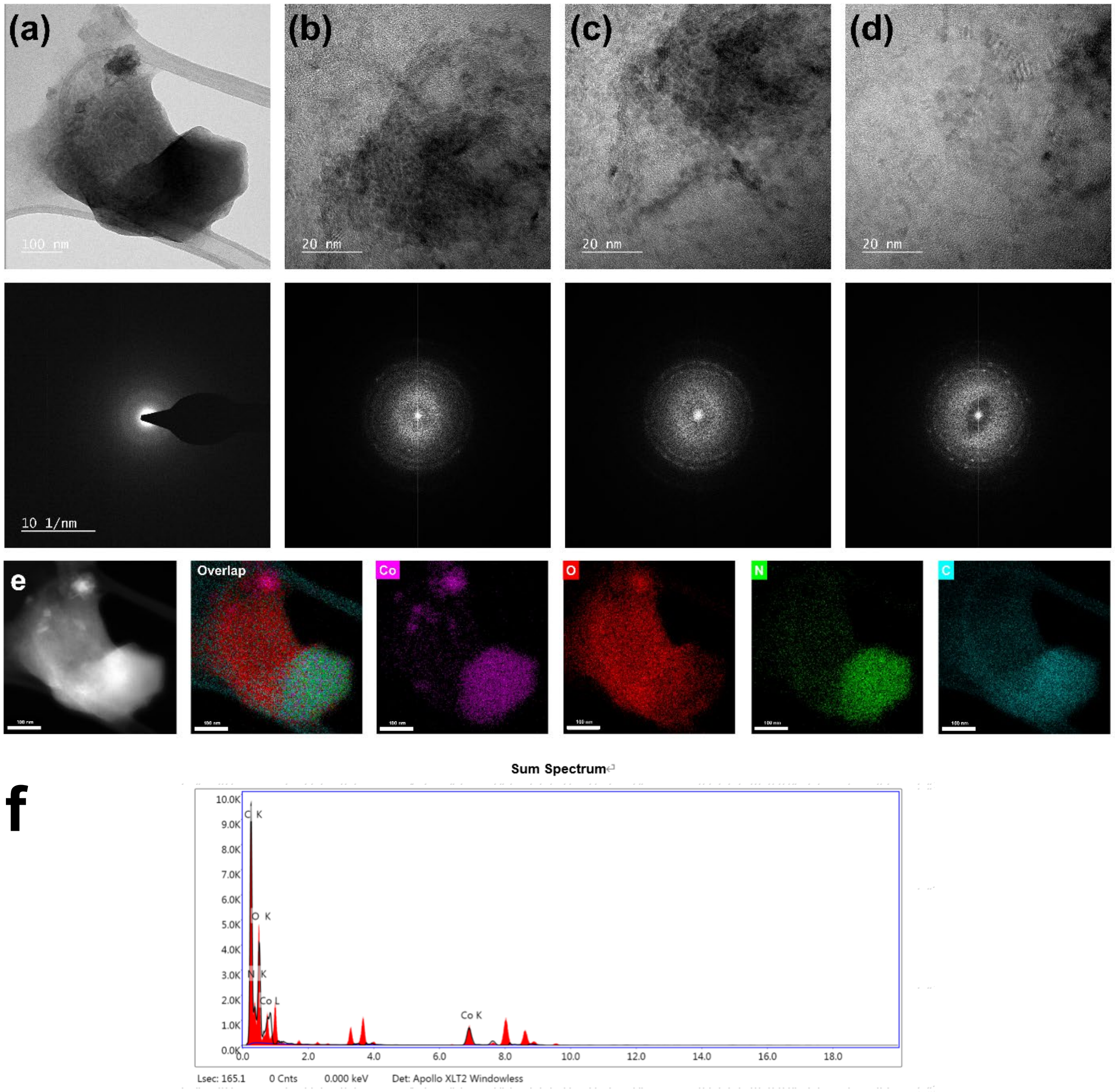

MThin Smart Quant Results (Theoretical)

Element	Weight %	Atomic %	Net Int.	Net Error%	KABFactor
C K	54.02	64.36	1050.20	0.42	1
N K	9.70	9.91	115.50	3.76	1.63
O K	25.97	23.23	426.30	1.57	1.18
CoK	10.31	2.50	159.90	1.22	1.25

Fig. S31. **a**, TEM image of AB-ZIF after 12-hour amperometry, with the corresponding SAED and FFT patterns. **b**, **c**, **d**, Enlarged TEM images of AB-ZIF after 12-hour amperometry. **e**, Elemental mapping of AB-ZIF after 12-hour amperometry showing the Co, O, N and C distribution. **f**, EDS quantitative elemental analysis of AB-ZIF after 12-hour amperometry.

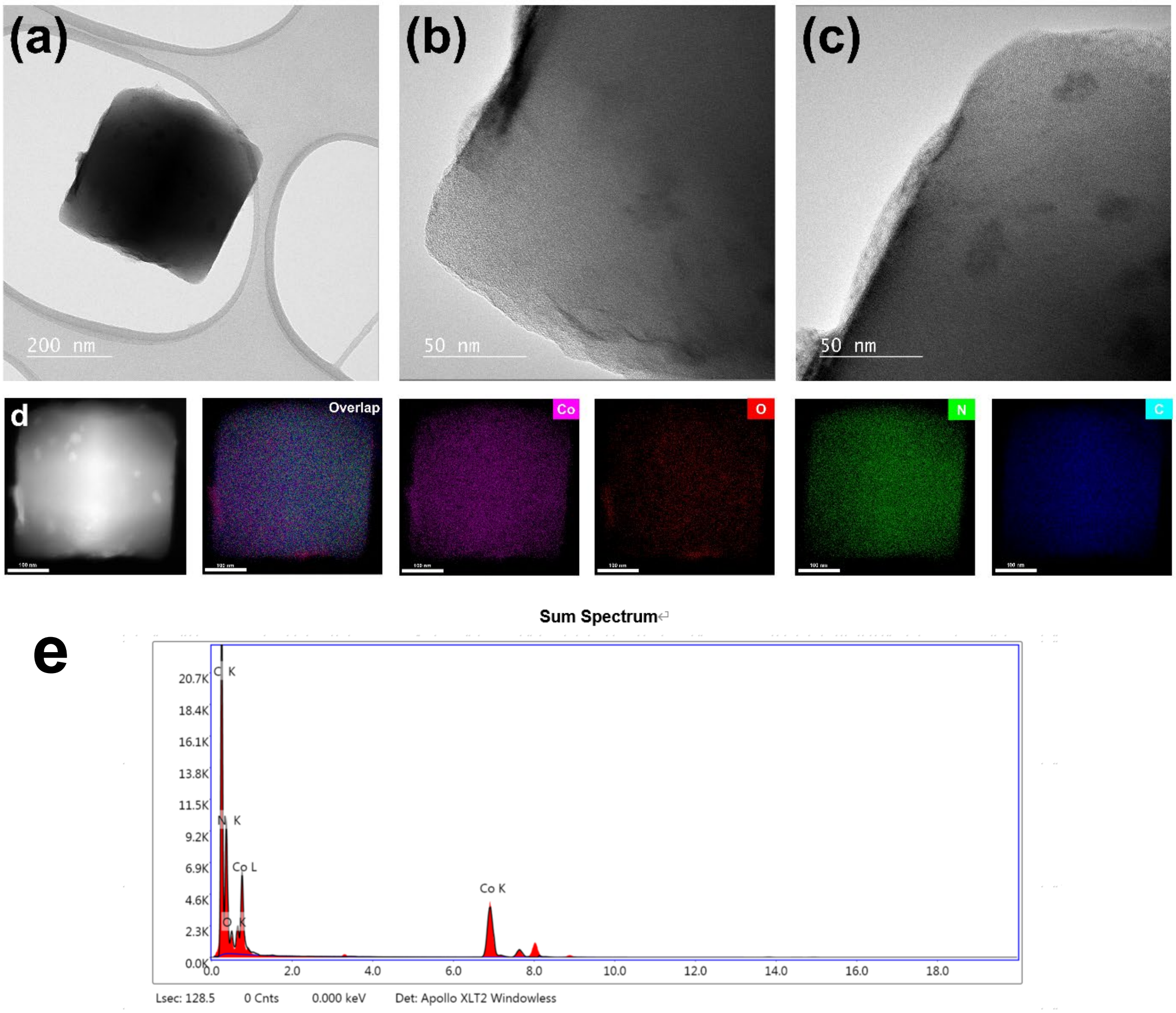

MThin Smart Quant Results (Theoretical)

Element	Weight %	Atomic %	Net Int.	Net Error%	KABFactor
C K	48.31	62.52	2873.00	0.27	1
N K	26.96	29.92	981.90	1.30	1.63
O K	1.47	1.43	73.70	21.77	1.18
CoK	23.27	6.14	1104.80	0.66	1.25

Fig. S32. **a**, TEM image of AC-ZIF after 12-hour amperometry. **b**, **c**, Enlarged TEM images of AC-ZIF after 12-hour amperometry. **d**, Elemental mapping of AC-ZIF after 12-hour amperometry showing the Co, O, N and C distribution. **e**, EDS quantitative elemental analysis of AC-ZIF after 12-hour amperometry.

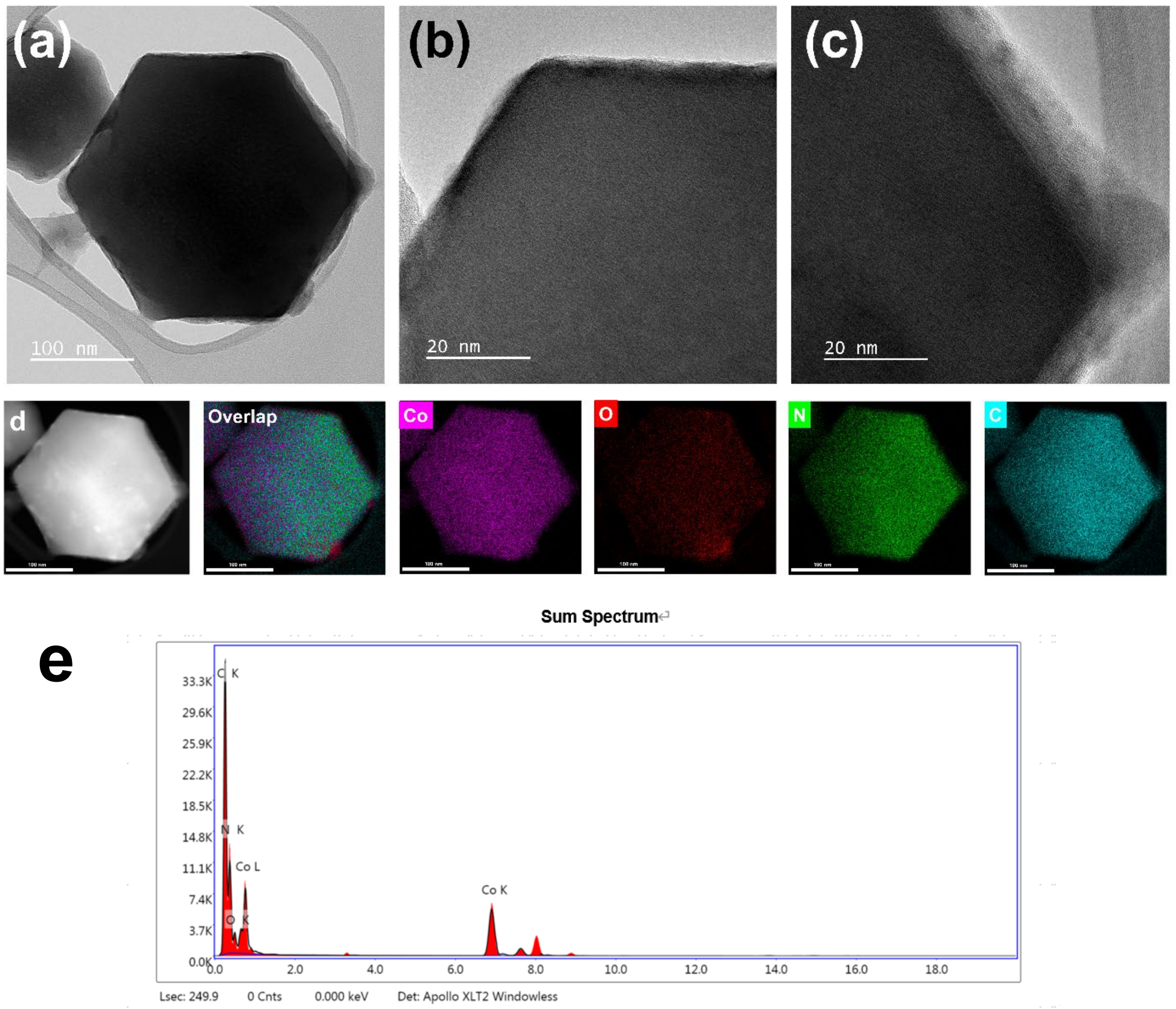

MThin Smart Quant Results (Theoretical)

Element	Weight %	Atomic %	Net Int.	Net Error%	KABFactor
C K	51.79	66.18	2474.00	0.17	1
N K	24.53	26.88	717.80	1.34	1.63
O K	1.10	1.06	44.40	28.30	1.18
CoK	22.58	5.88	861.10	0.52	1.25

Fig. S33. **a**, TEM image of AD-ZIF after 12-hour amperometry. **b**, **c**, Enlarged TEM images of AD-ZIF after 12-hour amperometry. **d**, Elemental mapping of AD-ZIF after 12-hour amperometry showing the Co, O, N and C distribution. **e**, EDS quantitative elemental analysis of AD-ZIF after 12-hour amperometry.

S5. In-situ UV–Vis absorption and Raman spectroscopy during electrocatalytic reactions

During the in-situ electrochemical UV–Vis absorption measurements, AC, AD and AE also exhibit the broad feature, associated to the same transition of tetrahedral Co species. Sample AB-ZIF only shows absorption signals at 328 and 354 nm, corresponding to ligand 2-mIm and NH_2-mIm, respectively (Fig. S35a). The broad signals associated with tetrahedral Co species in AC and AD noticeably weaken after 50 and 60 CV cycles, in addition to AC-ZIF displaying a CoOOH signal at 369 nm (Figs. S35b and S35c) [13]. The most unstable one is AB-ZIF, where the ligand signals disappear after 10 CV cycles, indicating that the intrinsic framework had been degraded.

For the other LE-ZIFs analyzed by in-situ electrochemical Raman spectroscopy, the secondary ligands bIm and 2-bIm are attributed to characteristic peaks at 701, 775 and 685 cm^{-1} in AC and AD (Figs. S36 and S37) [14, 15]. Under high applied potential or after higher CV cycles, the preservation of ligand peaks in AD-ZIF is notably better than in AC-ZIF. Additionally, there are slightly signals in the range of 498 to 617 cm^{-1} indicating the presence of high-valence cobalt species. Combined with the AE-like core-shell structure observed in TEM images (Figs. S32 and S33), this implies that they undergo the SR process, continuous generation of cobalt (oxy)hydroxide layer while preserving the inherent framework structure as the core. In the case of AB-ZIF, the characteristic peaks rapidly weaken and disappear after 1.30 V or 20 CV cycles, with signals of probably CoOOH appearing only after 80 cycles. Subsequently, no distinct features were observed in the Raman spectra, indicating rapid structural degradation to form the amorphous structures.

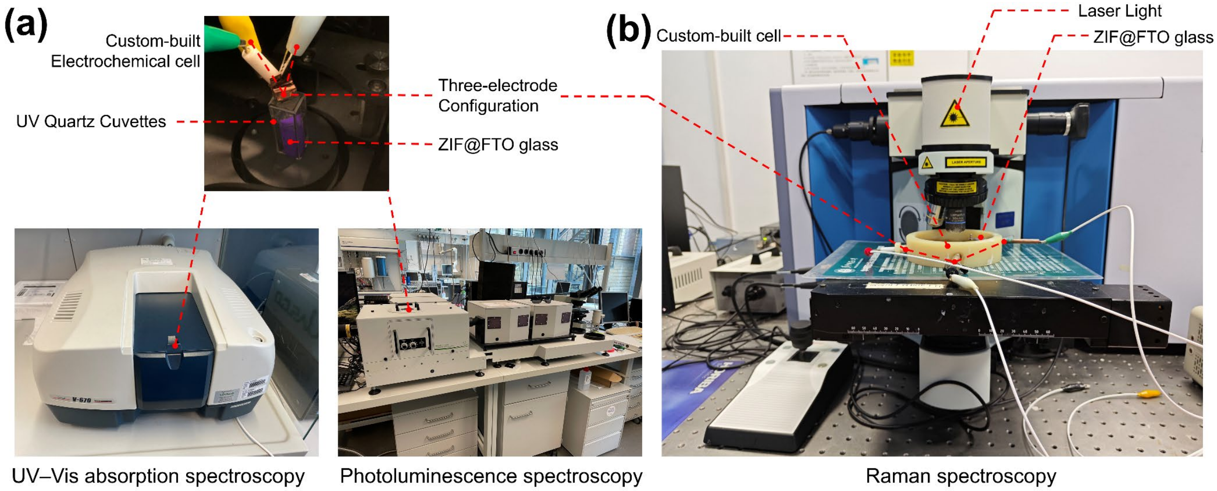

Fig. S34. Experimental setup for In-situ electrochemical UV–Vis absorption/Photoluminescence (**a**) and Raman (**b**) measurements.

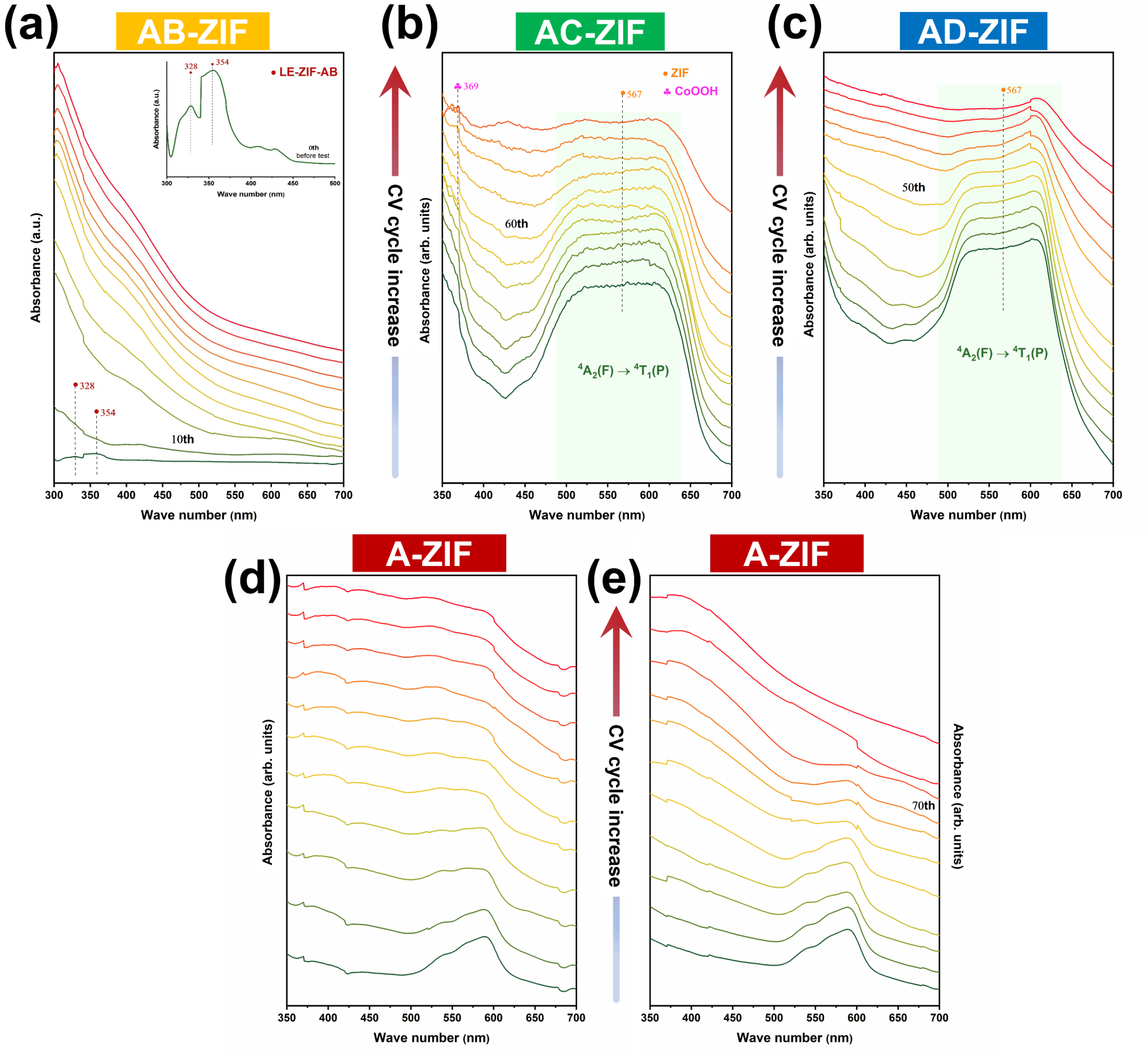

Fig. S35. a, b, **c**, In-situ electrochemical UV–Vis absorption spectroscopy of AB-ZIF (**a**), AC-ZIF (**b**) and AD-ZIF (**c**) with 100 CV cycles at high potential window from 0.85-1.55 V. **d, e**, In-situ electrochemical UV–Vis absorption spectroscopy of A-ZIF with 100 CV cycles at low potential window from 0.85-1.05 V (**d**) and medium potential window from 0.85-1.30 V (**e**).

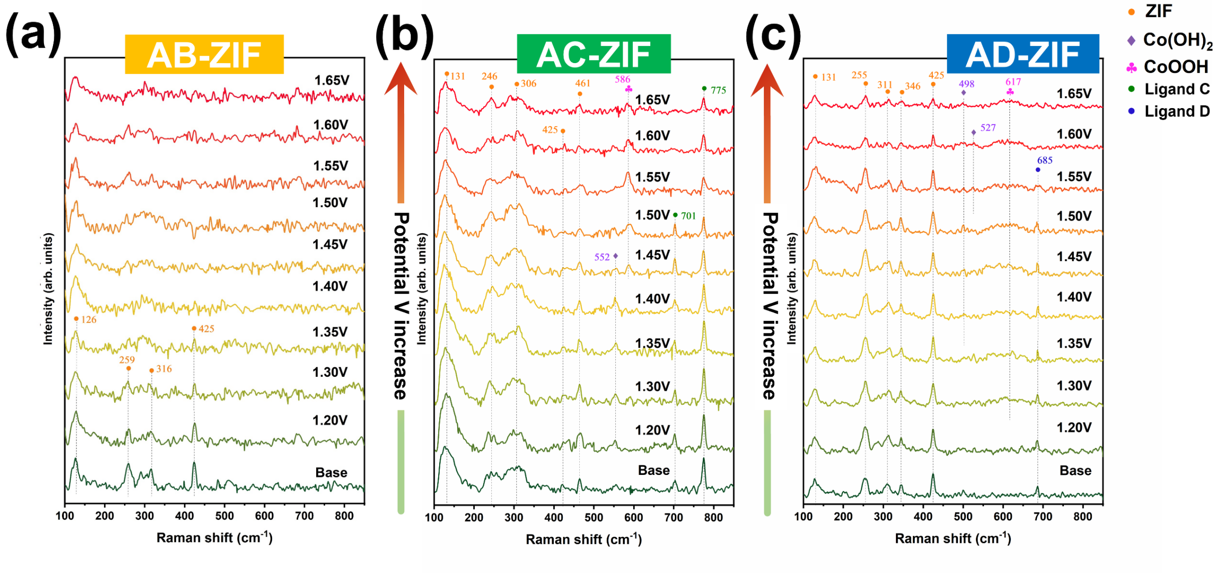

Fig. S36. In-situ electrochemical Raman spectroscopy of AB-ZIF (**a**), AC-ZIF (**b**) and AD-ZIF (**c**) from at various applied potentials 1.20 to 1.65 V.

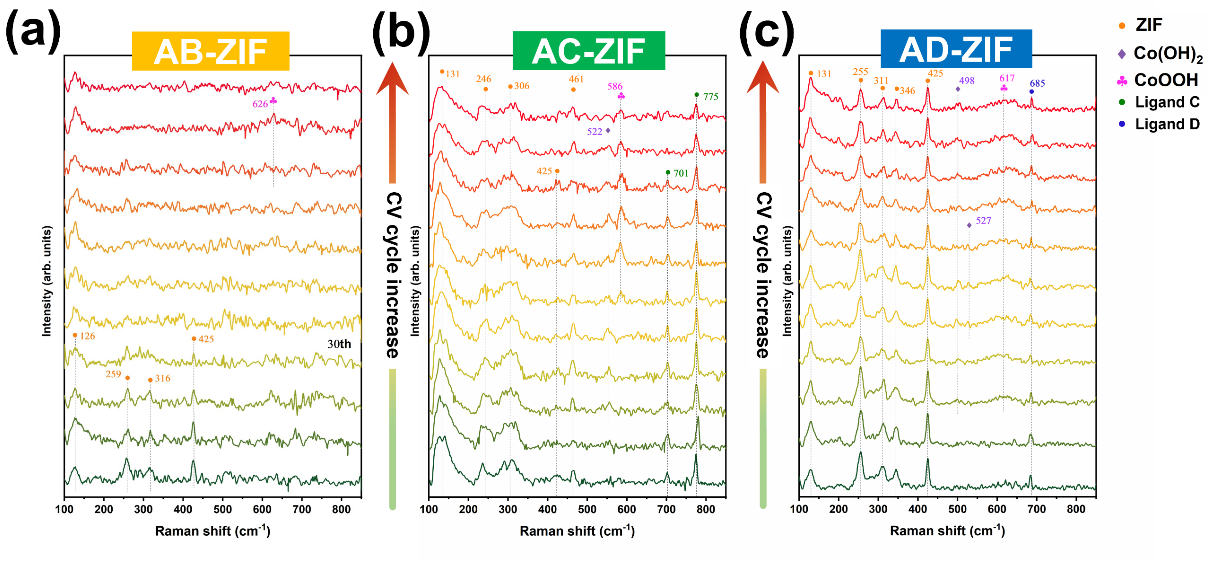

Fig. S37. In-situ electrochemical Raman spectroscopy of AB-ZIF (**a**), AC-ZIF (**b**) and AD-ZIF (**c**) with 100 CV cycles at high potential window from 0.85-1.55 V, the scan rate is 10 mV s^{-1}.

S6. Photoelectrocatalysis and band gaps calculation

Samples AC and AD exhibit similar trends in band gap and PL peak intensity to AE-ZIF, which decrease with an increasing number of CV cycles (Figs. S43 and S45). Specifically, the band gap of AC-ZIF decreases from 1.41 to 1.07 eV, while that of AD-ZIF from 1.43 to 0.91 eV. The decrease in band gap aligns with the varying degrees of self-reconstruction and structural evolution during photoelectrocatalysis. The slight decrease in PL peak intensity of AC and AD can be attributed to the in-situ formation of the cobalt (oxy)hydroxide layer during CV test. Samples AC and AD exhibit relatively low current density and O_2 evolution rate under visible light, with reductions of 23.6% and 23.2% after 12 hours, indicating their inferior activity and stability under photoelectrocatalysis compared to AE-ZIF. Compared to A-ZIF, they still maintain visible light catalytic performance under amperometry for 12 hours, with about 1.5 or 2-fold enhancement compared to the dark condition.

Conversely, AB-ZIF shows poor photoelectrochemical stability, with the PL peak disappearing after 10 CV cycles, indicating a phase transition. Furthermore, AB-ZIF has a band gap exceeding 2 eV and lacks visible light absorption ability, resulting in a negligible enhancement of OER efficiency in the visible light (Fig. S41).

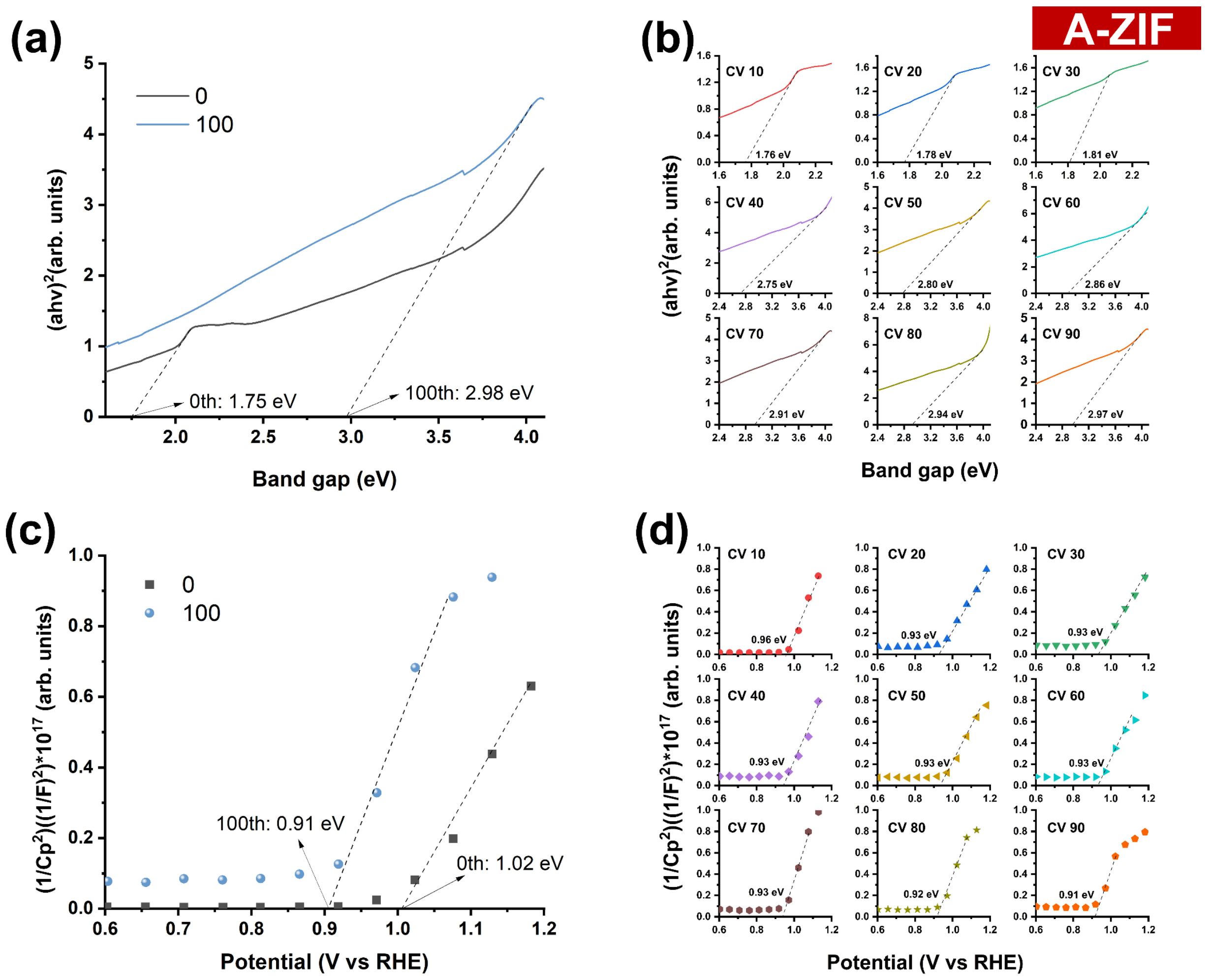

Fig. S38. **a**, **b**, Band gap of A-ZIF calculated from in-situ UV–Vis absorption spectra from 0 to 100 CV cycles at 0.85-1.55 V. **c**, **d**, Flat band potentials of A-ZIF calculated from Mott-Schottky plots from 0 to 100 CV cycles at 0.85-1.55 V.

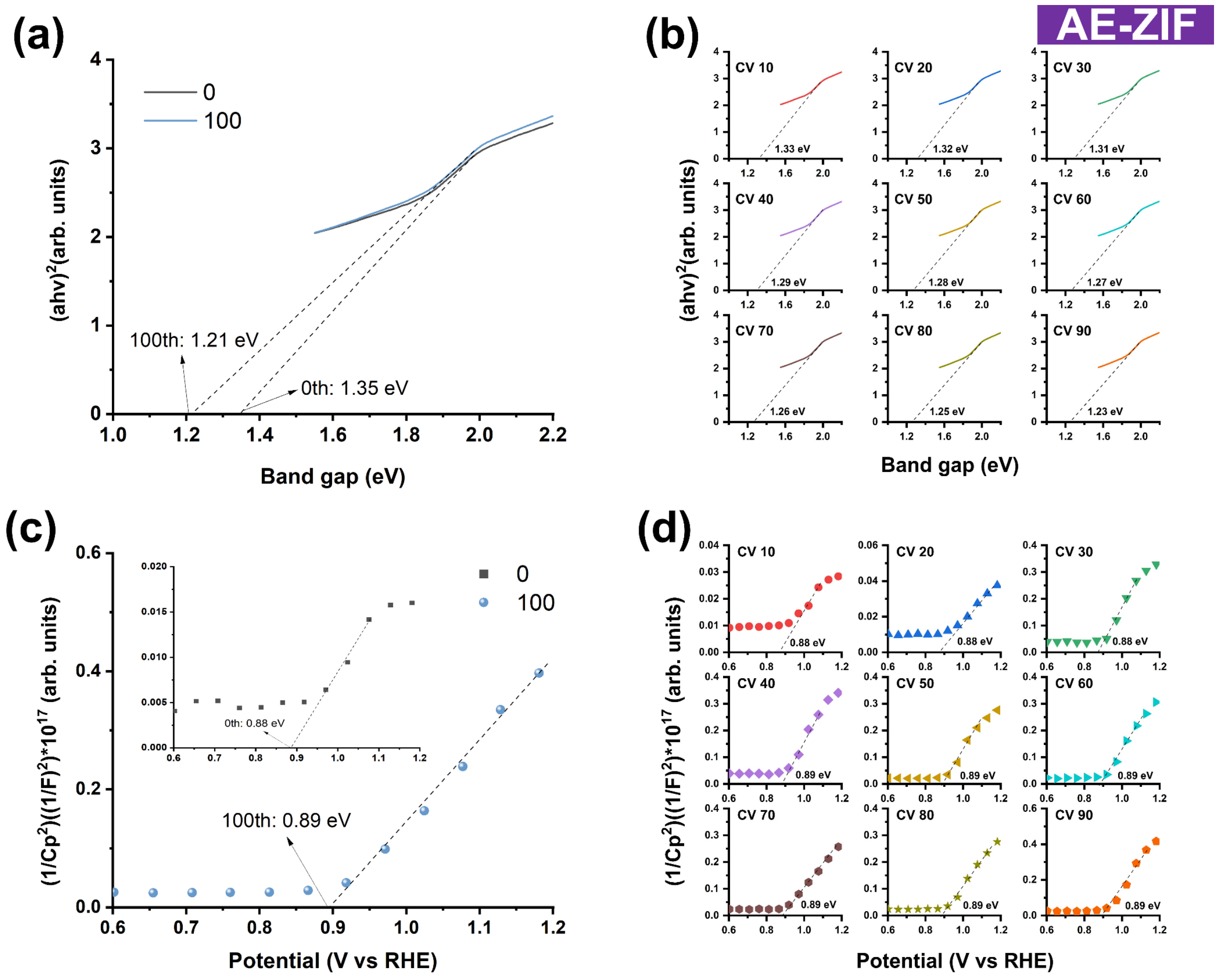

Fig. S39. a, **b**, Band gap of AE-ZIF calculated from in-situ UV–Vis absorption spectra from 0 to 100 CV cycles at 0.85-1.55 V. **c**, **d**, Flat band potentials of AE-ZIF calculated from Mott-Schottky plots from 0 to 100 CV cycles at 0.85-1.55 V.

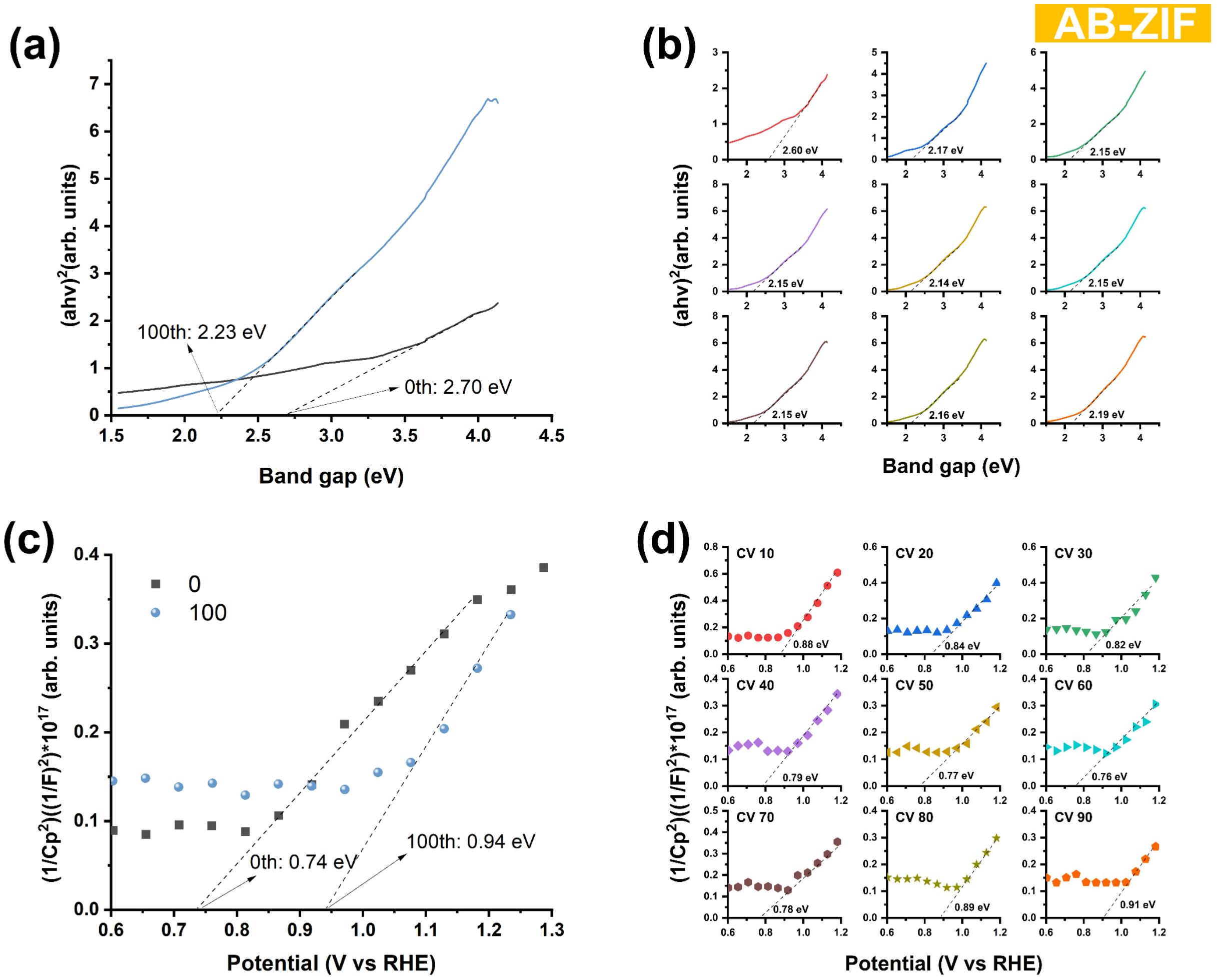

Fig. S40. a, **b**, Band gap of AB-ZIF calculated from in-situ UV–Vis absorption spectra from 0 to 100 CV cycles at 0.85-1.55 V. **c**, **d**, Flat band potentials of AB-ZIF calculated from Mott-Schottky plots from 0 to 100 CV cycles at 0.85-1.55 V.

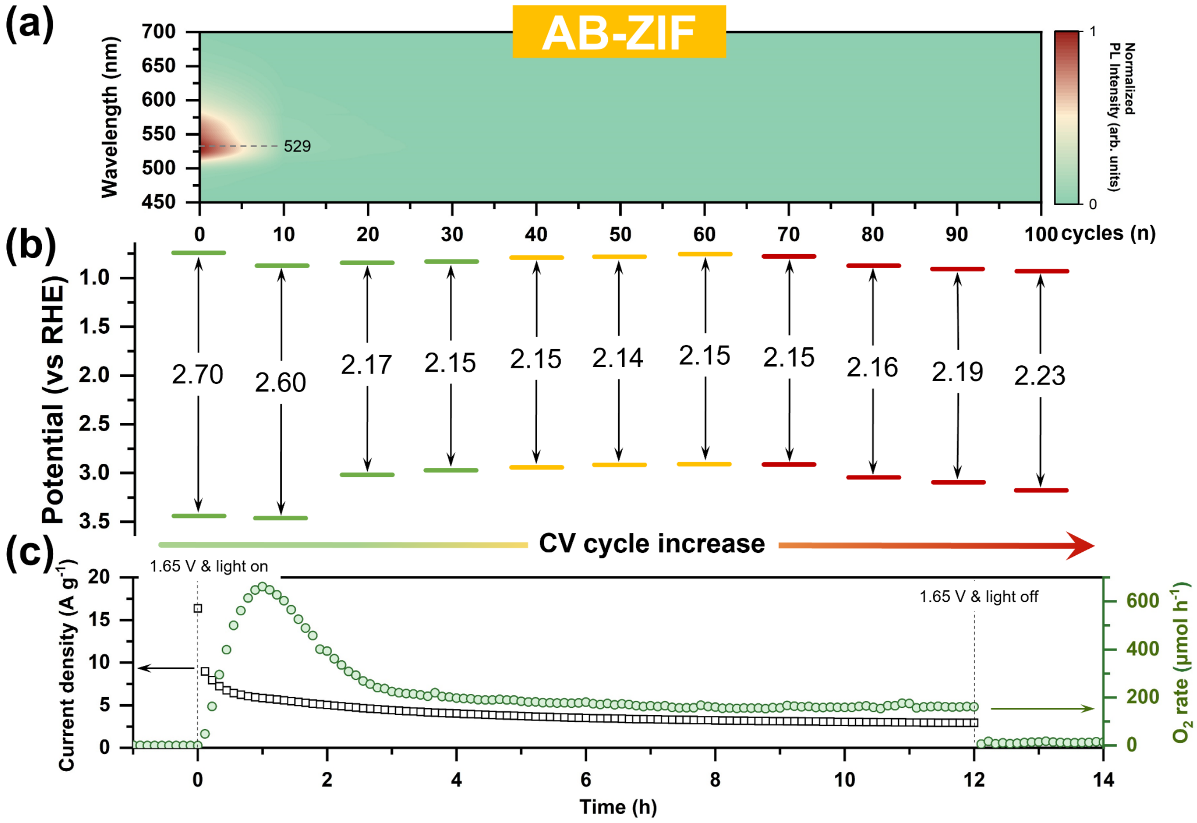

Fig. S41. a, **b**, In-situ electrochemical Photoluminescence (PL) spectroscopy (**a**) and band potential diagram (**b**) of AB-ZIF with 100 CV cycles at 0.85-1.55 V. **c**, Amperometric plots and O_2 evolution rates of AB-ZIF at certain potentials of 1.65 V and visible light.

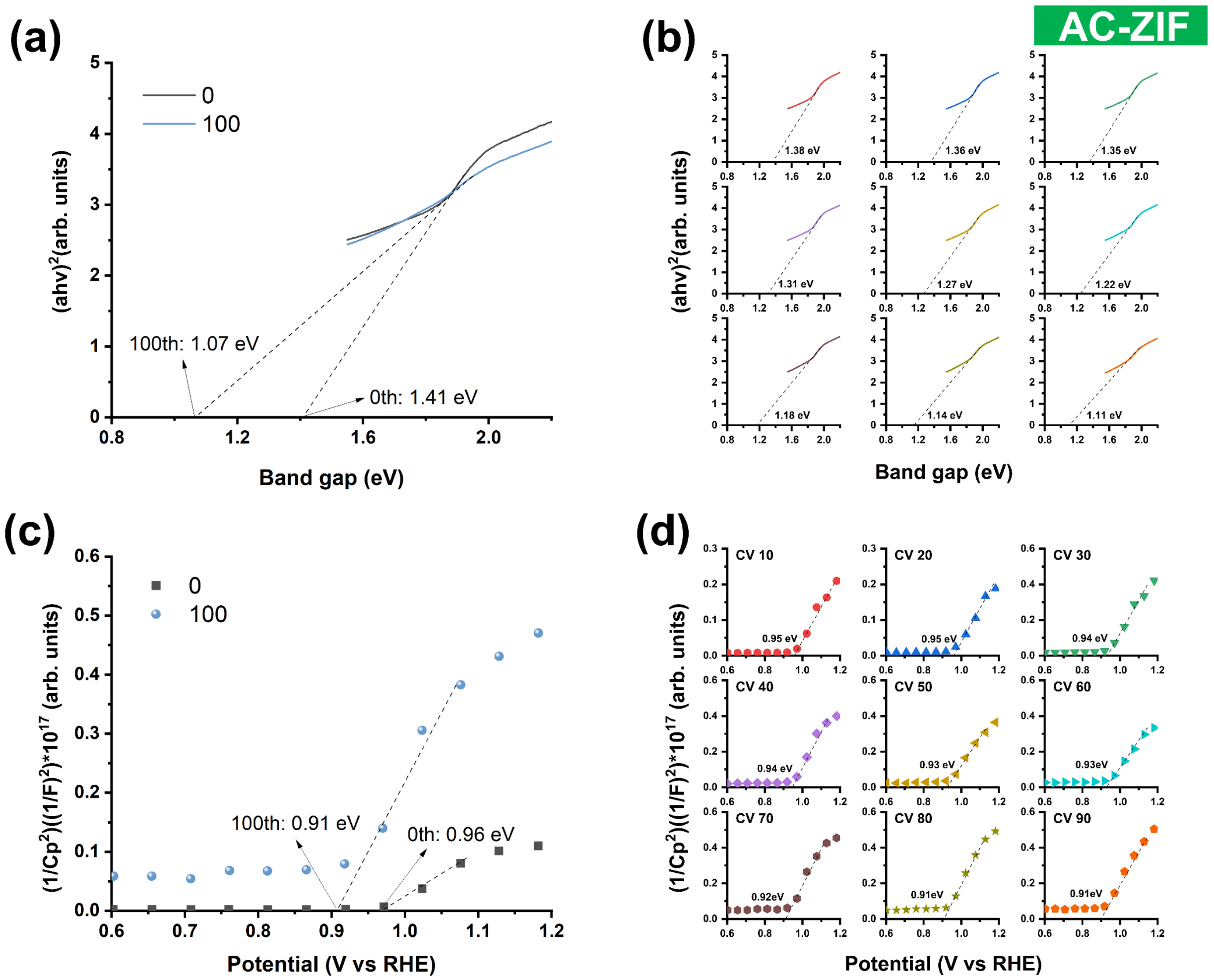

Fig. S42. **a**, **b**, Band gap of AC-ZIF calculated from in-situ UV–Vis absorption spectra from 0 to 100 CV cycles at 0.85-1.55 V. **c**, **d**, Flat band potentials of AC-ZIF calculated from Mott-Schottky plots from 0 to 100 CV cycles at 0.85-1.55 V.

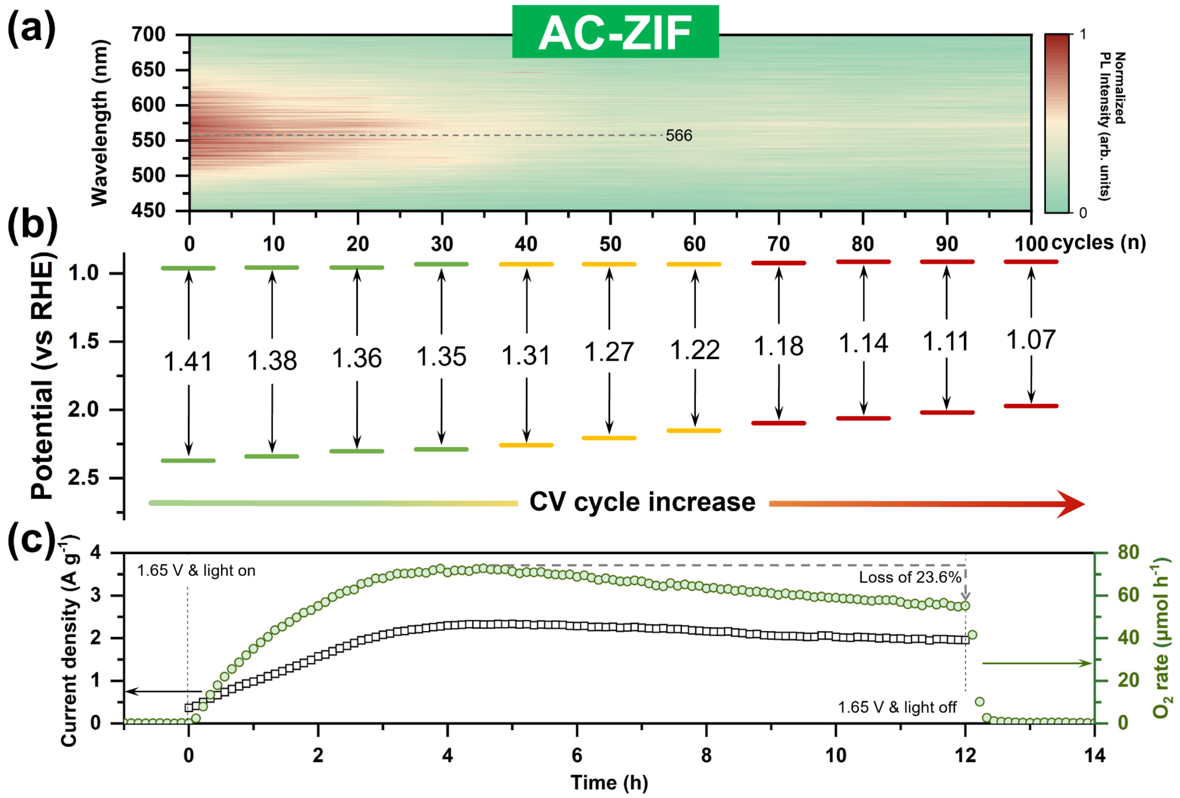

Fig. S43. **a**, **b**, In-situ electrochemical Photoluminescence (PL) spectroscopy (**a**) and band potential diagram (**b**) of AC-ZIF with 100 CV cycles at 0.85-1.55 V. **c**, Amperometric plots and O_2 evolution rates of AC-ZIF at certain potentials of 1.65 V and visible light.

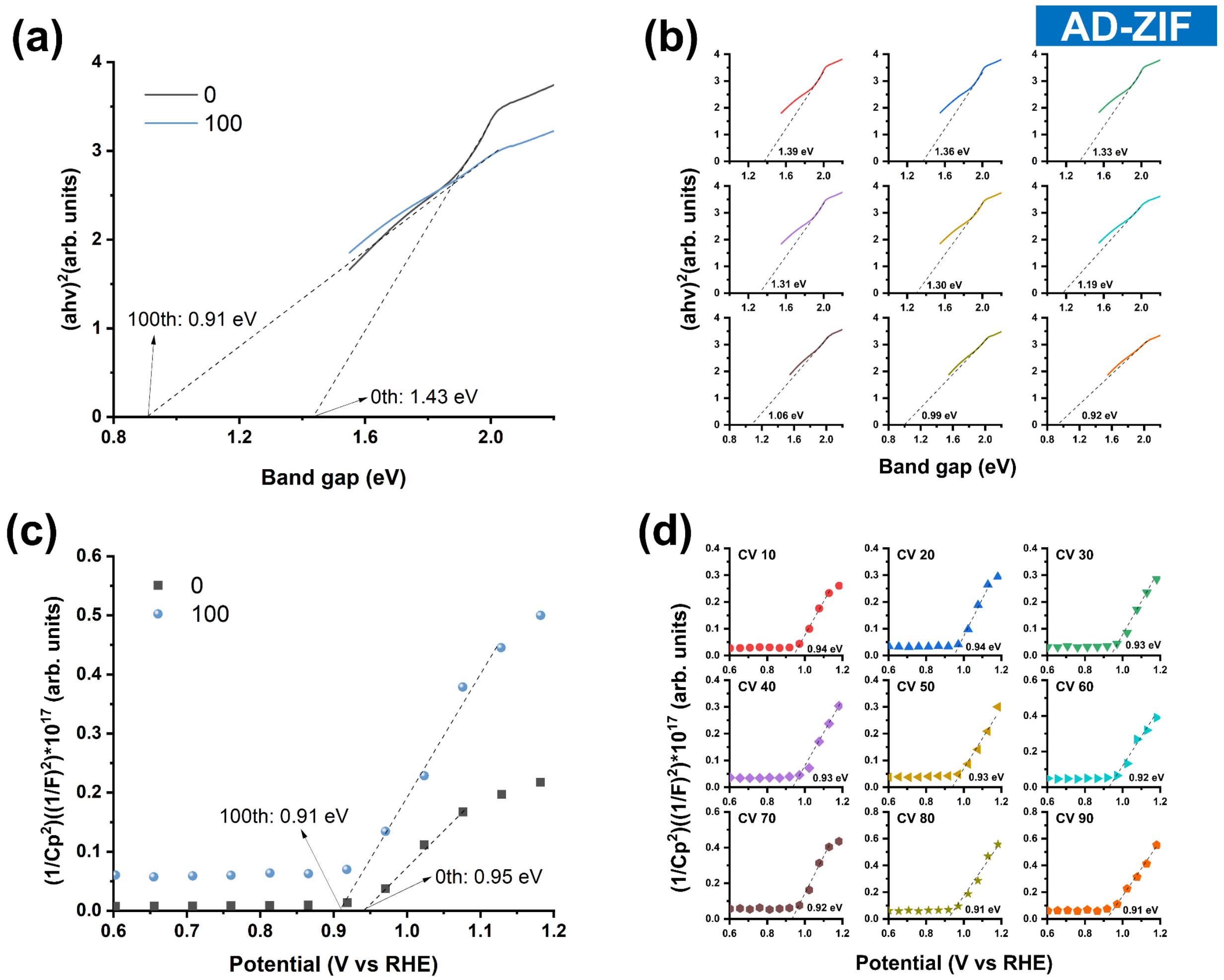

Fig. S44. a, **b**, Band gap of AD-ZIF calculated from in-situ UV–Vis absorption spectra from 0 to 100 CV cycles at 0.85-1.55 V. **c**, **d**, Flat band potentials of AD-ZIF calculated from Mott-Schottky plots from 0 to 100 CV cycles at 0.85-1.55 V.

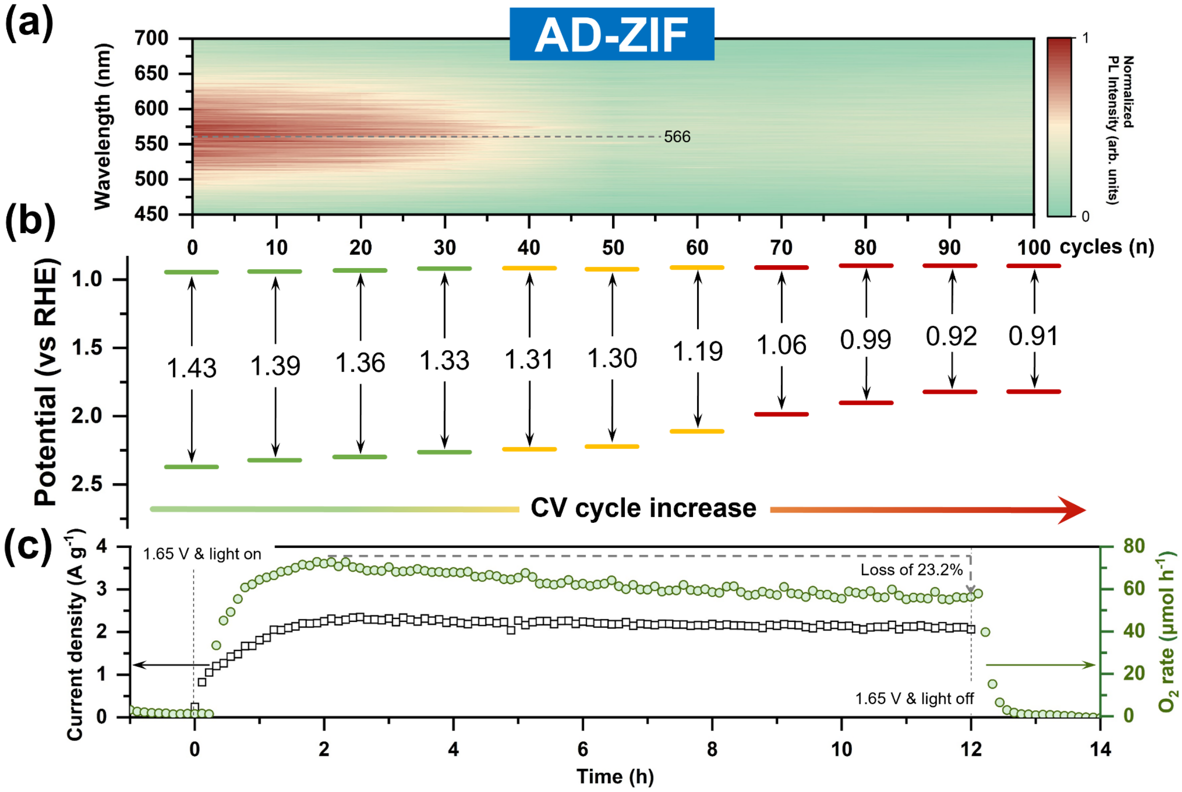

Fig. S45. **a**, **b**, In-situ electrochemical Photoluminescence (PL) spectroscopy (**a**) and band potential diagram (**b**) of AD-ZIF with 100 CV cycles at 0.85-1.55 V. **c**, Amperometric plots and O_2 evolution rates of AD-ZIF at certain potentials of 1.65 V and visible light.

S7. DFT simulation calculations

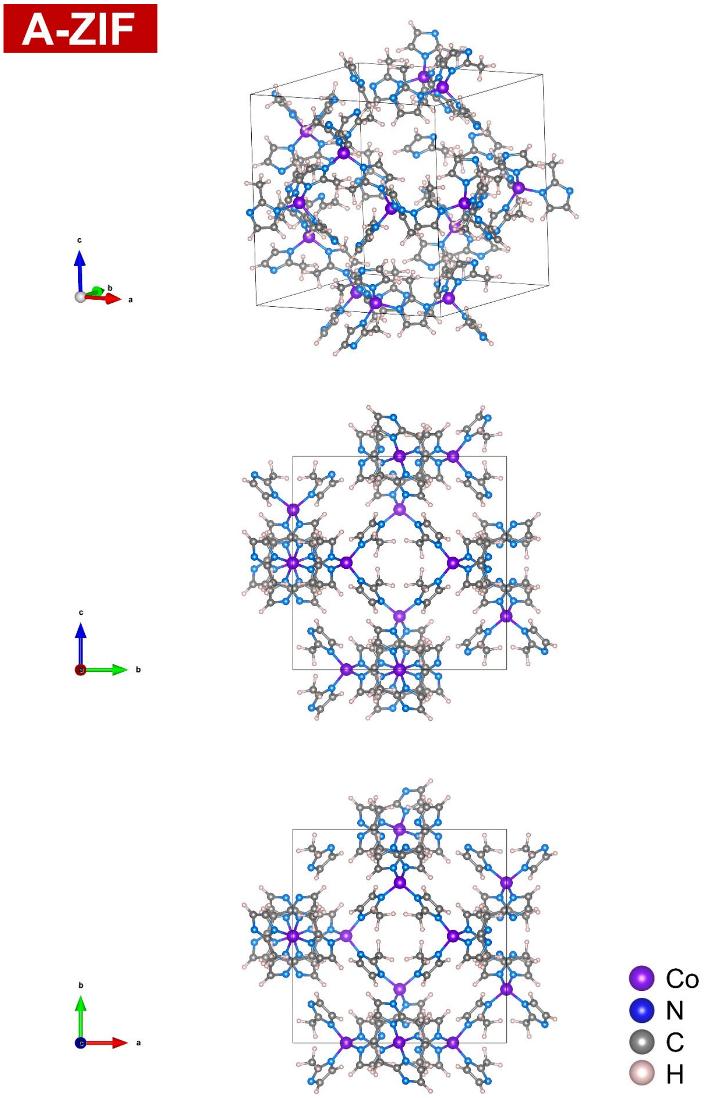

Fig. S46. Models structure of A-ZIF optimized from DFT simulation.

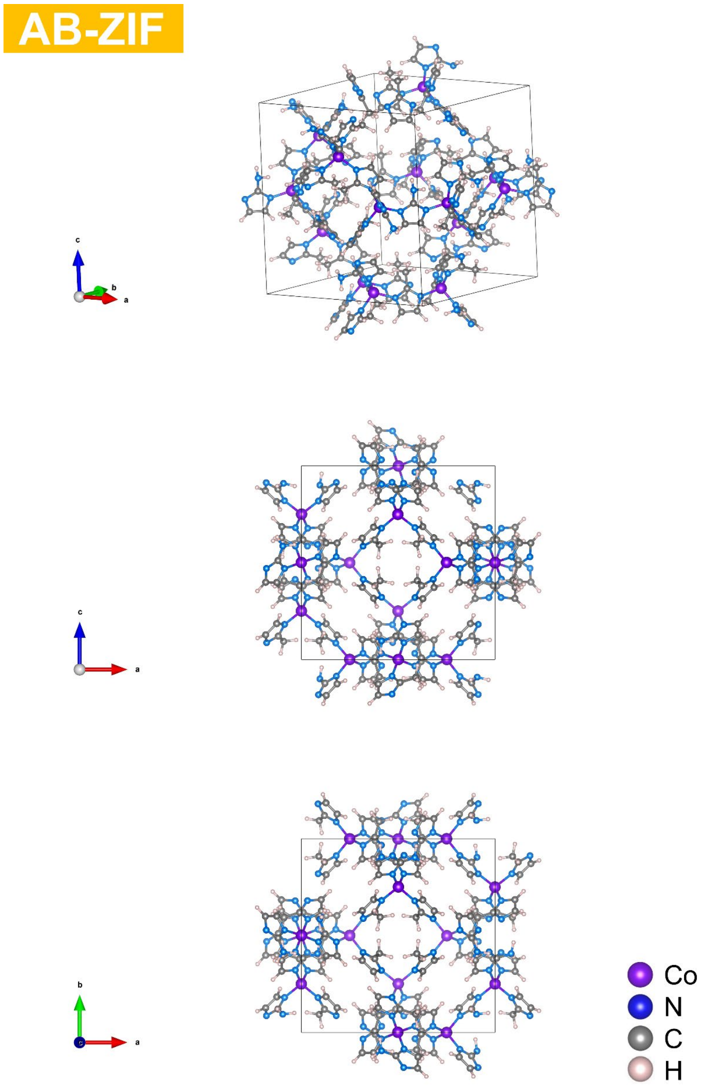

Fig. S47. Models structure of AB-ZIF optimized from DFT simulation.

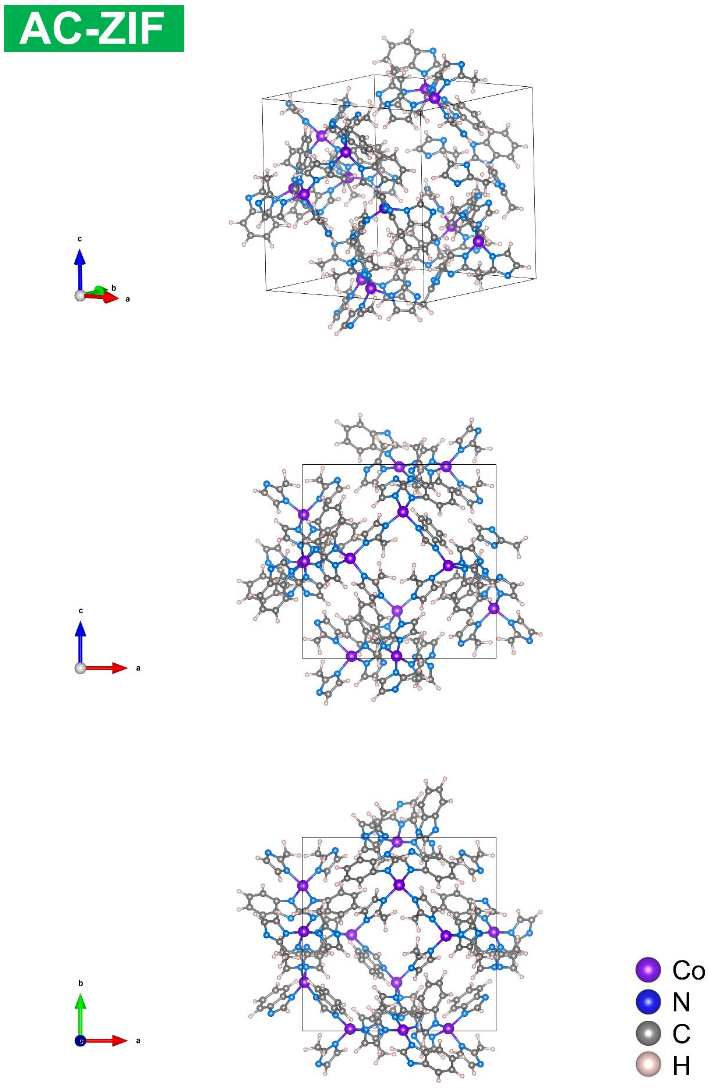

Fig. S48. Models structure of AC-ZIF optimized from DFT simulation.

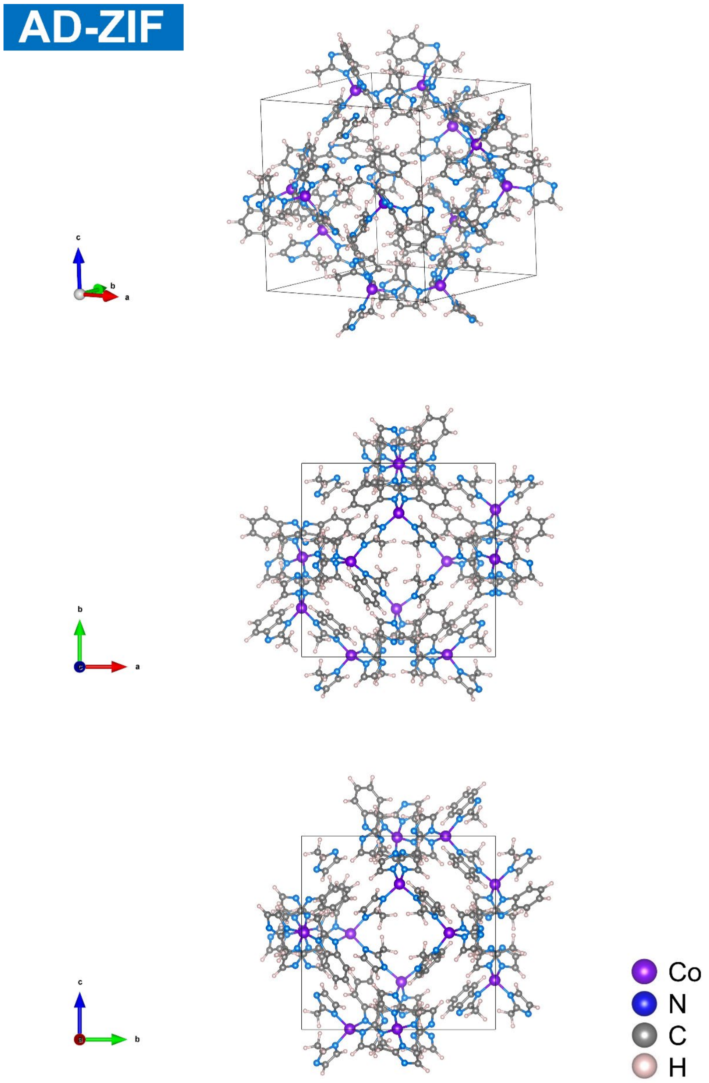

Fig. S49. Models structure of AD-ZIF optimized from DFT simulation.

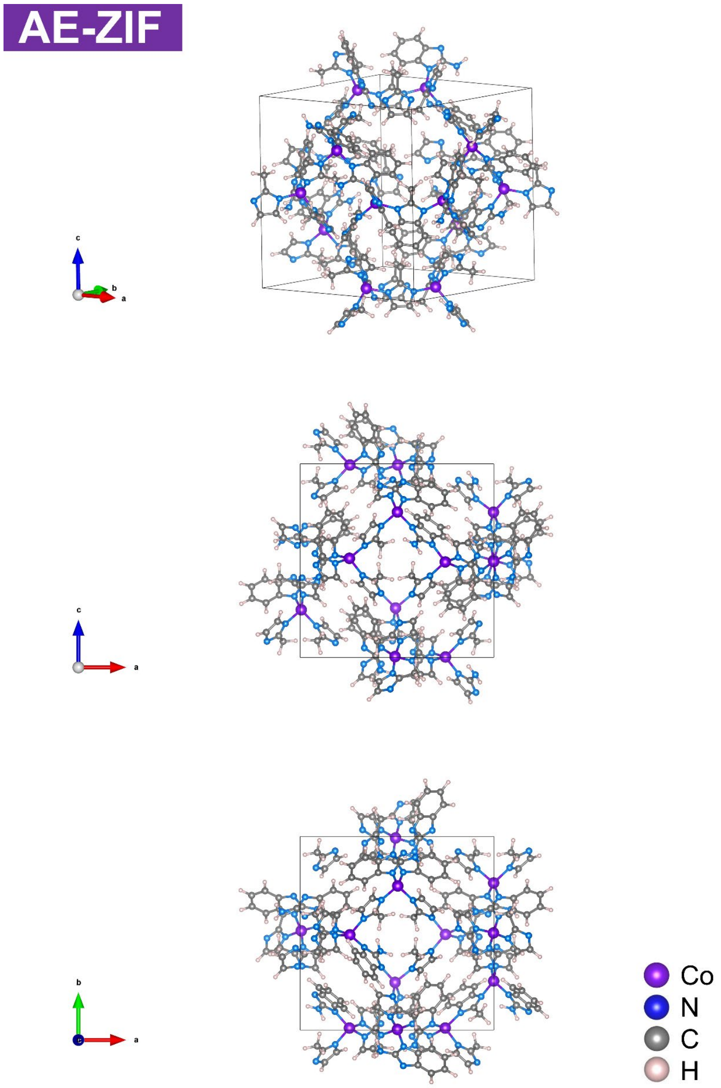

Fig. S50. Models structure of AE-ZIF optimized from DFT simulation.

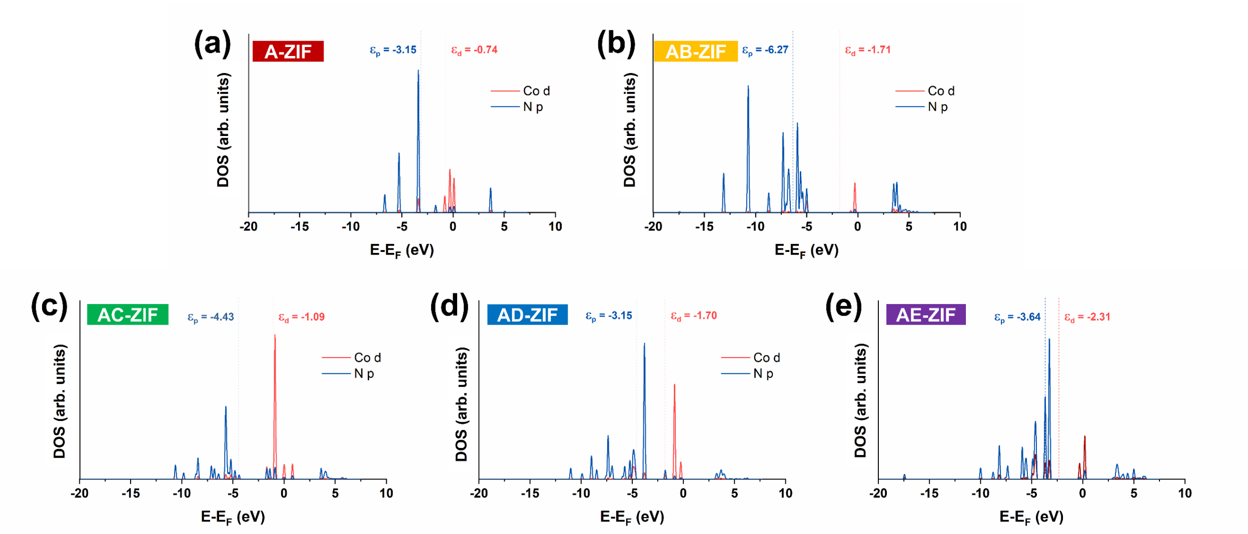

Fig. S51. Calculated DOS of p-state and n-state of A-ZIF (**a**), AB-ZIF (**b**), AC-ZIF (**c**), AD-ZIF (**d**) and AE-ZIF (**e**).

Table S4. Co band center (ε_d), N band center (ε_p) and energy difference (ΔE) between ε_d and ε_p of as-prepared ZIFs.

Samples	ε_d (eV)	ε_p (eV)	ΔE ε_d-ε_p (eV)
A-ZIF	-0.74	-3.15	2.41
AB-ZIF	-1.71	-6.27	4.56
AC-ZIF	-1.09	-4.43	3.34
AD-ZIF	-1.70	-3.15	1.45
AE-ZIF	-2.31	-3.64	1.33

All band center data are from Fig. S51.

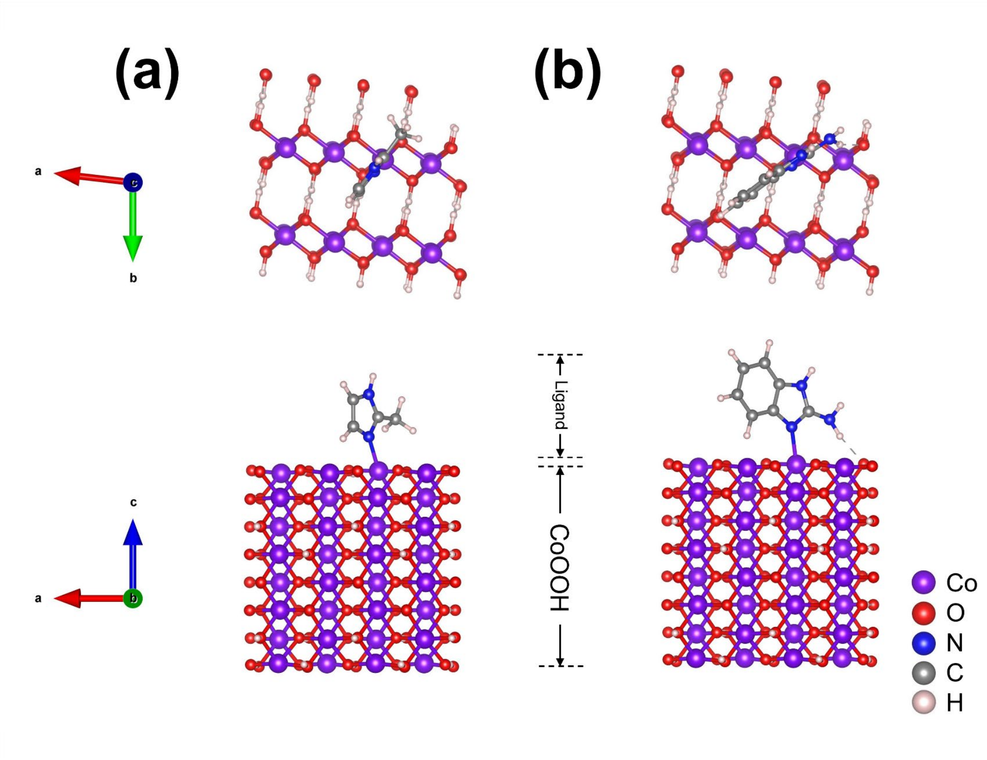

Fig. S52. Models structure of the CoOOH incorporating ligands A (**a**) and E (**b**) optimized from DFT simulation.

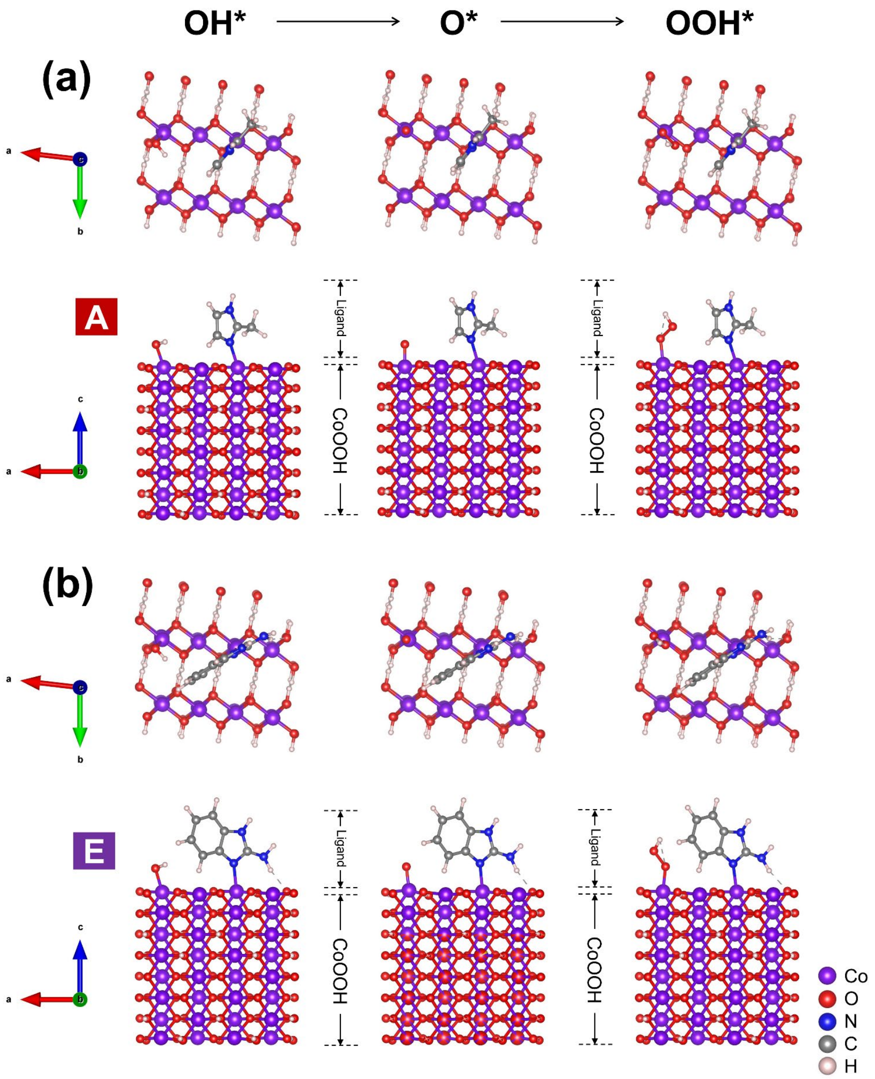

Fig. S53. Calculation steps for OER free energy of A-CoOOH (**a**) and E-CoOOH (**b**).

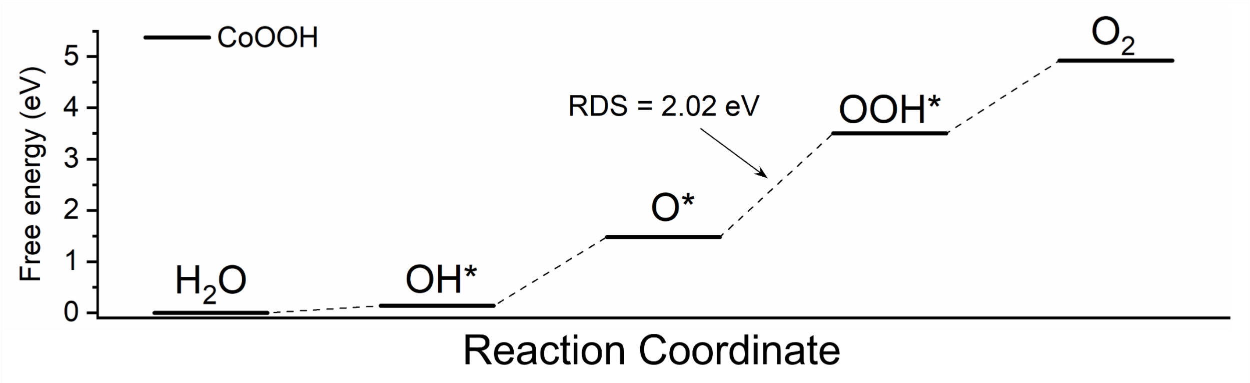

Fig. S54. Free energies diagram of the CoOOH model without ligands.

S8. Characterizations Section

X-ray Diffraction (XRD). Used PANalytical X'Pert Pro multi-purpose diffractometer (MPD) with Bragg Brentano geometry with Cu anode at 45 kV, 40 mA, equipped with a BBHD Mirror and an X-Celerator multichannel detector. The diffraction patterns were tested at a 2θ angle of 5° and 90°. All measurements were performed with Cu sealed tube Kα and Kß radiation (2:1 ratio) with a wavelength of λ=1.54060 Å at a scan rate of 0.5° min^{-1}. Sample holder is a single crystal silicon on which the sample is immobilized by use of a drop of heptane, and rotated every 4 seconds during the test.

X-ray photoelectron spectroscopy (XPS). All XPS measurements were executed using a custom-built SPECS XPS spectrometer equipped with a monochromatized Al-Kα X-ray source (μFocus 350) and a hemispherical WAL-150 analyzer (acceptance angle: 60°). XPS samples were prepared by scratching off the MOF-powder from the FTO substrate and placing it onto In-foil. The In-foil was fixed to the sample holder using double sided carbon tape. For spectral acquisition, pass energies of 100 eV and 30 eV, along with energy resolutions of 1 eV and 100 meV, were employed for survey and detailed spectra, respectively. An excitation energy of 1486.6 eV, beam energy and spot size of 70 W onto 400 μm, an angle of 51° to the sample surface normal were used. The base pressure reached a value of $5x10^{-10}$ mbar, and the pressure throughout the measurement was $5x10^{-9}$ mbar. All reported content values are presented in units of relative atomic percent (at%), with a typical detection limit in survey measurements ranging from 0.1-1 at%, contingent on the specific element. The accuracy of XPS measurements is approximately 10-20% of the reported values.

N_2 Physisorption. Measurements were conducted at temperature of 77 K, on a 3Flex instrument by Micromeritics. Before measuring, the samples were vacuum outgassed at 150°C for 3-12 hours. The apparent surface area was calculated by applying the Brunauer-Emmet-Teller (BET) equation, following the recommended procedure for microporous sorbents [16]. The relevant pore size distributions were determined from the adsorption branch of the isotherms using the kernel of nonlocal density functional theory (NLDFT).

Scanning electron microscope (SEM). Images were recorded on a FEI Quanta 250 (Schottky-)FEG-SEM, facilitated by USTEM (university service center for transmission electron microscopy) at TU Wien, equipped with an ETD Secondary electron detector and an EDAX-AMETEK Octane Elite 55 detector for elemental characterization by energy-dispersive X-ray-spectroscopy. The device was operated at a voltage of 10 kV at a working distance of about 5 mm, providing a maximum resolution of roughly 2 nm. Chemical X-ray microanalysis was performed at 20 kV and 10 mm working distance.

Transmission electron microscope (TEM). All HR-TEM measurements displayed in this paper were performed on a Tecnai F20 FEG-TEM, facilitated by USTEM (university service center for transmission electron microscopy) at TU Wien, equipped with a X-FEG, a Gatan Rio16 CCD-camera, Gatan DigiSTEM II with HAADF detector for STEM imaging, an EDAX-AMETEK Apollo XLTW SDD EDX-detector. The operating voltage was kept at 200 kV for all measurements, providing a maximum lattice resolution of approximately 0.14 nm.

In-situ electrochemical Raman spectroscopy. Measurements were collected on HORIBA LabRAM Spectrometer with a 532 nm laser.

In-situ electrochemical UV–Vis absorption spectroscopy (UV–Vis). Absorbance of each ZIF@FTO glass were obtained at 350-700nm by Jasco V-670. Measurements were carried out using the Jasco V-670 in diffuse reflectance mode with an Ulbricht-sphere.

In-situ electrochemical Photoluminescence spectroscopy. Measurements were carried out with a PicoQuant FluoTime 300 spectrophotometer. The excitation source was a xenon arc lamp (power = 300 W), coupled with a double-grating monochromator. The detection system comprised of a PMA Hybrid 07 detector along with a high-resolution double monochromator. Samples were excited directly at a wavelength of 425 nm with ZIFs@FTO glass.

Nuclear magnetic resonance spectroscopy (^{1}H NMR). Liquid phase ^{1}H spectra were measured using the Bruker ADVANCE 250 (250.13 MHz) instrument, which is equipped with a 5 mm inverse-broad probe head and z-gradient unit. To serve as an internal reference, Acetic Acid with a $\delta = 1.96$ ppm was used. Due to the limited solubility of ZIFs in typical NMR solvents, the sample weighing 2 mg was digested with 0.5 ml of d_4-acetic acid and then sonicated until it was well dispersed in the acid.

S9. References

1. Mor J, Utpalla P, Bahadur J, Sharma SK. Flexibility of Mixed Ligand Zeolitic Imidazolate Frameworks (ZIF-7–8) under CO2 Pressure: An Investigation Using Positron Annihilation Lifetime Spectroscopy. *Langmuir* **38**, 15694-15702 (2022).

2. Hillman F, Zimmerman JM, Paek S-M, Hamid MRA, Lim WT, Jeong H-K. Rapid microwave-assisted synthesis of hybrid zeolitic–imidazolate frameworks with mixed metals and mixed linkers. *J Mater Chem A* **5**, 6090-6099 (2017).

3. Huang Z, Rath J, Zhou Q, Cherevan A, Naghdi S, Eder D. Hierarchically Micro- and Mesoporous Zeolitic Imidazolate Frameworks Through Selective Ligand Removal. *Small* **n/a**, 2307981 (2023).

4. Cho KY*, et al.* Synthesis of amine-functionalized ZIF-8 with 3-amino-1,2,4-triazole by postsynthetic modification for efficient CO2-selective adsorbents and beyond. *J Mater Chem A* **6**, 18912-18919 (2018).

5. Xiang L, Sheng L, Wang C, Zhang L, Pan Y, Li Y. Amino-Functionalized ZIF-7 Nanocrystals: Improved Intrinsic Separation Ability and Interfacial Compatibility in Mixed-Matrix Membranes for CO2/CH4 Separation. *Adv Mater* **29**, 1606999 (2017).

6. Lee YR, Do XH, Cho KY, Jeong K, Baek K-Y. Amine-Functionalized Zeolitic Imidazolate Framework-8 (ZIF-8) Nanocrystals for Adsorption of Radioactive Iodine. *ACS Applied Nano Materials* **3**, 9852-9861 (2020).

7. Schlumberger C, Thommes M. Characterization of Hierarchically Ordered Porous Materials by Physisorption and Mercury Porosimetry—A Tutorial Review. *Adv Mater Interfaces* **8**, 2002181

(2021).

8. Shinagawa T, Garcia-Esparza AT, Takanabe K. Insight on Tafel slopes from a microkinetic analysis of aqueous electrocatalysis for energy conversion. *Scientific Reports* **5**, 13801 (2015).

9. Chen C-L*, et al.* Conductive Lanthanide Metal–Organic Frameworks with Exceptionally High Stability. *J Am Chem Soc* **145**, 16983-16987 (2023).

10. Zhu R*, et al.* Quasi-ZIF-67 for Boosted Oxygen Evolution Reaction Catalytic Activity via a Low Temperature Calcination. *ACS Appl Mater Interfaces* **12**, 25037-25041 (2020).

11. Zuo F*, et al.* Electrochemical interfacial catalysis in Co-based battery electrodes involving spin-polarized electron transfer. *Proc Natl Acad Sci* **120**, e2314362120 (2023).

12. Gao Z, Liu B, Xu X, Peng J, Wu J, Zhang J. Electrochemical oxidation induced in-situ transformation of lamellar cobalt nitrate-hydroxide to oxygen vacancy-rich porous Ce Doped-CoOOH nanosheet for efficient oxygen evolution reaction. *Materials Today Sustainability* **24**, 100601 (2023).

13. El Haskouri J*, et al.* High Cobalt Content Mesoporous Silicas. *Chem Mater* **16**, 2805-2813 (2004).

14. Kamali K, Joseph B, Narayana C. Stability of zeolitic imidazolate frameworks (ZIF-7) under high pressures and its implications on storage applications of ZIFs. *J Solid State Chem* **309**, 122973 (2022).

15. Kamali K, Prasad S, Sahoo MK, Behera JN, Waghmare UV, Narayana C. Unusual CO2 Adsorption in ZIF-7: Insight from Raman Spectroscopy and Computational Studies. *Inorg Chem* **61**, 11571-

11580 (2022).

16. Sánchez-Varretti FO, García GD, Ramirez-Pastor AJ, Romá F. A simple model for studying multilayer adsorption of noninteracting polyatomic species on homogeneous and heterogeneous surfaces. *J Chem Phys* **130**, 194711 (2009).
