# Supplementary material for: Ligand engineering enhances (photo) electrocatalytic activity and stability of zeolitic imidazolate frameworks via in-situ surface reconstruction
Source: Nat Commun. 2024 Oct 30;15:9393. doi: 10.1038/s41467-024-53385-0 (PMC11526130; doi:10.1038/s41467-024-53385-0)
Supplement: Supplementary file 2 — Description of Additional Supplementary Files [file 41467_2024_53385_MOESM2_ESM.pdf]

Description of Additional Supplementary Files

Supplementary Data 1 contains the structures of A-ZIF, AB-ZIF, AC-ZIF, AD-ZIF and AE-ZIF in CIF format (Crystallographic Information Files).

Supplementary Data 2 provides their detailed atomic coordinates, or in other words, their representation in POSCAR format.
