# Supplementary material for: Ligand engineering enhances (photo) electrocatalytic activity and stability of zeolitic imidazolate frameworks via in-situ surface reconstruction
Source: Nat Commun. 2024 Oct 30;15:9393. doi: 10.1038/s41467-024-53385-0 (PMC11526130; doi:10.1038/s41467-024-53385-0)
Supplement: Supplementary file 4 — Supplementary Data 2 [file 41467_2024_53385_MOESM4_ESM.pdf]

Model Data from optimized ZIF models

Ligand Engineering Enhances (Photo)Electrocatalytic Activity and Stability of Zeolitic Imidazolate Frameworks via In-situ Surface Reconstruction

Zheao Huang [1], Zhouzhou Wang [2], Hannah Rabl [1], Shaghayegh Naghdi [1], Qiancheng Zhou [2], Schwarz Sabine [3], Dogukan Hazar Apaydin [1], Ying Yu [,2] and Dominik Eder [*,1]*

[1] Institute of Materials Chemistry, Technische Universität Wien, 1060, Vienna, Austria

[2] Institute of Nanoscience and Nanotechnology, College of Physical Science and Technology, Central China Normal University, 430079, Wuhan, China

[3] Service Center for Electron Microscopy (USTEM), Technische Universität Wien, 1060, Vienna, Austria

* Corresponding authors: dominik.eder@tuwien.ac.at and yuying01@ccnu.edu.cn

A-ZIF:

1.0

16.9076995850	0.0000000000	0.0000000000
0.0000000000	16.9076995850	0.0000000000
0.0000000000	0.0000000000	16.9076995850

N	C	H	Co
48	96	120	12

Direct

0.413717002	0.681653023	0.969488025
0.913477004	0.179631993	0.470624000
0.584508002	0.322443992	0.967615008
0.085841000	0.819966972	0.471657008
0.584779024	0.677917004	0.032224000
0.086806998	0.181274995	0.529121995
0.413206995	0.320874006	0.030006999
0.913120985	0.820795000	0.528966010
0.967481971	0.414669991	0.681330979
0.471652001	0.912631989	0.178575993
0.969632030	0.586467981	0.319296986
0.470537990	0.088229999	0.821406007
0.030406000	0.586871028	0.679512978
0.528452992	0.085589997	0.181496993
0.030262001	0.412896007	0.321725011
0.528815985	0.915247977	0.818093002
0.681109011	0.965866983	0.413441002
0.179508001	0.468546987	0.915368974
0.318430007	0.967800021	0.585947990
0.820716023	0.470227987	0.086874999
0.679453015	0.031925000	0.588066995
0.178565994	0.530176997	0.086175002
0.319519013	0.029638000	0.413206995
0.820836008	0.529609978	0.914120018
0.680603027	0.413735986	0.968953013
0.179694995	0.913286984	0.470901996
0.320266992	0.587535024	0.969394982
0.820406973	0.085198998	0.470681995
0.680373013	0.586085975	0.029436000
0.179412007	0.086539999	0.530390024
0.318374008	0.413477004	0.031831000
0.819935977	0.914817989	0.528576016
0.414824009	0.966756999	0.676953018
0.913680017	0.470618993	0.181326002
0.586606026	0.967882991	0.320369989

0.086907998	0.468098998	0.820330024
0.585238993	0.032295998	0.681394994
0.085855998	0.532224000	0.180873007
0.416229010	0.032483999	0.322535008
0.913788021	0.528240979	0.819703996
0.968625009	0.679167986	0.413866997
0.469769001	0.182530001	0.914494991
0.968200028	0.321749002	0.586916983
0.470607996	0.818885028	0.084916003
0.031128000	0.680109978	0.585385978
0.529322028	0.178141996	0.086574003
0.031256001	0.319472998	0.415666014
0.528317988	0.822090983	0.912324011
0.372781008	0.686012030	0.898218989
0.872573018	0.184003994	0.399488002
0.626367986	0.316835999	0.897010982
0.126948997	0.814544022	0.400642008
0.627188027	0.683004022	0.102348000
0.127452001	0.185683995	0.600642025
0.372640997	0.315277994	0.101548001
0.872021019	0.816157997	0.599920988
0.896418989	0.373288006	0.685459971
0.400391012	0.871949971	0.183781996
0.898293018	0.627095997	0.314745992
0.399105012	0.128758997	0.816326022
0.101643004	0.627490997	0.684404016
0.599702001	0.126763001	0.186150998
0.101668000	0.371982992	0.316816002
0.599924982	0.874027014	0.813944995
0.684717000	0.895125985	0.371773005
0.184426993	0.397056997	0.874747992
0.314359009	0.896569014	0.626919985
0.815796018	0.399327993	0.128162995
0.683857977	0.103174001	0.628702998
0.184318006	0.601772010	0.126677006
0.314893007	0.101191998	0.372671008
0.815629005	0.599967003	0.872071028
0.684885979	0.372889012	0.897709012
0.184413999	0.871524990	0.400274009
0.315820992	0.628458977	0.898202002
0.815719008	0.126168996	0.399419010
0.685482025	0.626756012	0.100982003
0.184043005	0.127802998	0.601109982
0.314770997	0.371901006	0.102548003

0.814934015	0.873727977	0.599865973
0.373151004	0.896120012	0.682761014
0.872681975	0.399430990	0.185699001
0.627133012	0.896308005	0.314801008
0.127923995	0.396970004	0.816621006
0.626438022	0.103523999	0.685813010
0.127560005	0.602831006	0.184515998
0.373748004	0.102665998	0.316922992
0.872476995	0.599343002	0.814487994
0.897755980	0.684015989	0.372364014
0.398635000	0.186468005	0.873272002
0.897023022	0.316354990	0.627792001
0.399612993	0.814584017	0.126451001
0.102096997	0.684634984	0.626932025
0.600085974	0.183367997	0.128077000
0.102015004	0.314916998	0.374237001
0.599548995	0.816901982	0.871457994
0.406437010	0.595404029	0.090723000
0.380546004	0.621104002	0.010704000
0.907496989	0.093217999	0.591376007
0.880647004	0.119117998	0.511756003
0.595106006	0.406989008	0.090207003
0.619113028	0.381743014	0.009448000
0.090533003	0.909151971	0.590644002
0.118744001	0.880818009	0.512261987
0.592325985	0.593692005	0.909539998
0.618629992	0.618628025	0.989709020
0.092671998	0.093253002	0.409162015
0.119406000	0.120139003	0.488642991
0.403093010	0.408571988	0.909693003
0.378847003	0.381393999	0.989741027
0.907338023	0.907871008	0.408376992
0.880316019	0.881231010	0.487641007
0.089680001	0.407716006	0.597185016
0.009008000	0.382055998	0.620921016
0.591441989	0.906431973	0.091480002
0.512112021	0.879305005	0.117822997
0.090636998	0.593918979	0.405813992
0.010350000	0.619262993	0.380208999
0.591387987	0.095747001	0.906572998
0.511331975	0.122079998	0.881582975
0.909323990	0.594531000	0.593393028
0.989633977	0.619937003	0.618891001
0.408712000	0.092267998	0.092265002

0.487940013	0.118106000	0.120178998
0.909348011	0.405155987	0.407898992
0.989646971	0.379689991	0.382393003
0.408363998	0.908838987	0.906459987
0.487542987	0.882475019	0.878795028
0.598051012	0.088885002	0.407218009
0.620851994	0.008059000	0.380977988
0.094595000	0.590466022	0.904594004
0.119718999	0.509613991	0.880940974
0.403086990	0.089486003	0.592433989
0.379857987	0.008470000	0.617729008
0.908340991	0.590927005	0.093734004
0.880877018	0.511645973	0.120269001
0.594142020	0.909932971	0.597038984
0.618968010	0.990806997	0.621349990
0.091741003	0.409613997	0.096693002
0.118308000	0.489919007	0.120663002
0.406830013	0.909537971	0.406686008
0.381610006	0.989997983	0.381435990
0.909015000	0.409561008	0.910049975
0.881367028	0.488014996	0.881303012
0.388579011	0.728474975	0.852846026
0.602630973	0.471120000	0.096695997
0.888870001	0.226594999	0.354126006
0.102387004	0.972666025	0.597033024
0.610571980	0.273858994	0.851853013
0.392594010	0.532603979	0.099432997
0.110728003	0.771695971	0.355868012
0.895084023	0.030073000	0.599820971
0.611724019	0.725862026	0.147643000
0.400400013	0.473396987	0.906228006
0.111585997	0.228476003	0.645711005
0.896508992	0.971467972	0.401549011
0.389495999	0.272193015	0.145926997
0.606440008	0.531131983	0.899626017
0.888148010	0.773850977	0.645546019
0.105673000	0.030122001	0.401362985
0.851215005	0.388366997	0.728492022
0.098993003	0.605255008	0.469215989
0.355446011	0.888611019	0.226558998
0.600378990	0.106587000	0.969886005
0.853579998	0.610801995	0.271753013
0.102453999	0.389133006	0.536372006
0.354153007	0.112092003	0.773563981

0.599084020	0.896129012	0.027884999
0.147064000	0.611730993	0.727087021
0.902696013	0.396806985	0.471870989
0.644924998	0.111275002	0.229049996
0.402619988	0.896543980	0.969944000
0.146706000	0.388195992	0.273972005
0.901808977	0.605252981	0.529878974
0.645828009	0.889708996	0.771986008
0.404516995	0.101055004	0.028085001
0.727122009	0.849439979	0.387230992
0.463645011	0.102856003	0.611419022
0.226960003	0.351787001	0.890528977
0.972733021	0.595476985	0.100683004
0.270907998	0.851742983	0.612091005
0.538186014	0.103376001	0.386976004
0.773123026	0.354173005	0.111601003
0.030849000	0.597820997	0.894648015
0.726810992	0.148138002	0.612578988
0.469938010	0.900523007	0.394488990
0.227660999	0.646215022	0.110224999
0.972181022	0.402496010	0.897135019
0.271665007	0.145892993	0.388444990
0.530067027	0.902981997	0.604466021
0.772687972	0.645089984	0.887612998
0.027093999	0.405176997	0.100414999
0.728049994	0.388460994	0.852876008
0.470335990	0.604496002	0.097690001
0.227219000	0.887353003	0.355040014
0.971301973	0.103468999	0.597710013
0.273321003	0.611676991	0.853013992
0.533146977	0.391344011	0.101727001
0.773606002	0.109940000	0.353745013
0.026681000	0.898690999	0.595786989
0.728827000	0.610790014	0.145610005
0.463476986	0.388839990	0.896754980
0.226356998	0.111119002	0.646525025
0.970758021	0.895991027	0.401400000
0.272439003	0.387176007	0.148450002
0.528346002	0.602536976	0.903276026
0.772005022	0.889684975	0.644825995
0.028774999	0.102860004	0.402787000
0.389310986	0.851206005	0.725839019
0.600504994	0.093202002	0.472005010
0.888598979	0.354241997	0.228279993

0.107980996	0.601127982	0.967197001
0.610692024	0.851980984	0.271481991
0.399015009	0.095059998	0.527859986
0.111553997	0.351568013	0.774110019
0.892789006	0.600338995	0.031420998
0.610502005	0.148727000	0.728345990
0.395837992	0.901703000	0.470138997
0.112074003	0.648802996	0.226655006
0.899604023	0.404911011	0.974168003
0.389306009	0.147860005	0.273873001
0.610035002	0.899055004	0.535049975
0.888778985	0.643979013	0.771408975
0.110659003	0.396845013	0.035944000
0.852454007	0.726783991	0.388071001
0.095017001	0.472283006	0.601553977
0.352836996	0.228676006	0.888828993
0.597643971	0.969851017	0.103822999
0.852106988	0.273312986	0.611261010
0.098699003	0.530595005	0.394075006
0.353745013	0.772355020	0.110983998
0.598205984	0.032357998	0.894339025
0.147141993	0.727476001	0.610606015
0.899725020	0.467689991	0.393188000
0.645366013	0.225949004	0.111832999
0.400635988	0.972441018	0.896223009
0.146948993	0.271881014	0.389705986
0.900386989	0.531418025	0.605772018
0.644572020	0.774208009	0.888264000
0.399078012	0.029622000	0.106037997
0.125111997	0.636345983	0.869714022
0.862327993	0.370559990	0.378816992
0.376646012	0.628884017	0.138086006
0.116424002	0.363480002	0.135547996
0.862389028	0.627463996	0.624421000
0.137737006	0.625790000	0.373847008
0.373870999	0.862828016	0.375328004
0.135407999	0.382153988	0.636004984
0.638427973	0.126991004	0.874059021
0.877658010	0.125926003	0.639236987
0.360522002	0.126534998	0.119672999
0.631716013	0.378592998	0.136100993
0.122249000	0.125527993	0.360825986
0.363801003	0.385226011	0.863184988
0.621351004	0.628225029	0.862464011

0.119465001	0.878472984	0.640377998
0.877371013	0.359860986	0.882040024
0.359914005	0.877614021	0.876038015
0.875989020	0.877277970	0.360136002
0.622895002	0.863933027	0.633216977
0.639644980	0.875604987	0.122680999
0.881711006	0.639254987	0.128478006
0.363633007	0.134663999	0.617983997
0.638818979	0.134017006	0.383718014
0.501546025	0.749994993	0.998440027
0.500433028	0.251194000	0.001566000
0.000768000	0.500168979	0.750371993
0.000490000	0.499282986	0.250461996
0.749100983	0.000205000	0.500613987
0.249647006	0.000085000	0.499716014
0.750061989	0.499642015	0.000012000
0.249431998	0.500827014	0.000694000
0.497521013	0.001925000	0.749413013
0.499487996	0.998292983	0.250820994
0.999258995	0.749673009	0.499720007
0.999454021	0.250604004	0.501558006

AB-ZIF:

1.0

16.9076995850	0.0000000000	0.0000000000
0.0000000000	16.9076995850	0.0000000000
0.0000000000	0.0000000000	16.9076995850

N	C	H	Co
56	88	112	12

Direct

0.414196998	0.681555986	0.968647003
0.913371980	0.180868998	0.469832003
0.585731030	0.322084010	0.967176974
0.086722001	0.819423020	0.471320003
0.585174978	0.679507017	0.031930000
0.085961998	0.180193007	0.530180991
0.413298011	0.320549995	0.031067999
0.912029982	0.819074988	0.529084027
0.969392002	0.416110992	0.680426002
0.470775992	0.912636995	0.179974005
0.968384981	0.586381972	0.319819003
0.468138009	0.086324997	0.821663976
0.032864999	0.585370004	0.677582979
0.529174984	0.086457998	0.181162000
0.030882001	0.412894011	0.320205986
0.530032992	0.914883971	0.818318009
0.680939972	0.965151012	0.414112985
0.180739000	0.467729002	0.914130986
0.318497002	0.966444016	0.585469007
0.820738018	0.469695002	0.086998999
0.679156005	0.032931998	0.587339997
0.177925006	0.532334030	0.085095003
0.319833994	0.030771000	0.413926989
0.818627000	0.528580010	0.912949026
0.681496978	0.413693994	0.967998981
0.180401996	0.912811995	0.471183985
0.319718987	0.588337004	0.969443023
0.819598973	0.086482003	0.470418006
0.680194974	0.587082028	0.029681999
0.179980993	0.086836003	0.530417979
0.318509012	0.413318992	0.032432001
0.818351984	0.913169980	0.528717995
0.413462013	0.966955006	0.677933991
0.913448989	0.469826013	0.181501001
0.586914003	0.967718005	0.320253015

0.088361003	0.468095988	0.818313003
0.585846007	0.032933999	0.681527019
0.086567000	0.532311976	0.181201994
0.414599001	0.032065999	0.321330994
0.913182020	0.528801024	0.819696009
0.968257010	0.680028021	0.413576990
0.468077987	0.180862993	0.914654016
0.968954980	0.322023988	0.585833013
0.471359015	0.817236006	0.087930001
0.031442001	0.680961013	0.585506022
0.529484987	0.180791005	0.087454997
0.032671999	0.320302010	0.415271997
0.528115988	0.819285989	0.910236001
0.904003978	0.094462998	0.585937023
0.906486988	0.904273987	0.414081991
0.082906999	0.407431006	0.594084978
0.585783005	0.903099000	0.092561997
0.413816005	0.093721002	0.096271999
0.414332002	0.905478001	0.905201018
0.095761001	0.584707975	0.900116026
0.907683015	0.416294008	0.909502029
0.372877985	0.685989022	0.897689998
0.873304009	0.183788002	0.398081988
0.627192974	0.316545993	0.896538019
0.127608001	0.814750016	0.400182992
0.627165973	0.684161007	0.102346003
0.127018005	0.185014993	0.601457000
0.372467995	0.314974993	0.102536999
0.871451974	0.815653026	0.600441992
0.898537993	0.373277992	0.686131001
0.399058998	0.872766972	0.183689997
0.897463977	0.627421021	0.315122008
0.397022992	0.127694994	0.816411018
0.103390999	0.627469003	0.682834983
0.600651979	0.127022997	0.185156003
0.102282003	0.372323990	0.315607995
0.601625025	0.874284029	0.814940989
0.684792995	0.894758999	0.371986985
0.184316993	0.395655990	0.874150991
0.314298987	0.895398021	0.627174973
0.815998971	0.398687989	0.127774000
0.684217989	0.103906997	0.628319025
0.183663994	0.603281021	0.126361996
0.315066010	0.102049001	0.372889996

0.814212978	0.599880993	0.871568978
0.685796976	0.372667015	0.896782994
0.184946999	0.871746004	0.400027990
0.315358013	0.629045010	0.898015022
0.815873027	0.126858994	0.398851007
0.685149014	0.627659023	0.101223998
0.184360996	0.127865002	0.601418018
0.314738005	0.371870995	0.103184998
0.814993978	0.873458982	0.600876987
0.372521996	0.896130979	0.683512986
0.872539997	0.398479998	0.185626000
0.627515972	0.896080971	0.314574003
0.128576994	0.396319985	0.815205991
0.627183974	0.104042999	0.685947001
0.127736002	0.603441000	0.184992999
0.372835994	0.102739997	0.316202015
0.872550011	0.600246012	0.815541029
0.897219002	0.684858024	0.372289002
0.397195011	0.185236007	0.873381972
0.898536026	0.316385001	0.628867984
0.399444014	0.813993990	0.128059000
0.102535002	0.685554981	0.626509011
0.601193011	0.183871999	0.127596006
0.103340000	0.315533996	0.373400003
0.600009978	0.815644979	0.870743990
0.406471997	0.595656991	0.090126999
0.380533010	0.621603012	0.010207000
0.879418015	0.121137999	0.511358023
0.596082985	0.407081991	0.089394003
0.620126009	0.381653994	0.008684000
0.090368003	0.908981979	0.589964986
0.119584002	0.879803002	0.512381017
0.592096984	0.594338000	0.909883976
0.618808985	0.619822025	0.989784002
0.092551000	0.093162000	0.409797013
0.119466998	0.119933002	0.489115000
0.402805001	0.408324987	0.910592973
0.378847986	0.380952001	0.990741014
0.878269970	0.878992021	0.487704009
0.008559000	0.383567005	0.618507981
0.512127995	0.877852023	0.120871998
0.089777999	0.594040990	0.405696005
0.009546000	0.619650006	0.380241990
0.589923024	0.095004000	0.906271994

0.509279013	0.120200999	0.881739974
0.910270989	0.594560027	0.593226016
0.990891993	0.619624972	0.617914021
0.487962008	0.120949000	0.121386997
0.910283983	0.405043006	0.407420993
0.990369976	0.379826993	0.381316990
0.488000989	0.880105972	0.877269983
0.598684013	0.088421002	0.407375008
0.620891988	0.007383000	0.381330013
0.121898003	0.509332001	0.878536999
0.402337015	0.088770002	0.592351973
0.379330009	0.007749000	0.617799997
0.908563018	0.589981973	0.093943998
0.880843997	0.510882974	0.120736003
0.594244003	0.910818994	0.597288013
0.619081974	0.991723001	0.621119022
0.091746002	0.410593003	0.096615002
0.118271999	0.491183013	0.120029002
0.406307995	0.910054982	0.406630993
0.380953997	0.990395010	0.381253988
0.879522026	0.487913013	0.879680991
0.389472008	0.728232980	0.852016985
0.603375971	0.471285999	0.096051998
0.889908016	0.225400001	0.351875991
0.102512002	0.972487986	0.595311999
0.611052990	0.273764998	0.851558983
0.392724991	0.532822013	0.098858997
0.110776000	0.772691011	0.354481995
0.611002028	0.726043999	0.148369998
0.399594992	0.473129004	0.907651007
0.111417003	0.228753999	0.645941973
0.898690999	0.963765979	0.406832993
0.389351994	0.272161007	0.147383004
0.606346011	0.531741977	0.900597990
0.888044000	0.773428023	0.646017015
0.105001003	0.029975999	0.401928991
0.854041994	0.389034986	0.729480028
0.097854003	0.605560005	0.469045997
0.352952987	0.890432000	0.224882007
0.599325001	0.106573001	0.969480991
0.852289021	0.611370981	0.272251993
0.095311001	0.392742991	0.536969006
0.352394015	0.111654997	0.773177028
0.148732007	0.611172974	0.725360990

0.903919995	0.396398991	0.471421987
0.645866990	0.110827997	0.227991998
0.147283003	0.388437003	0.272615999
0.901983023	0.605247021	0.529754996
0.648009002	0.891122997	0.773846984
0.408850014	0.098212004	0.036166999
0.727227986	0.848927975	0.386864007
0.462918013	0.102590002	0.611149013
0.226173997	0.350149989	0.891278028
0.972931981	0.594052017	0.101099998
0.271126002	0.850468993	0.611822009
0.538940012	0.103517003	0.387223989
0.773617029	0.353253007	0.111221001
0.726675987	0.149115995	0.611913979
0.469424993	0.901427984	0.394344002
0.227211997	0.647889972	0.110503003
0.965676010	0.407747000	0.895336986
0.271625996	0.146715000	0.388749987
0.530155003	0.903689027	0.604870021
0.771616995	0.645036995	0.887794971
0.027081000	0.406399012	0.100594997
0.728453994	0.388455003	0.851472020
0.470337987	0.604924977	0.096997999
0.227255002	0.888368011	0.354730010
0.963611007	0.100938000	0.593039989
0.273306012	0.612169027	0.852419972
0.534166992	0.391213000	0.100536004
0.772943020	0.110362999	0.353706002
0.026360000	0.899120986	0.594295979
0.728443027	0.611185014	0.145770997
0.463218004	0.389104992	0.896860003
0.226772994	0.111897998	0.646987975
0.272159994	0.387091011	0.148741007
0.528033018	0.602936029	0.903750002
0.773532987	0.890960991	0.646853030
0.028728999	0.103301004	0.403941989
0.388747990	0.851275980	0.726736009
0.601487994	0.092770003	0.472115010
0.888616979	0.353219002	0.228321001
0.104442999	0.595735013	0.958944976
0.610952973	0.851994991	0.270983011
0.398059011	0.094430998	0.527799010
0.113521002	0.351617992	0.771715999
0.893419981	0.599045992	0.031507000

0.611828983	0.149203002	0.729062021
0.395464987	0.902218997	0.470167011
0.112524003	0.648608029	0.227842003
0.388922989	0.148111999	0.273757994
0.610122979	0.899477005	0.535432994
0.889831007	0.646166027	0.773908973
0.110506997	0.396923006	0.036033999
0.851934016	0.727164984	0.388850003
0.095380001	0.464769006	0.607680023
0.352108002	0.228625000	0.888319016
0.592768013	0.962468982	0.101368003
0.853726983	0.273144007	0.612935007
0.098040000	0.530712008	0.393866003
0.353855014	0.771951973	0.111443996
0.597837985	0.031704001	0.894470990
0.147147998	0.729183972	0.611158013
0.900439024	0.467579991	0.393050998
0.646963000	0.225824997	0.110769004
0.406524986	0.964712977	0.897174001
0.148335993	0.272542000	0.389142007
0.901457012	0.531570017	0.606006980
0.645277977	0.773200989	0.887658000
0.120789997	0.629265010	0.867717981
0.863138020	0.370510012	0.378520995
0.376765996	0.629298985	0.137381002
0.116447002	0.364987999	0.136115000
0.863783002	0.627659976	0.624807000
0.136795998	0.626111984	0.373912990
0.373533994	0.863120019	0.375364989
0.636209011	0.127057999	0.873362005
0.875793993	0.121492997	0.632126987
0.367406994	0.123492002	0.121215001
0.632507026	0.378583014	0.135371000
0.121777996	0.125542000	0.361373991
0.363059998	0.385188013	0.864404023
0.621022999	0.628436029	0.862447023
0.118644997	0.879297972	0.640586972
0.874966979	0.367514014	0.895331979
0.366982013	0.875304997	0.882834971
0.882071018	0.875249028	0.366979003
0.623283029	0.865346014	0.633849025
0.632928014	0.873878002	0.116697997
0.882166982	0.638592005	0.128404006
0.362682998	0.133680999	0.618080020

0.639874995	0.132940993	0.383473992
0.500802994	0.751264989	0.999014974
0.500216007	0.251547992	0.000263000
0.001233000	0.501648009	0.750553012
0.000301000	0.500059009	0.250660986
0.749521971	0.999994993	0.501188993
0.249552995	0.999621987	0.499689996
0.750916004	0.499579996	0.000630000
0.249576002	0.500910997	0.000679000
0.497956991	0.001180000	0.749368012
0.499285012	0.998818994	0.250312001
0.999233007	0.750681996	0.498935014
0.999980986	0.250891000	0.501119018

AC-ZIF:

1.0

16.9076995850	0.0000000000	0.0000000000
0.0000000000	16.9076995850	0.0000000000
0.0000000000	0.0000000000	16.9076995850

N	C	H	Co
48	120	120	12

Direct

0.419456005	0.673286974	0.977483988
0.945052028	0.173177004	0.449775994
0.563710988	0.324571997	0.968465984
0.084830001	0.828945994	0.482546002
0.583625972	0.676814020	0.005664000
0.072255000	0.173547998	0.560783982
0.419328004	0.324791014	0.063669004
0.919744015	0.827461004	0.500086010
0.979884982	0.432206988	0.664507985
0.439610988	0.911062002	0.163399994
0.941504002	0.581157029	0.331681997
0.479831010	0.071138002	0.838979006
0.056379002	0.584168971	0.665268004
0.512076974	0.066937000	0.175951004
0.023351001	0.431946993	0.333361000
0.565905988	0.926118016	0.828559995
0.669628978	0.968829989	0.414613008
0.181693003	0.499062985	0.919484973
0.329400986	0.005525000	0.596763015
0.819401979	0.497597009	0.076338001
0.688642025	0.038654000	0.569050014
0.173375994	0.504821002	0.090180002
0.319602013	0.004136000	0.424438000
0.816484988	0.494379014	0.900155008
0.663097978	0.413161993	0.958971977
0.170532003	0.931155980	0.480132997
0.332601011	0.572239995	0.973990023
0.838603020	0.089601003	0.460832000
0.671383977	0.577431977	0.015283000
0.172223002	0.081618004	0.565877974
0.326081008	0.423518986	0.063165002
0.824477971	0.919579983	0.492796004
0.429096997	0.004350000	0.684556007
0.908201993	0.503562987	0.175117001
0.576969028	0.974114001	0.318933994

0.089546002	0.502264023	0.823185980
0.600731015	0.025459999	0.671519995
0.078713998	0.507378995	0.183449000
0.405959994	0.993689001	0.323581010
0.916365981	0.502763987	0.812453985
0.943962991	0.671153009	0.433329999
0.483967006	0.171645001	0.926360011
0.991768003	0.328846008	0.581719995
0.440973997	0.828208983	0.055519000
0.056800000	0.673572004	0.562864006
0.504550993	0.170976996	0.093492001
0.020277999	0.329663992	0.418680996
0.560214996	0.832040012	0.926949978
0.375802994	0.678844988	0.908011019
0.901796997	0.177458003	0.379281998
0.601451993	0.314294010	0.896184027
0.128152996	0.824880004	0.412811011
0.626711011	0.686914980	0.074749999
0.103758000	0.173796996	0.637374997
0.385652989	0.328763992	0.139028996
0.874363005	0.822992027	0.568059027
0.912636995	0.385490000	0.671130002
0.366304010	0.873386979	0.159105003
0.866295993	0.614961028	0.337765992
0.412234992	0.116834998	0.829361022
0.131641001	0.618004978	0.660836995
0.578769028	0.113656998	0.186564997
0.091059998	0.386788994	0.322486997
0.639051974	0.887898028	0.832090020
0.680664003	0.904878020	0.364461988
0.190703005	0.432518989	0.872367978
0.323585987	0.936972976	0.641333997
0.810808003	0.430868000	0.123322003
0.686510980	0.110091001	0.609972000
0.183126003	0.572688997	0.134928003
0.308780015	0.067781001	0.373982996
0.805979013	0.562355995	0.855445027
0.661746025	0.368014991	0.890386999
0.180250004	0.886915028	0.411680013
0.323038012	0.617466986	0.906045020
0.835807025	0.126104996	0.385993987
0.680257022	0.626340985	0.080558002
0.165897995	0.117103003	0.640398979
0.328083992	0.390060991	0.138813004

0.815909028	0.879199028	0.563501000
0.384382010	0.936334014	0.694963992
0.864687979	0.434735000	0.183700994
0.624520004	0.908047020	0.306116998
0.134397000	0.434291989	0.813955009
0.631644011	0.102274999	0.673021019
0.125650004	0.574068010	0.192195997
0.361595988	0.061535001	0.312615991
0.866715014	0.567462981	0.802013993
0.867483020	0.670217991	0.401163995
0.414763987	0.178042993	0.882203996
0.919743001	0.322494000	0.620719016
0.366721988	0.822351992	0.092165001
0.132038996	0.673415005	0.597675979
0.574293017	0.177063003	0.136234999
0.089387000	0.324613988	0.374291986
0.636094987	0.830204010	0.893054008
0.422284991	0.584563971	0.095195003
0.392223001	0.608559012	0.016068000
0.905037999	0.118928000	0.494318008
0.588465989	0.411511004	0.088155001
0.602976024	0.385102987	0.005282000
0.080849998	0.915573001	0.601297975
0.111213997	0.893707991	0.521633983
0.582406998	0.580227017	0.893397987
0.611483991	0.609990001	0.971072018
0.114427000	0.116774000	0.522450984
0.382310987	0.383085012	0.022235001
0.919331014	0.912321985	0.377481997
0.888583004	0.887145996	0.455978990
0.106861003	0.420630008	0.582068026
0.026714999	0.395659000	0.608748972
0.480048001	0.882939994	0.099777997
0.983883977	0.615821004	0.390578985
0.600742996	0.081495002	0.929708004
0.521817982	0.106243998	0.899375021
0.015659001	0.618609011	0.604930997
0.388491988	0.080825001	0.088993996
0.468116999	0.104068004	0.118472002
0.902468979	0.418177009	0.423709005
0.981655002	0.395056009	0.392919987
0.522557020	0.890685022	0.886563003
0.574468970	0.086094998	0.415805995
0.605570018	0.009500000	0.385214001

0.089440003	0.616324008	0.919862986
0.119359002	0.539928973	0.887964010
0.420518011	0.123604998	0.594586015
0.394026995	0.044953998	0.624216020
0.907166004	0.618291974	0.079089001
0.879464984	0.540301979	0.109311000
0.635948002	0.991451979	0.607999027
0.077813998	0.389907986	0.091498002
0.109274998	0.466729999	0.120737001
0.409361988	0.884459019	0.425226986
0.379485995	0.960424006	0.392259002
0.915641010	0.383150011	0.902157009
0.884006977	0.459780991	0.872574985
0.708536983	0.901530027	0.789254010
0.774752975	0.856185973	0.807654977
0.772275984	0.799291015	0.868463993
0.703602016	0.785830975	0.912003994
0.201337993	0.713937998	0.577280998
0.269261986	0.698507011	0.621111989
0.268997997	0.643158019	0.683318973
0.200721994	0.601934016	0.703544974
0.298460990	0.779915988	0.071781002
0.231850997	0.788497984	0.119542003
0.232019007	0.838395000	0.186132997
0.298851013	0.881951988	0.206282005
0.796531975	0.598771989	0.296299994
0.728129983	0.638653994	0.319063991
0.728973985	0.692502975	0.382351011
0.798012018	0.708900988	0.424241006
0.915988028	0.218912005	0.308914006
0.862801015	0.209237993	0.246685997
0.796863973	0.158920005	0.253717005
0.782826006	0.116508998	0.322939008
0.725035012	0.181963995	0.595429003
0.707301974	0.245719999	0.644263029
0.651885986	0.238491997	0.705901027
0.613518000	0.167282999	0.721125007
0.206384003	0.102320999	0.711156011
0.184529006	0.145292997	0.777984977
0.122703001	0.200997993	0.775083005
0.081324004	0.215633005	0.705348015
0.403140008	0.286482006	0.208146006
0.362271011	0.306432992	0.276713014
0.306095988	0.367911994	0.276944011

0.288619995	0.410596997	0.208761007
0.385993004	0.725736022	0.865324974
0.596804023	0.475546002	0.093883999
0.091104999	0.978030980	0.614252985
0.582063973	0.268949986	0.855368972
0.430895001	0.520479977	0.099299997
0.118681997	0.778258979	0.369657010
0.616102993	0.736626029	0.113958001
0.905174017	0.974669993	0.366360992
0.600975990	0.518966019	0.883744001
0.886686981	0.779873013	0.614023983
0.864952981	0.401163012	0.710888982
0.611869991	0.110758997	0.986495018
0.118701003	0.395229012	0.523576975
0.368007004	0.102457002	0.785309017
0.899331987	0.407478005	0.487661004
0.624100983	0.097745001	0.229013994
0.134809002	0.402527988	0.278436989
0.386108994	0.085750997	0.024240000
0.727643013	0.862353027	0.374208987
0.481501013	0.135536999	0.613210022
0.236194998	0.388660997	0.883803010
0.971966028	0.622627020	0.082321003
0.276719004	0.894101977	0.631722987
0.513275027	0.094217002	0.395794004
0.767268002	0.385522008	0.110175997
0.024645999	0.619098008	0.915144026
0.473325998	0.878551006	0.415021002
0.229891002	0.614844024	0.122556999
0.979021013	0.378156006	0.888764024
0.264566988	0.112972997	0.385331005
0.757098019	0.602692008	0.865625024
0.013407000	0.387300998	0.100102000
0.704133987	0.378257990	0.843423009
0.478884012	0.614148974	0.106366999
0.223308995	0.903189003	0.367128998
0.279247999	0.602612972	0.861720979
0.528689027	0.395842999	0.107097998
0.017143000	0.903111994	0.604633987
0.723586023	0.614185989	0.126267001
0.983780980	0.904679000	0.374940008
0.517552018	0.583611012	0.890466988
0.769954979	0.893598020	0.605850995
0.399540007	0.892364025	0.739251018

0.576738000	0.088643998	0.480536014
0.874933004	0.394042999	0.232235000
0.106393002	0.622305989	0.982249975
0.614781022	0.868192017	0.256635010
0.417288989	0.126084998	0.529722989
0.122725002	0.392051011	0.767095029
0.888323009	0.626417994	0.017444000
0.397745013	0.881483972	0.489012986
0.115061000	0.617538989	0.238261998
0.906955004	0.378275007	0.966372013
0.371318012	0.100791998	0.262890011
0.878306985	0.612394989	0.757534027
0.091181003	0.382773012	0.028297000
0.112019002	0.485045999	0.578406990
0.372725010	0.225504994	0.891993999
0.879244030	0.274008989	0.609201014
0.604525983	0.017347001	0.938035011
0.889958978	0.480468005	0.412054986
0.614965022	0.225948006	0.127571002
0.132299006	0.277608991	0.383118987
0.373407990	0.020383000	0.106141001
0.113770001	0.667411983	0.887403011
0.855279982	0.382937998	0.395752013
0.380926013	0.601511002	0.142591000
0.104162998	0.339347988	0.123305999
0.380192995	0.833262980	0.397161990
0.153476998	0.399430007	0.622355998
0.648431003	0.098448001	0.888606012
0.342628986	0.120526001	0.112885997
0.629908025	0.382995993	0.129736006
0.605627000	0.616469026	0.844365001
0.109876998	0.880380988	0.647921979
0.885280013	0.332522005	0.874360979
0.893379986	0.877189994	0.329122990
0.882395983	0.667379022	0.114211001
0.383186013	0.171666995	0.617959023
0.609130025	0.136611998	0.393258005
0.462137997	0.908172011	0.899932981
0.710429013	0.947237015	0.743852973
0.829843998	0.865683019	0.775309026
0.825639009	0.765748024	0.882610023
0.703356981	0.743367016	0.960376024
0.202420995	0.755052984	0.527776003
0.324167997	0.728875995	0.606037021

0.323668003	0.631253004	0.715582013
0.201057002	0.556986988	0.749566019
0.954819024	0.603129983	0.590542972
0.539466977	0.902885973	0.085198000
0.296629012	0.742617011	0.019386001
0.178020000	0.756250978	0.104524001
0.178512007	0.844148993	0.221852005
0.299136013	0.923027992	0.255813986
0.795432985	0.554890990	0.249239996
0.672823012	0.626892984	0.287916005
0.674076021	0.721503019	0.399834007
0.797241986	0.748943985	0.474631011
0.044881001	0.600094020	0.403061986
0.968052983	0.256206006	0.302478999
0.873014987	0.240245998	0.190918997
0.756762028	0.151939005	0.203520000
0.733498991	0.075094998	0.326936007
0.925149977	0.101760998	0.552824020
0.766502023	0.187616006	0.546463013
0.735660017	0.302778989	0.633840978
0.637567997	0.290434003	0.741299987
0.569121003	0.162531003	0.767556012
0.623594999	0.931230009	0.589249015
0.252348006	0.057248000	0.713782012
0.214870006	0.134581998	0.834035993
0.105981000	0.232577994	0.828894019
0.031920999	0.256976992	0.704433024
0.102790996	0.100397997	0.461405993
0.448455989	0.240734994	0.207844004
0.375247985	0.274830997	0.331510991
0.276695013	0.383226007	0.332305998
0.246779993	0.459524989	0.210246995
0.397599012	0.396611005	0.961341977
0.500279009	0.753408015	0.988563001
0.487058997	0.249100998	0.010846000
0.007244000	0.512733996	0.741132021
0.989138007	0.509769976	0.256366998
0.754342973	0.011122000	0.477467000
0.247290000	0.009424000	0.515806019
0.742389977	0.490411013	0.990022004
0.254426986	0.495829999	0.009926000
0.520362020	0.003655000	0.755752027
0.489585012	0.975741982	0.244237006
0.003079000	0.749337971	0.493523002

0.011423000	0.250625014	0.501003027

AD-ZIF:

```
1.0
        16.9076995850         0.0000000000         0.0000000000
         0.0000000000        16.9076995850         0.0000000000
         0.0000000000         0.0000000000        16.9076995850
    N     C     H    Co
   48   128   136    12
Direct
     0.418595999         0.669851005         0.966355026
     0.930362999         0.174312994         0.453159988
     0.582262993         0.314503998         0.985508025
     0.081592999         0.840269029         0.478471994
     0.583967984         0.670768023         0.027719000
     0.075053997         0.176156998         0.531907022
     0.419999003         0.328691989         0.046386000
     0.914569020         0.832885981         0.524349988
     0.979426026         0.420870990         0.674969971
     0.481646001         0.909677982         0.182660997
     0.966863990         0.598848999         0.314678997
     0.476112008         0.077862002         0.827130020
     0.020173000         0.592036009         0.680140018
     0.512962997         0.078436002         0.177171007
     0.019459000         0.426766992         0.322023988
     0.528267026         0.912980974         0.822355986
     0.675886989         0.977838993         0.419627011
     0.177272007         0.484598994         0.909731984
     0.333041012         0.964399993         0.578221977
     0.826901972         0.479285002         0.086724997
     0.678435028         0.029564001         0.587885976
     0.178992003         0.519478023         0.075291000
     0.326074004         0.035974000         0.422452986
     0.816143990         0.520947993         0.914798975
     0.674018979         0.409628987         0.979758024
     0.182814002         0.924308002         0.490162998
     0.322640985         0.578643024         0.968622983
     0.827403009         0.087672003         0.460124999
     0.679193020         0.579351008         0.025797000
     0.179374993         0.091614000         0.523714006
     0.330017000         0.428629011         0.045600001
     0.818666995         0.923638999         0.521821976
     0.418776006         0.970888019         0.678867996
     0.923628986         0.483689010         0.175900996
     0.578522980         0.967601001         0.330531001
```

0.072479002	0.471762002	0.829291999
0.573777974	0.037453000	0.671571016
0.082368001	0.531434000	0.164129004
0.419512987	0.033341002	0.328211010
0.915075004	0.530943990	0.827410996
0.966003001	0.694130003	0.410351992
0.477798015	0.173982993	0.918178022
0.983446002	0.329190999	0.579280972
0.479521006	0.814525008	0.087421000
0.023855001	0.687638998	0.585570991
0.518706977	0.169496998	0.081596002
0.017073000	0.330448985	0.413347006
0.531166971	0.815981984	0.915112972
0.372987002	0.678990006	0.898727000
0.884512007	0.183897004	0.385628015
0.622475028	0.307666987	0.914499998
0.126595005	0.835566998	0.410194010
0.627754986	0.678463995	0.096693002
0.115882002	0.179148003	0.603134990
0.377353996	0.325388014	0.116663001
0.870079994	0.824730992	0.592700005
0.909866989	0.377342999	0.677815020
0.406937987	0.874859989	0.183394000
0.889667988	0.627605975	0.321085006
0.407676995	0.122396998	0.818803012
0.097257003	0.622806013	0.677536011
0.583205998	0.120079003	0.185534000
0.087187000	0.380645007	0.312844008
0.603794992	0.879299998	0.821545005
0.688597023	0.909952998	0.375315994
0.184559003	0.413607001	0.868131995
0.323237985	0.897409022	0.624387026
0.822755992	0.411058009	0.132275999
0.685100019	0.100451998	0.631120026
0.186542004	0.593142986	0.110380001
0.319660008	0.104359001	0.377572000
0.810492992	0.593765020	0.877709985
0.678273976	0.366104990	0.910495996
0.188866004	0.886795998	0.417479992
0.314139992	0.623447001	0.900099993
0.820146978	0.131265998	0.390733987
0.686151028	0.622539997	0.095440000
0.180536002	0.126799002	0.598541975
0.323051989	0.388902009	0.117371999

0.811260998	0.880329013	0.591293991
0.375627011	0.901494980	0.685954988
0.882857025	0.413617015	0.186467007
0.628904998	0.903324008	0.320852995
0.120242998	0.405344009	0.819305003
0.618264019	0.106204003	0.680782974
0.126211002	0.600596011	0.164136007
0.376650989	0.102590002	0.319815993
0.871062994	0.600893021	0.824671984
0.889135003	0.686693013	0.380488008
0.408230007	0.180981994	0.874855995
0.911876023	0.321359009	0.619141996
0.405838996	0.815204978	0.124526002
0.099421002	0.682835996	0.619320989
0.586860001	0.175632000	0.126528993
0.086139999	0.322254986	0.369011998
0.605443001	0.818540990	0.879077971
0.414712012	0.585763991	0.088048004
0.386622995	0.608412981	0.007554000
0.894913018	0.114543997	0.494987994
0.594343007	0.404992998	0.105398998
0.614907980	0.377261013	0.024025001
0.089047998	0.917051971	0.605933011
0.116741002	0.895824015	0.525318980
0.590440989	0.584295988	0.905920982
0.616316020	0.609818995	0.986069977
0.114208996	0.120977998	0.487100989
0.391494989	0.392899990	0.006905000
0.909174979	0.916688979	0.401890010
0.882040977	0.893324971	0.482488006
0.100900002	0.418534994	0.587288022
0.022049000	0.390141994	0.613368988
0.521804988	0.873149991	0.122892000
0.009471000	0.640110016	0.370310009
0.595377982	0.082997002	0.916706026
0.516552985	0.110216998	0.888639987
0.979483008	0.631730020	0.622777998
0.398005992	0.081240997	0.082254998
0.476074010	0.108827002	0.112508997
0.901300013	0.422401011	0.413657010
0.979040980	0.394308001	0.384287000
0.488673002	0.874504983	0.880905986
0.576512992	0.088064998	0.421241999
0.608425975	0.011088000	0.391873002

0.080600001	0.597172022	0.911114991
0.108551003	0.517587006	0.885215998
0.416624010	0.088345997	0.585169017
0.392057002	0.007900000	0.612421989
0.912293971	0.602283001	0.085194997
0.889131010	0.522138000	0.114381000
0.610319972	0.995037973	0.613484979
0.086383000	0.402745008	0.086594000
0.114946999	0.484100997	0.108101003
0.412075996	0.912877023	0.415625006
0.387459993	0.993902981	0.391039997
0.906650007	0.403584987	0.906858981
0.880424023	0.484674990	0.883544981
0.671774983	0.899891019	0.779204011
0.740702987	0.857239008	0.793651998
0.742407978	0.795687973	0.849597991
0.675395012	0.776344001	0.893499970
0.170545995	0.721574008	0.601608992
0.238076001	0.699349999	0.643099010
0.236283004	0.639033020	0.699398994
0.166333005	0.599802017	0.717356026
0.336934000	0.771418989	0.110464998
0.270051986	0.787827015	0.155775994
0.271102995	0.848020971	0.212954000
0.338880986	0.892473996	0.227302998
0.820483029	0.603977025	0.282786995
0.749643028	0.640236974	0.305390000
0.748748004	0.699624002	0.363516003
0.817789972	0.723376989	0.401795000
0.897203982	0.230159998	0.318428993
0.841306984	0.226147994	0.258358002
0.774869025	0.176552996	0.264807999
0.764396012	0.127241999	0.329694986
0.924230993	0.081559002	0.571739972
0.741328001	0.161172003	0.629736006
0.727908015	0.227200001	0.677042007
0.658779025	0.234622002	0.722310007
0.602582991	0.174652994	0.724533021
0.228055000	0.112723999	0.665274978
0.210300997	0.154415995	0.733897984
0.146459997	0.207231000	0.738358021
0.097661003	0.219733000	0.672966003
0.384519011	0.274116009	0.180965006
0.337071002	0.288574010	0.246656999

0.285800010	0.353917986	0.249038994
0.278456002	0.405268013	0.185083002
0.407564014	0.898047984	0.906706989
0.090414003	0.090079002	0.407842010
0.095068000	0.626039982	0.384930998
0.895882010	0.613263011	0.603022993
0.427046001	0.422219992	0.931335986
0.580892980	0.917786002	0.583297014
0.602265000	0.898163974	0.097428001
0.385836005	0.723743021	0.854623020
0.600606978	0.469512999	0.109794997
0.099889003	0.979825020	0.618494987
0.606317997	0.261770010	0.872749984
0.405272007	0.523087978	0.100876004
0.110037997	0.797388017	0.360760987
0.612877011	0.722239971	0.141506001
0.896871984	0.979171991	0.389973998
0.605741024	0.521969020	0.895781994
0.884507000	0.780447006	0.637363017
0.863955975	0.391566008	0.720315993
0.602141023	0.094264001	0.980332971
0.111702003	0.401401013	0.525738001
0.364286989	0.108029999	0.773998022
0.897572994	0.414310992	0.477921993
0.625465989	0.105632000	0.231786996
0.130437002	0.393000007	0.267297000
0.394356996	0.089449003	0.018018000
0.738894999	0.871618986	0.385805011
0.477250993	0.101666003	0.603308022
0.234695002	0.374464989	0.876459002
0.976679027	0.609715998	0.089263998
0.279724985	0.852676988	0.609673977
0.514244974	0.095475003	0.405429989
0.778437972	0.365619004	0.122386001
0.016491000	0.602858007	0.902689993
0.474846005	0.903842986	0.402386993
0.232991993	0.633449972	0.092341997
0.969585001	0.394432992	0.892938972
0.276333988	0.149721995	0.391413003
0.763880014	0.634720981	0.892572999
0.021956000	0.398966014	0.091943003
0.719421029	0.380553991	0.863211989
0.477851987	0.600013018	0.093962997
0.236671001	0.898752987	0.376313001

0.266851008	0.612164021	0.858503997
0.533917010	0.388024002	0.121420003
0.025427001	0.904591978	0.610684991
0.731528997	0.609917998	0.138815999
0.972813010	0.905327022	0.396378994
0.526453018	0.592637002	0.899483979
0.765079021	0.892021000	0.634277999
0.386294991	0.860466003	0.733963013
0.582957983	0.092859000	0.485671014
0.899253011	0.371401995	0.230757996
0.095498003	0.606589973	0.973617971
0.616917014	0.857828975	0.277395010
0.412025988	0.093116000	0.520682991
0.104593001	0.357502997	0.780228019
0.893267989	0.610682011	0.023596000
0.400985986	0.903545976	0.478729993
0.110003002	0.651126981	0.199388996
0.896863997	0.396293998	0.970938027
0.390132993	0.144724995	0.273761004
0.885775030	0.650138974	0.787235022
0.103597000	0.389883012	0.025212999
0.103909999	0.483395010	0.592585027
0.363389999	0.225305006	0.887835979
0.866298020	0.280131012	0.600884020
0.602527976	0.019637000	0.904789984
0.893217981	0.485246986	0.398635000
0.633235991	0.217895001	0.113433003
0.130485997	0.277520001	0.380721003
0.389640987	0.018402999	0.096589997
0.109618999	0.644325018	0.876416028
0.852213025	0.388830990	0.386341989
0.382768989	0.619809985	0.133919001
0.112627000	0.356936991	0.124681003
0.378713012	0.867219985	0.382876992
0.148300007	0.393393993	0.624499023
0.643342018	0.114338003	0.885501027
0.348847985	0.114146002	0.109609999
0.634002984	0.378870994	0.150114000
0.619489014	0.619309008	0.858965993
0.120085001	0.882075012	0.651535988
0.871912003	0.357396990	0.876159012
0.878984988	0.881667018	0.355720997
0.884615004	0.648810983	0.121671997
0.378500015	0.134700000	0.610951006

0.609138012	0.138116002	0.395103991
0.671495020	0.949248016	0.738071024
0.794712007	0.872964025	0.762181997
0.797743022	0.764250994	0.859718025
0.678645015	0.730856001	0.938739002
0.172983006	0.766993999	0.556366026
0.293671012	0.728941977	0.630874991
0.290628999	0.621845007	0.729330003
0.165560007	0.550825000	0.759267986
0.334394008	0.726683021	0.064595997
0.215766996	0.754854977	0.146140993
0.216893002	0.861069024	0.245422006
0.338073999	0.940759003	0.269450992
0.822640002	0.557322979	0.238463998
0.694849014	0.620176971	0.278537989
0.692462981	0.727086008	0.379869998
0.815976977	0.768808007	0.447228998
0.950550020	0.265165001	0.312913001
0.850565016	0.259808987	0.204594001
0.732218027	0.175635993	0.216499999
0.715483010	0.085460000	0.332338005
0.792888999	0.157988995	0.591598988
0.770901978	0.274906993	0.677634001
0.648899019	0.289231986	0.755159020
0.547134995	0.181043997	0.756693006
0.274484009	0.068348996	0.665459991
0.244599998	0.144741997	0.787518024
0.133307993	0.236098006	0.794171989
0.045681000	0.256774008	0.676558018
0.427331001	0.226186007	0.179056004
0.341544986	0.250034988	0.298022002
0.252516001	0.365750015	0.303113997
0.242699996	0.458490014	0.188389003
0.378147990	0.930776000	0.859099984
0.371452987	0.846506000	0.922600985
0.410010993	0.937146008	0.958862007
0.070087999	0.028431000	0.413289011
0.042222999	0.123737000	0.381096989
0.141232997	0.089165002	0.367056996
0.134031996	0.662227988	0.346697986
0.111648999	0.637921989	0.446781009
0.108969003	0.563574016	0.371699989
0.855684996	0.632701993	0.650089025
0.888216972	0.549013019	0.597414017

0.878132999	0.641220987	0.547766984
0.441518009	0.373760015	0.890609026
0.483150005	0.453011006	0.943771005
0.388567001	0.464632004	0.900565028
0.522687972	0.924589992	0.554811001
0.622605979	0.894261003	0.539722979
0.573814988	0.874472022	0.631223023
0.875527978	0.054409999	0.604973972
0.968433022	0.034745999	0.561803997
0.950589001	0.126908004	0.609744012
0.598703027	0.946004987	0.053096998
0.636488020	0.921180010	0.147669002
0.634766996	0.849031985	0.070027001
0.500970006	0.742986023	0.998759985
0.489122987	0.248604000	0.003011000
0.996312976	0.504965007	0.751824975
0.004017000	0.514689028	0.246681005
0.751111984	0.012280000	0.500856996
0.256054014	0.008432000	0.509350002
0.748969018	0.493286014	0.003054000
0.253917992	0.492547989	0.993054986
0.503045022	0.997780025	0.750186026
0.498903006	0.996123016	0.254738986
0.999688983	0.761523008	0.499433994
0.000113000	0.252914011	0.495160997

AE-ZIF:

1.0

16.9076995850	0.0000000000	0.0000000000
0.0000000000	16.9076995850	0.0000000000
0.0000000000	0.0000000000	16.9076995850

N	C	H	Co
56	120	128	12

Direct

0.419135988	0.671724975	0.966453016
0.934633970	0.173121005	0.448105991
0.577611029	0.316700995	0.982611001
0.081625998	0.838279009	0.477297008
0.582795024	0.670446992	0.027326001
0.076705001	0.182761997	0.536746025
0.419790000	0.324167013	0.050080001
0.913447022	0.832970023	0.524900973
0.976258993	0.423354000	0.670765996
0.480242014	0.906369984	0.181272000
0.970550001	0.598733008	0.315880001
0.465631008	0.069306999	0.836299002
0.024106000	0.588146985	0.679279983
0.513863027	0.074722998	0.178747997
0.014870000	0.429159999	0.324070990
0.534138978	0.915012002	0.815903008
0.670237005	0.968591988	0.421611011
0.179957002	0.488373011	0.910782993
0.333198994	0.965618014	0.577961981
0.828674018	0.474283993	0.083066002
0.681964993	0.031295002	0.583432972
0.182255000	0.518827021	0.075414002
0.322522014	0.034070000	0.423999995
0.817065001	0.524524987	0.916067004
0.675499976	0.406560987	0.983749986
0.181133002	0.925580025	0.486865014
0.327966988	0.575483978	0.965727985
0.828944981	0.089841999	0.459803998
0.680261016	0.581257999	0.023376999
0.179670006	0.095716998	0.527940989
0.331472009	0.426755995	0.049082000
0.815629005	0.921675026	0.517147005
0.417896986	0.974385023	0.679159999
0.923928976	0.481557012	0.174419999
0.578010023	0.967288017	0.326380998

0.073155001	0.473022997	0.833701015
0.579653025	0.031849999	0.671958983
0.084201001	0.533568978	0.163123995
0.417432010	0.033546001	0.331367999
0.907868981	0.523190022	0.818835020
0.966502011	0.691694975	0.413987994
0.478623986	0.172948003	0.917931020
0.985679984	0.328889996	0.578554988
0.476792991	0.811842024	0.084486000
0.025745001	0.687093019	0.587146997
0.516524971	0.167821005	0.084852003
0.010164000	0.330285996	0.411886007
0.532227993	0.815357029	0.907070994
0.904690981	0.413138986	0.911122978
0.413314015	0.890132010	0.886533022
0.096892998	0.110501997	0.415053993
0.088068001	0.623672009	0.389901996
0.906948984	0.611796975	0.604219019
0.413419992	0.406987995	0.934284985
0.591095984	0.922110975	0.585731983
0.593246996	0.892265022	0.098228000
0.373133987	0.680546999	0.898989975
0.888001978	0.181243002	0.380261004
0.620181978	0.309774995	0.912679017
0.126696005	0.833662987	0.408838987
0.627843976	0.678775012	0.095657997
0.115576997	0.182199001	0.609530985
0.378502995	0.323747009	0.121578999
0.866708994	0.825511992	0.591935992
0.908047020	0.378100991	0.673672974
0.404641002	0.872364998	0.181320995
0.892395020	0.626164019	0.322189003
0.400597006	0.118185997	0.826197028
0.100951999	0.619503975	0.676621974
0.583294988	0.117292002	0.186939001
0.081491999	0.382586986	0.314244002
0.609363019	0.881202996	0.818044007
0.682287991	0.902052999	0.375079989
0.186850995	0.416725993	0.870648026
0.324178994	0.898944020	0.624342978
0.825293005	0.406769991	0.129780993
0.685957015	0.101865001	0.627421021
0.189749002	0.592666984	0.109568000
0.315200001	0.102294996	0.378910005

0.809934020	0.594893992	0.873520017
0.679547012	0.364789009	0.913082004
0.187727004	0.886834979	0.414894998
0.317279994	0.622043014	0.898810983
0.821819007	0.130948007	0.388357013
0.687745988	0.624529004	0.092923999
0.179519996	0.128162995	0.604589999
0.325628996	0.388453990	0.122171000
0.806607008	0.879697978	0.587146997
0.375791013	0.904235005	0.686635017
0.883482993	0.411401004	0.185783997
0.626031995	0.901409984	0.316848993
0.121229999	0.407624990	0.823993981
0.620890975	0.103602000	0.679906011
0.129697993	0.601638973	0.163468003
0.373219997	0.101774000	0.322403997
0.864759982	0.594088018	0.814061999
0.890146017	0.683609009	0.382822990
0.408192009	0.181608006	0.876024008
0.913829029	0.320019990	0.617356002
0.402936012	0.813243985	0.121974997
0.101641998	0.681138992	0.620262027
0.585058987	0.174311996	0.129217997
0.078726001	0.321707994	0.367657989
0.608312011	0.819405973	0.874514997
0.417539001	0.586050987	0.087232001
0.390287012	0.608087003	0.006300000
0.897566020	0.115764998	0.492532015
0.591454983	0.404307008	0.105168998
0.613170981	0.376228005	0.024466000
0.088275000	0.917437017	0.603317022
0.115759000	0.895422995	0.522741973
0.588174999	0.582228005	0.906457007
0.615574002	0.609930992	0.985414028
0.116332002	0.128509998	0.491566002
0.390262008	0.387663990	0.009840000
0.910098016	0.915318012	0.401131988
0.881137013	0.892027020	0.480958015
0.101678997	0.420924008	0.588485003
0.021633999	0.392334014	0.611603975
0.519393027	0.869816005	0.121304996
0.010721000	0.637992024	0.373264015
0.590861976	0.076862998	0.919224977
0.511546016	0.104239002	0.893022001

0.983165979	0.628777981	0.623257995
0.398539990	0.076993003	0.084275998
0.475571990	0.105645001	0.115231000
0.895519018	0.425356001	0.414016992
0.972972989	0.396021992	0.384452999
0.491602987	0.875037014	0.871169984
0.576032996	0.083878003	0.421741009
0.606215000	0.006868000	0.391005993
0.079554997	0.598448992	0.914002001
0.109958000	0.520384014	0.887567997
0.417144001	0.089703999	0.584151030
0.391424000	0.010087000	0.612100005
0.912344992	0.598874986	0.083090000
0.890043974	0.518531024	0.111451000
0.616726995	0.993055999	0.612160981
0.089056998	0.404201001	0.086076997
0.117273003	0.484701008	0.107490003
0.411918014	0.912976027	0.418172002
0.385376990	0.993378997	0.393658012
0.877420008	0.483862013	0.881789982
0.678654015	0.902756989	0.779422998
0.747148991	0.859687984	0.796253026
0.746469021	0.797985017	0.851619005
0.677196026	0.777634025	0.892036974
0.171233997	0.722419024	0.604265988
0.239951998	0.700414002	0.644445002
0.239473999	0.637651980	0.698217988
0.170133993	0.596083999	0.715044975
0.333517998	0.771094024	0.107354999
0.266214013	0.788896024	0.152159005
0.267637014	0.848429978	0.209698007
0.336914986	0.891153991	0.224653006
0.823584020	0.603936970	0.282362998
0.751946986	0.638963997	0.305350006
0.749849021	0.695769012	0.365586013
0.818889022	0.719428003	0.404305011
0.899891019	0.224314004	0.310743988
0.841072977	0.221149996	0.253241003
0.772970974	0.174637005	0.263206989
0.763782978	0.126935005	0.329768986
0.927456021	0.082592003	0.568843007
0.739305973	0.164718002	0.624750972
0.724249005	0.230123997	0.672885001
0.656647027	0.234099999	0.720588982

0.603555024	0.170791000	0.724641979
0.226209998	0.112043999	0.670790970
0.208162993	0.151021004	0.741380990
0.144651994	0.203999996	0.746272981
0.097356997	0.219952002	0.680649996
0.384133995	0.272821993	0.186042994
0.337069005	0.289279997	0.251825988
0.288657010	0.356301993	0.254527003
0.282622010	0.407640010	0.190074995
0.383803010	0.728241026	0.857441008
0.597546995	0.468957990	0.108942002
0.099917002	0.980268002	0.614787996
0.604691029	0.265085995	0.869576991
0.407582998	0.523514986	0.099495001
0.110784002	0.794736981	0.360531986
0.612333000	0.721678972	0.140743002
0.897533000	0.977792978	0.389380008
0.603636026	0.519748986	0.898600996
0.880769014	0.783149004	0.637880027
0.860069990	0.392316997	0.714044988
0.599016011	0.088127002	0.982501984
0.114335001	0.403577000	0.527218997
0.355661005	0.104373001	0.782727003
0.891326010	0.418377995	0.478549987
0.625612020	0.102565996	0.232789993
0.126344994	0.396288007	0.270738006
0.395873994	0.086002000	0.020021999
0.730382979	0.861315012	0.386642009
0.478430986	0.100899003	0.601768970
0.237507001	0.378167987	0.878665984
0.976669014	0.607195020	0.086237997
0.280768007	0.854349017	0.609268010
0.513548970	0.091654003	0.407283008
0.781804979	0.361128002	0.118473999
0.015340000	0.601306021	0.904608011
0.474281013	0.904787004	0.402680010
0.236194000	0.633076012	0.091903999
0.269556999	0.145518005	0.391496003
0.766619980	0.639200985	0.890676975
0.024559001	0.399569005	0.091820002
0.723870993	0.379004002	0.868848979
0.480257988	0.599985003	0.094648004
0.234817997	0.900601983	0.373640001
0.269749999	0.610318005	0.857671976

0.530717015	0.387735993	0.120319001
0.024743000	0.905743003	0.609444976
0.734748006	0.612709999	0.134757996
0.973915994	0.904865026	0.396806002
0.524096012	0.589774013	0.900591016
0.759114981	0.892379999	0.628320992
0.385125011	0.864385009	0.735978007
0.583283007	0.087030001	0.486097008
0.899874985	0.371740013	0.233498007
0.092496999	0.608904004	0.976873994
0.615835011	0.859481990	0.269479990
0.412699997	0.094401002	0.519704998
0.105485998	0.360058010	0.784217000
0.892620027	0.608174026	0.022058001
0.404309988	0.903832972	0.481711000
0.113508999	0.652177989	0.200165004
0.869782984	0.388173014	0.953343987
0.387080997	0.143372998	0.275956988
0.878979981	0.638969004	0.770083010
0.104978003	0.390998006	0.024371000
0.104980998	0.485231012	0.593258023
0.368557990	0.231227994	0.885692000
0.872400999	0.273658991	0.601436019
0.598510027	0.013679000	0.907494009
0.888467014	0.487787992	0.398806006
0.630177975	0.217721999	0.115184002
0.119093001	0.272729993	0.377079010
0.390989989	0.013820000	0.097099997
0.106849000	0.647036016	0.879851997
0.846004009	0.392286003	0.387380004
0.385044008	0.620350003	0.132131994
0.116021998	0.358065009	0.123671003
0.377947003	0.866658986	0.387268007
0.147889003	0.395264000	0.626475990
0.637440979	0.109180003	0.887049019
0.348648995	0.109122999	0.111112997
0.630948007	0.379040986	0.150516003
0.616324008	0.615858972	0.858282030
0.119212002	0.882939994	0.649164021
0.928870022	0.374722987	0.871985018
0.881200016	0.880029023	0.354571998
0.885186017	0.645478010	0.119946003
0.380290002	0.137153998	0.609597027
0.608119011	0.134565994	0.396140993

0.679351985	0.952657998	0.738996983
0.802419007	0.874723017	0.766223013
0.801253021	0.765827000	0.863514006
0.677456975	0.731324971	0.936592996
0.172180995	0.769922018	0.561060011
0.294921011	0.731670022	0.633076012
0.294732004	0.619916022	0.726482987
0.171180993	0.545602024	0.755250990
0.331160992	0.726836979	0.061120000
0.211133003	0.757045984	0.141068995
0.213275999	0.862419009	0.241882995
0.337065995	0.938974023	0.267410010
0.825336993	0.559361994	0.236153007
0.696712017	0.620757997	0.277099013
0.693482995	0.721872985	0.383235991
0.817134023	0.764653981	0.449999005
0.953613997	0.258453012	0.302004009
0.848329008	0.255369991	0.199191004
0.727639019	0.175937995	0.217371002
0.714298010	0.086314999	0.336986005
0.790114999	0.163039997	0.585210025
0.764746010	0.280124992	0.672407985
0.645694017	0.288015991	0.754087985
0.549691021	0.173886999	0.759981990
0.273988008	0.069112003	0.667900026
0.242828995	0.139783993	0.794447005
0.131577000	0.232407004	0.802573979
0.046805002	0.259256989	0.685006976
0.425334007	0.223486006	0.184226006
0.339064986	0.250140011	0.302778006
0.255773008	0.368445992	0.308521986
0.247454002	0.461380988	0.192567006
0.395613998	0.946153998	0.872794986
0.393220007	0.869421005	0.939599991
0.039671000	0.122556001	0.400409997
0.119747996	0.058685001	0.394713998
0.119681001	0.587572992	0.354068995
0.118194997	0.662693024	0.424198985
0.877717972	0.646066010	0.564534009
0.875486016	0.576479971	0.641039014
0.468881011	0.388985008	0.918545008
0.398876011	0.462936014	0.917223990
0.620112002	0.893710017	0.541590989
0.540992975	0.898967981	0.609022975

0.878436983	0.054118998	0.600367010
0.972763002	0.036892001	0.558974028
0.954463005	0.128151998	0.606178999
0.621877015	0.932356000	0.132513002
0.626053989	0.855829000	0.063507996
0.503889978	0.744570017	0.994320989
0.489890993	0.246602997	0.004472000
0.998025000	0.501926005	0.750528991
0.005612000	0.512888014	0.246078998
0.748996019	0.010467000	0.493286014
0.254420012	0.010416000	0.512032986
0.750100970	0.494130999	0.002395000
0.256628990	0.491926014	0.993929982
0.502228022	0.003417000	0.751257002
0.492619008	0.992372990	0.255383015
0.998772025	0.759926975	0.501305997
0.999122977	0.253580004	0.495624989
